# Tubulin E-Hook Hexamers Reveal Sequence-Dependent Compaction and Transient Secondary Structure Signatures

**DOI:** 10.3390/molecules31193378

**Published:** 2026-09-23

**Authors:** Alexander C. Bromley, Nicholas A. Kruse, Connor R. Brower, Madison K. Beam, Nathan I. Hammer, Ryan C. Fortenberry, Dana N. Reinemann

**Affiliations:** 1 Department of Biomedical Engineering, University of Mississippi, University, MS 38677, USA; 2 Department of Chemistry and Biochemistry, University of Mississippi, University, MS 38677, USA; 3 Department of Chemical Engineering, University of Mississippi, University, MS 38677, USA

**Keywords:** tubulin, E-hook, peptide, vibrational spectroscopy, computational chemistry, Raman, DFT

## Abstract

The present work shows that E-hook fragments possess functional structural differences governed by electrostatic interactions and sequence composition. The acidic C-terminal tails of tubulin, known as E-hooks, play a central role in regulating interactions between microtubules and motor proteins, microtubule-associated proteins, and enzymatic modifiers. Despite their functional importance, the intrinsic structural properties of these peptide segments remain poorly characterized due to their intrinsically disordered nature. In this work, we present quantum-mechanically optimized structures of hexamer peptides derived from β-tubulin E-hook sequences. Density functional theory calculations were used to optimize peptide geometries using progressively larger basis sets. From the optimized geometries, we calculated theoretical Raman spectra, Ramachandran backbone dihedral distributions, and measured radii of gyration to resolve composition-dependent structural tendencies. The combined Raman and conformational analyses provide a systematic computational approach for comparing simulated and experimental Raman spectra of tubulin E-hooks and other intrinsically disordered proteins and offer insight into how E-hooks contribute to the recognition mechanisms underlying the tubulin code.

## 1. Introduction

Microtubules are dynamic cytoskeletal polymers that perform essential cellular functions including intracellular transport, chromosome segregation during mitosis [1], motility [2], and other stages of cellular evolution [3]. These polymers are composed of α- and β-tubulin heterodimers [4,5] that assemble into protofilaments which join to form the hollow cylindrical microtubule lattice [6]. Although the structured domains of the tubulin core have been extensively characterized through X-ray crystallography and cryo-electron microscopy [7], the C-terminal tails of tubulin remain largely unresolved in structural studies [8].

Microtubule C-terminal tails, commonly referred to as E-hooks, are short peptide segments rich in glutamic acid residues, giving them their namesake, and are characterized by high negative charge density [9]. E-hooks extend outward from the microtubule surface, acting like an electronegative brush for motor proteins, microtubule-associated proteins (MAPs), and severing enzymes, affecting their binding capabilities and functionality [10,11,12]. E-hooks are thought to be modulated by two major structural factors that influence MAPs: isotype expression [13,14] and post-translational modification [15,16]. The functional importance of E-hooks and the regulatory function of their structure have led to the concept of the “tubulin code,” wherein the combinations of the tubulin isotypes and post-translational modifications regulate microtubule behavior and function by modulating MAP interactions [17,18].

Despite their biological significance, the structural properties of E-hooks remain poorly understood. Because of their intrinsic flexibility from the electrostatic repulsion between their acidic residues, these segments are widely believed to behave as intrinsically disordered peptides, making traditional experimental structure studies, such as X-ray crystallography and cryo-electron microscopy, ineffective [19]. However, growing evidence from alternative structural techniques suggests that intrinsically disordered regions may still exhibit transient local secondary structures that may contribute to molecular recognition [20]. Novel approaches, such as computational chemistry, must be employed in order to examine how the structures of these molecules influence their environments.

E-hook structures have been studied in the past using molecular dynamics (MD) [8,20,21,22]. Conformational ensembles were originally prepared by allowing a free E-hook to explore its conformational space. While the E-hooks did portray the predicted characteristics of intrinsically disordered proteins, the MD simulations predict that they also demonstrated a consistent yet transient helical secondary structure [20]. Further MD studies with E-hooks attached to tubulin’s globular core have revealed the existence of anchoring points, wherein certain E-hook residues may non-covalently interact with the globular core [21]. These specific residues of the E-hook and globular core that interacted as attachment points varied between simulations yet remained in place throughout each simulation, suggesting that E-hooks may have their conformational landscape limited in some capacity [8]. Other computational studies have used MD to examine how E-hooks affect MAPs and motor protein binding. These computations have revealed a soft, guided landing functionality of E-hooks [23,24]. By providing a broad brush of negative charge, domains with positive residues are drawn toward the microtubule, but upon nearing the filament, the microtubule-binding domain (usually along the globular core) begins to control the MAP’s microtubule binding due to its stronger binding affinity [23,25]. While MD can show how E-hooks may change over time and how they may affect dynamic processes, minute differences in intramolecular interactions between the different E-hook isotypes cannot be effectively predicted, preventing deeper understanding of how E-hook structure affects microtubule functionality [26].

Time-independent quantum-chemical modeling provides the precision necessary to study intramolecular interactions leading to these transient secondary structures and how they differ across E-hook sequence composition and post-translational modification. However, the increased cost of this approach prevents its use across a conformational ensemble [27]. Density functional theory (DFT) computations can both optimize a given molecular geometry and predict its associated vibrational frequencies, allowing theoretical spectra to be compared directly to experimental measurements [28]. Raman spectroscopy, on the other hand, provides an experimental means to probe peptide structure in flexible, biological systems [29]. This technique is particularly useful in the study of E-hooks due to backbone amide and side-chain vibrational modes providing detailed information about peptide conformation and hydrogen bonding, allowing for experimental corroboration with computationally derived models [29,30,31,32,33].

In this study, quantum-mechanically optimized structures of E-hook hexamer peptides are presented. These are derived from the C-terminal of β-tubulin sequences (Table 1) and are used to quantify each simulated geometry’s agreement with experiment as well as map E-hook Raman fingerprints. The C-terminal hexamers of the E-hooks are used as representative peptides due to the computational cost of quantum-mechanical calculations [34,35]. In addition, this region of the E-hook hosts the major compositional differences between the isotypes and would likely be the portion closest to MAPs and motor proteins attempting to bind to the microtubule, making their conformational information relevant to functional translation [9,19,36]. Our approach integrates DFT structural optimizations, theoretical Raman spectra calculations, Ramachandran backbone analysis, and structural compactness measurements through radius of gyration calculations. By combining these methods, the aim of the present work is to characterize intrinsic structural tendencies of E-hooks and examine how electrostatics and sequence composition influence their conformational behavior.

## 2. Results and Discussion

### 2.1. Raman Spectra of E-Hook Peptides

As all calculated spectra represent isolated gas-phase E-hook structures, they do not fully reproduce the molecular environment present within the experimental samples. In particular, the absence of solvent dielectric screening may enhance intramolecular electrostatic interactions and repulsion between charged residues. The lack of surrounding water molecules or neighboring peptides eliminates solvent-mediated intermolecular interactions and packing effects. These forces may alter both the optimized conformations and their associated vibrational frequencies relative to those present experimentally [38]. Furthermore, intermolecular interactions and packing in the solid-state samples may contribute to shifts, broadening, and alterations in Raman intensities that cannot be captured by the isolated molecule calculations. Therefore, agreement between calculated and experimental spectra is interpreted primarily in terms of correspondence between characteristic vibrational features and relative peak positions rather than as evidence that the optimized structures uniquely represent the conformational ensemble present experimentally.

Despite these limitations, the similarity of the experimentally measured E-hook Raman spectra in aqueous solution and in the solid state indicates that the major spectral features are retained across substantially different intermolecular environments [34,35]. This suggests that many of the prominent Raman bands arise predominantly from local molecular vibrations that persist across these environments. Nevertheless, agreement between the two experimental conditions does not eliminate the effects of solvation or intermolecular interactions relative to an isolated gas-phase calculation, and smaller differences in peak position, breadth, and intensity may still arise from these effects. Thus, the dynamic time warping (DTW) analysis is conducted for each calculated spectrum to accommodate modest differences in peak position between the calculated and experimental spectra while preserving the overall spectral relationships, thereby facilitating comparison and characterization of vibrational features within the E-hooks, which can be seen by increased Pearson’s coefficient for DTW-analyzed spectra (Table 2).

Across all β-tubulin isotypes, both the 4+2 and 2+4 buildup methods reproduce the majority of experimentally observed spectral features. However, neither the 4+2 nor the 2+4 reconstruction strategy consistently outperforms the other across all sequences. Instead, the preferred reconstruction strategy is strongly dependent upon peptide composition, indicating that sequence-specific structural interactions may influence the preservation of experimentally observed vibrational behavior (Table 2).

#### 2.1.1. βI—EAEEEA

βI E-hooks (EAEEEA) demonstrate the strongest sensitivity to reconstruction strategy. The EAEE+EA (4+2) buildup reduces the normalized peak-shift RMSD from 0.46 to 0.27 while simultaneously improving the Pearson correlation from 0.215 to 0.297. The peak-matching fraction remains identical between both geometries, indicating that the improvement arises from more accurate reproduction of experimentally observed vibrational frequencies rather than recovery of additional spectral features. In the EAEE+EA spectrum, the amide III region near 1250–1260 cm^−1^ closely reproduces the experimental feature (Figure 1) and is assigned primarily to amide III-like C–N stretching coupled with N–H bending, reflecting improved preservation of backbone vibrational modes. Likewise, the amide I band near 1650–1660 cm^−1^, assigned to backbone carbonyl stretching, shows close frequency agreement with experiment. Despite these improvements, the simulation continues to overestimate the intensity of the 1290–1310 cm^−1^ feature, which is assigned largely to amide III-like and mixed collective vibrations, indicating that vibrational coupling among the glutamate-rich residues remains incompletely captured.

The alternative EA+EEEA (2+4) reconstruction predicts effectively the same principal amide III and amide I assignments but exhibits greater splitting and intensity distortion throughout the 780–820 cm^−1^ region. This span of features corresponds primarily to skeletal bending/torsional and mixed collective vibrations. Most notably, both reconstructions fail to reproduce the broad experimental band between approximately 1050 and 1100 cm^−1^, despite reproducing neighboring features. This region is associated with mixed collective backbone and side-chain motions, suggesting that the experimentally observed vibration likely depends on longer-range coupling or conformational averaging that is disrupted by fragmentation. Consequently, although the EAEE+EA (4+2) reconstruction substantially improved peak-position agreement, the persistent absence of the 1050–1100 cm^−1^ feature explains why the overall Pearson correlation remains comparatively low.

#### 2.1.2. βII—EGEDEA

The EG+EDEA (2+4) reconstruction provides a more useful description of the βII E-hooks (EGEDA) even though it produces lower composite scores, and it maintains strong agreement with the experimental Raman spectrum. Although both reconstruction pathways reproduce the dominant experimental features, the 2+4 model more consistently preserves peak positions throughout the fingerprint region, indicating that retaining the larger C-terminal fragment better maintains the local vibrational environment. In the EG+EDEA (2+4) spectrum, the amide III band near 1240–1250 cm^−1^ closely matches the experimental feature (Figure 2) and is assigned primarily to C–N stretching coupled with N–H bending from the vectors of the quantum-chemically computed normal modes. Such an assignment is consistent with preservation of backbone amide vibrations. Likewise, the dominant 1450 cm^−1^ feature, assigned to CH_2_/CH_3_ deformation (scissoring/bending), reproduces both the position and relative intensity of the experimental maximum. The amide I region near 1660 cm^−1^, arising predominantly from backbone carbonyl stretching, also shows excellent positional agreement, indicating that the principal peptide backbone vibrations are retained despite intensity differences.

The alternative EGED+EA (4+2) reconstruction reproduces the same principal vibrational assignments but exhibits greater splitting within the 780–820 cm^−1^ region, where mixed collective and skeletal bending/torsional vibrations generate additional simulated peaks that have not been observed experimentally. Additionally, EGED+EA is predicted to exhibit exaggerated relative intensity compared to experiment. Similarly, the 1300–1350 cm^−1^ region contains several partially unmatched bands, suggesting increased perturbation of coupled backbone vibrations, which is not present in the experimental spectrum, potentially due to the broadening effect of vibrational mode mixing.

#### 2.1.3. βIII—EAQGPK

βIII E-hooks (EAQGPK) exhibit the strongest overall agreement with experiment, indicating that βIII’s structural behavior is comparatively robust to fragmentation, and favor the fragmentation strategy of EAQG+PK (4+2). The EAQG+PK reconstruction produces superior composite scores and stronger global spectral agreement relative to EA+QGPK. In the EA+QGPK spectrum, the dominant feature near 1445–1455 cm^−1^ agrees strongly with the experimental maximum (Figure 3) and was assigned primarily to CH_2_/CH_3_ deformation (scissoring/bending), reproducing both its position and relative prominence. The amide I region between 1650 and 1700 cm^−1^ also closely matches the experimental spectrum and is attributed to backbone carbonyl stretching. A remaining discrepancy occurs in the 780–820 cm^−1^ region, where skeletal bending/torsion and mixed collective vibrations are somewhat over-resolved, producing several closely spaced simulated peaks that exceed the complexity observed experimentally.

The EAQG+PK (4+2) reconstruction likewise reproduces the dominant amide I and CH_2_ deformation bands and provides a stronger overall spectral-shape agreement than EA+QGPK. However, the 1240–1260 cm^−1^ amide III region, assigned predominantly to C–N stretching coupled with N–H bending, exhibits broader splitting and slightly poorer positional agreement than in the 2+4 reconstruction. Additional unmatched features between 1300 and 1350 cm^−1^ suggest increased fragmentation-induced coupling among neighboring backbone modes despite preservation of the underlying vibrational assignments. Consequently, while both fragmentation pathways retain the major vibrational characteristics of βIII, the composite analysis supports EAQG+PK (4+2) as the preferred reconstruction, rather than the EA+QGPK (2+4) structure.

#### 2.1.4. βIV—AEEEVA

βIV E-hooks (AEEEVA) favor the AEEE+VA (4+2) geometry. The peak-matching fraction remains identical between both structural models, while peak-shift RMSD, Pearson’s correlation, and composite scoring metrics all improve under the 4+2 reconstruction. These improvements indicate moderately enhanced reproduction of vibrational frequencies without significant changes in spectral coverage. In the AEEE+VA spectrum, the amide III region near 1240–1260 cm^−1^ exhibits close agreement with the experimental spectrum (Figure 4) and is assigned primarily to amide III-like C–N stretching coupled with N–H bending, with dominant contributions from the contiguous glutamate residues within the AEEE fragment. The strong amide I band near 1660–1670 cm^−1^ is also accurately reproduced and corresponds predominantly to backbone and glutamate carbonyl stretching, indicating preservation of the principal peptide vibrational framework. A minor discrepancy remained near 1450–1470 cm^−1^, where several calculated CH_2_/CH_3_ deformation modes are more distinctly resolved than in the experimental spectrum, producing slight intensity redistribution despite good positional agreement.

In contrast, the AE+EEVA (2+4) reconstruction generates a broader cluster of unmatched features between 1300 and 1360 cm^−1^, reflecting increased splitting of modes associated with amide III and mixed collective backbone vibrations. The 800–850 cm^−1^ region, assigned primarily to skeletal bending/torsional and mixed collective vibrations, also appears more intense than observed experimentally, reflecting altered vibrational coupling after fragmentation. Although both reconstructions reproduce the dominant amide I feature well, the improved agreement throughout the fingerprint region demonstrates that preserving the contiguous AEEE segment more effectively maintains the experimentally observed vibrational behavior.

#### 2.1.5. βV—EEEIDG

βV E-hooks (EEEIDG) exhibit a metric-dependent response to the fragmentation strategy, with EEEI+DG (4+2) providing improved peak-position agreement while EE+EIDG (2+4) provides greater global spectral-shape correlation. The normalized peak shift RMSD decreases from EE+EIDG’s 0.48 to EEEI+DG’s 0.38, resulting in a lower position composite score for the 4+2 reconstruction. In contrast, the Pearson correlation decreases from 0.228 to 0.146, resulting in a lower Shape composite score for EE+EIDG. Because both reconstructions exhibit the same experimental peak-match fraction, these differences primarily reflect a trade-off between peak-position and global spectral-shape agreement.

In the EEEI+DG spectrum, several regions demonstrate the improved positional correspondence reflected by the position score. The amide III region near 1250–1300 cm^−1^ shows notably improved agreement with the experimental spectrum (Figure 5) and is assigned primarily to amide III-like C–N stretching coupled with N–H bending involving the glutamate-rich EEEI fragment, as shown in the quantum-chemical computations. Likewise, the strong feature near 1625–1635 cm^−1^ closely reproduces the experimental amide I band and is dominated by carbonyl stretching from glutamate carboxylate groups, indicating preservation of the backbone and acidic side-chain vibrational environment. A remaining discrepancy occurs near 1300–1330 cm^−1^, where the quantum-chemical computation produces an overly intense, partially unmatched band despite the correct underlying amide III assignment, suggesting altered coupling between neighboring glutamate residues following fragmentation.

In contrast, the EE+EIDG (2+4) reconstruction yields the higher Pearson correlation and correspondingly lower Shape composite score, indicating better global spectral-shape agreement despite its larger peak-shift RMSD. This reconstruction generates an overly pronounced feature near 800–850 cm^−1^, corresponding to mixed collective CH_2_-dominated vibrations, that substantially exceeds the experimental intensity. The amide III region around 1250–1300 cm^−1^ is also reproduced but exhibits broader splitting and greater intensity distortion than observed experimentally. Although the amide I feature near 1700 cm^−1^, assigned to carbonyl stretching, remains well positioned in both reconstructions, the composite analysis does not identify a reconstruction that is uniformly superior across all spectral descriptors. Instead, βV exhibits a fragmentation-dependent tradeoff: EEEI+DG more accurately reproduces experimental peak positions, whereas EE+EIDG provides better global spectral-shape agreement. The slightly lower balanced score for EEEI+DG provides modest overall support for the 4+2 reconstruction, but this preference is less robust than for isotypes whose reconstruction preference is retained across all three scoring schemes.

#### 2.1.6. βVI—PEDKGH

βVI E-hooks (PEDKGH) favored the PE+DKGH (2+4) reconstruction strategy. While both structures produce similar peak-matching fractions, lower composite scores and improved correlation metrics consistently favor the 2+4 reconstruction. In the PE+DKGH spectrum, the strong simulated feature near 1280–1300 cm^−1^ aligns well with the corresponding experimental band and is assigned primarily to amide III-like C–N stretching/N–H bending, with substantial contributions from the histidine-containing DKGH fragment. Likewise, the simulated carbonyl-region feature near 1680–1700 cm^−1^ closely reproduces the experimental peak and is associated predominantly with aspartic acid carboxylic acid C=O stretching, indicating that the 2+4 reconstruction retains residue-localized acidic-group vibrations. A less satisfactory feature occurs near 1350–1400 cm^−1^, where the simulation predicts several intense, partially unmatched peaks arising from mixed histidine N–H/C–N motions and backbone deformation, indicative of over-resolution or altered coupling of closely spaced modes.

The PEDK+GH spectrum also reproduces the dominant experimental band near 1430–1450 cm^−1^, but the simulation resolved it into two exceptionally intense peaks. The PEDK mode assignments associate these features primarily with histidine-localized N–H and C–N motions. The relatively intense simulated feature near 1730–1750 cm^−1^, assigned largely to amide-I/carbonyl stretching, is also stronger and more sharply defined than the corresponding experimental region (Figure 6). Compared with βI and βV, however, βVI demonstrates comparatively limited sensitivity to fragmentation, which may indicate more localized preservation of vibrational behavior across both reconstruction pathways.

### 2.2. Structural Compactness Corresponds with Raman Reconstruction Preferences and Sequence

To characterize the global structural compactness across β-tubulin E-hook isotypes, the R_g_ (Equation (4)) is calculated for optimized peptide structures across four progressively larger basis sets (Table 3). R_g_ values vary substantially between isotypes and reconstruction strategies, indicating significant sequence-dependent differences in structural compactness.

βIII E-hooks and βVI’s 2+4 reconstruction exhibit the lowest radii of gyration across all basis sets, with values remaining approximately between 4.08–4.65 Å and 4.41–4.44 Å, respectively. In contrast, βI, βII, and βV E-hooks produce substantially larger R_g_ values ranging from approximately 4.79–6.79 Å, indicating significantly more extended structures.

Several isotypes exhibit basis set-dependent structural expansion. βVI’s 4+2 demonstrates the largest increase in Rg, increasing from 5.06 Å under the minimal 3-21G basis set to 5.63 Å via 6-311+G(2df,2pd). βII’s 4+2 geometry similarly increases from 6.44 Å to approximately 6.79 Å. In contrast, βIII’s and βVI’s 2+4 structures show minimal variation across basis sets, indicating comparatively rigid structural behavior. The consistently lower Rg values observed in βIII’s and βVI’s 2+4 geometries may reflect increased intramolecular stabilization arising from charged and polar residue interactions, particularly involving lysine and histidine residues. Conversely, glutamate-rich sequences such as βI and βV appear to adopt more extended geometries, likely due to electrostatic repulsion among negatively charged side-chains. These results suggest that sequence-dependent structural compactness strongly influences vibrational behavior and may partially explain the distinct reconstruction preferences observed during Raman spectral analysis.

Additionally, the change in Rg primarily happens in the progression from 3-21G to 6-31G, and most isotypes exhibit a minor change in the transition from the 6-31+G(d,p) to 6-311+G(2df,2pd) calculation. This may allow extensions of these E-hook structures, which are 18–24 amino acids at full length, to proceed by limiting the ballooning computational cost as basis set size increases.

### 2.3. Preferred Reconstructions Occupy Favorable Ramachandran Conformational Space

To determine whether Raman-derived structural preferences correspond to physically meaningful structural differences, backbone dihedral angles have been examined using Ramachandran analysis (Figure 7). The favored reconstruction strategies of each isotype consistently display more constrained φ and ψ angles, indicating greater localization within Ramachandran space. This relationship is most pronounced in βI and βV, which also demonstrate the largest Raman-derived differences between competing structural models. In both isotypes, the reconstruction that produces superior Raman agreement also generates noticeably tighter spacing of backbone φ and ψ angles, whereas the alternative reconstruction exhibits a broader spread across multiple dihedral regions. These structural differences parallel the Raman discrepancies observed between the competing reconstruction strategies, suggesting that conformational restriction is associated with improved spectral agreement in these isotypes. In contrast, βIII and βVI structures exhibit substantial overlap between the φ-ψ distributions generated by the two reconstruction strategies. Although preferred buildup methods for these two isotypes appear to be present, both of their 4+2 and 2+4 geometries show high agreement with experimental Raman spectra. This behavior is mimicked here in that both 2+4 and 4+2 geometries occupy similar Ramachandran space instead of occupying distinct backbone space.

The favored reconstruction methods that result in structures with better computational-to-experimental agreement tend to cluster within favorable areas of the Ramachandran chart. Several residues exist within the β-sheet/extended area of the graph, lying within φ −150° to −90° and ψ 90° to 180°. The higher-agreement dihedrals gather around the other β extended/Polyproline II conformational basin, which wraps across the ψ ±180° boundary. Only one dihedral resides in the right-handed α-helix portion, sitting between φ −90° to −30° and ψ −70° to 0°. Interestingly, unfavored reconstruction methods’ dihedrals localize around the left-handed α-helix favored area of the chart, φ 30° to 90° and ψ 0° to 90° [39,40]. Otherwise, unfavored reconstruction strategies frequently exhibit broader dihedral dispersion and increased occupation of less favorable dihedral regions (any area outside of the listed areas of favored secondary structure), supporting the interpretation that Raman spectral agreement reflects physically meaningful structural differences rather than numerical fitting artifacts.

### 2.4. Residue Propensities for Hydrogen Bonding May Drive Conformational Differences

Hydrogen bonding analysis has been performed to characterize local structural stabilization within each peptide fragment and determine whether hydrogen bonding patterns correlate with experimentally favored structural reconstructions (Table 4). Hydrogen bonding capacity varies substantially between E-hook isotypes. PE+DKGH is predicted to possess the largest total hydrogen bonding network with seven hydrogen bonds, followed by EEEI+DG and EA+QGPK with six hydrogen bonds (Figure 8). EA+EEEA, EGED+EA, and PEDK+GH showcase the weakest overall hydrogen bonding capacities with only three total hydrogen bonds each.

Across nearly all peptide reconstructions, interresidue bonding interactions (hydrogen bonds wherein the donor and acceptor atoms are a part of two different residues) substantially exceed intraresidue hydrogen bonds (hydrogen bonds wherein the donor and acceptor atoms are a part of the same residue), indicating that structural stabilization primarily arises through cooperative residue–residue interactions rather than isolated local stabilization. For example, EAEE+EA forms four total hydrogen bonds but only one intraresidue hydrogen bond, suggesting that distributed intermolecular interactions dominate structural organization.

Residue-level analysis demonstrates strong dependence on amino acid identity (Table 5). Glutamate contributes the largest total hydrogen bonding contribution with 54 total hydrogen bonds and 1.5 interresidue bonds per residue across all 6-311+G(2df,2pd) basis set hexamer structures, reflecting its dominant structural role within glutamate-rich E-hook sequences. Lysine exhibits the strongest per-residue hydrogen bonding capacity with 2.8 total hydrogen bonds per residue, each being an interresidue bond, pointing to a strong local stabilizing potential. Glutamine residues similarly demonstrate strong intermolecular stabilization, averaging 2.0 total hydrogen bonds per residue, each being an interresidue interaction.

Differences in residue-specific hydrogen bonding behavior likely contribute to the observed isotype-dependent fragmentation preferences. Peptides rich in strongly interacting charged and polar residues may preserve structural integrity differently depending on fragmentation boundaries and sequence, explaining why preferred reconstruction strategy varies across isotypes.

## 3. Materials and Methods

### 3.1. Peptide Structure Construction

Structures of E-hook hexamers corresponding to C-terminal regions of β-tubulin E-hook sequences are constructed. Ionizable side-chains are modeled in their charged states, with acidic glutamate and aspartate residues represented in their deprotonated, negatively charged forms and basic lysine residues represented in their protonated, positively charged forms. The N- and C-termini are modeled in their neutral forms to more closely establish the calculation as a continuation within the full-length tubulin protein rather than as an isolated, ionized peptide termini. Histidine residues are modeled using the Nδ1-H neutral tautomer. Each peptide is calculated in the gas phase and in isolation.

A stepwise buildup method for constructing E-hooks has been previously developed using quantum-chemical geometry optimizations [34,35] and is described briefly here. Each hexamer sequence is divided into two residue structures. These structures are iteratively optimized in Gaussian16 [41] using density functional theory (DFT) with the Becke three-parameter Lee–Yang–Parr (B3LYP) exchange-correlation functional [42,43,44] with basis sets increasing in size. Basis sets 3-21G [45], 6-31G [46], 6-31+G(*d*,*p*) [47], and 6-311+G [*2df*,*2pd*) [48] are utilized, with the last two incorporating diffuse and polarization functions to improve the description of intramolecular interactions and the diffuse electron environment of E-hooks, respectively. Upon optimization of the structure with the final basis set, the dimer is joined with another structurally optimized dimer, creating a tetramer that is then subjected to the same iterative optimization. Tetramers are formed from both the N-terminus and the C-terminus of the representative hexamer, allowing for the creation of two final hexamers, expanding the conformations available from previous studies. These tetramers are then joined to the final, separately optimized dimer, creating two hexamers which are subsequently subjected to iterative optimization again (Figure 9).

These hexamers are named according to their reconstruction strategy: geometries made with the N-terminal tetramer and C-terminal dimer are labeled as 4+2 (e.g., EAEE+EA), and those made with the N-terminal dimer and C-terminal tetramer are labelled as 2+4 (e.g., EA+EEEA). The final hexamers are visualized and analyzed for hydrogen bonding in Jmol [49] based on the Baker–Hubbard definition, where the distance between the hydrogen and acceptor heavy atom is less than or equal to 2.5 Å and the hydrogen bond angle is greater than or equal to 120° [50,51,52], criteria commonly employed to identify geometrically favorable hydrogen bonds while excluding weak or non-directional contacts.

### 3.2. Vibrational Frequency Analysis and Experimental Raman Spectra

Following each geometry optimization, harmonic vibrational frequency calculations are performed within Gaussian16 to obtain Raman intensities and frequencies. A scaling factor of 0.97 is applied to account for anharmonicity of the fundamental vibrational frequencies, and the spectra are plotted using a Lorentzian line function [53].

For experimental studies, chemically synthesized E-hooks, both full-length and hexamers, have been obtained from GenScript (lyophilized, purity ≥ 95%, Nanjing, China). Experimental Raman spectra are produced using a Horiba LabRAM HR Evolution Spectroscopy system (manufactured by HORIBA France SAS in Palaiseau, France) with the E-hooks in a solid state using an 1800 grooves/mm grating, 20 mW 532 nm laser excitation and CCD camera detection. Autocalibration is conducted using a silicon chip with the acquisition time and accumulations set to 3 s and 30, respectively. These experimental spectra are baseline-corrected using LabSpec 6 (version 6.5.1.24 32-bit) software, normalized, and vertically offset for direct comparison, allowing for experimental corroboration of the quantum-chemical results.

### 3.3. Raman Spectra Analysis

Comparison of computed and experimental Raman spectra is frequently performed through qualitative visual inspection in which corresponding peaks are identified manually and the overall agreement is assessed by the investigator [34,35,54,55,56]. While this approach can be effective for simple spectra, it becomes increasingly subjective for complex biomolecular systems containing numerous overlapping bands, broadened experimental features, and systematic frequency shifts arising from harmonic quantum-chemical approximations. Consequently, different investigators may emphasize certain spectral regions or assign peak correspondences differently, reducing reproducibility and making quantitative comparisons between computational methods difficult. The motivation for this present methodology, therefore, is to replace qualitative visual assessment with a systematic, reproducible, and objective comparison framework. Due to the mixed and shifted nature of these simulated spectra, this approach is used less as a means to exhaustively assign vibrational modes but more as a means to determine which buildup strategy most accurately reproduces experimentally observed vibrational frequency positions and peak distributions.

Raman spectra are compared using a region-specific dynamic time warping approach. Experimental and simulated spectra are normalized within the fingerprint region (200–2000 cm^−1^), and the simulated spectrum is interpolated onto the experimental wavenumber axis. Both spectra are smoothed using a Gaussian filter. DTW is then applied to the simulated spectrum with its experimental counterpart as the reference, allowing the simulated spectrum to stretch and warp to maximize overlap between the two spectral shapes while minimizing the cost of warping to achieve the overlap. By warping the spectrum, inherent shifting from the quantum-chemical simulation and its isolated conditions can be accounted for, allowing for assignment of local vibrational peaks in the warped form and recapitulation of this assignment in the non-warped spectra. The DTW spectra are used to identify candidate experimental peak positions corresponding to each simulated peak. Peaks are detected using the SciPy Python (version 1.17.1) library’s find_peaks function [57], and simulated peaks are assigned to experimental peaks within a frequency tolerance of 40 cm^−1^.

Peak assignments are scored using a weighted function containing a DTW-derived wavenumber distance term, a scaled intensity-difference term, and an occupancy penalty to discourage excessive assignment of multiple simulated peaks to the same experimental peak. The assignment for each simulated peak is selected as the candidate with the lowest matching score. This initial assignment establishes the correspondence between simulated and experimental peaks, while leaving unmatched peaks unassigned. A second calculation of the peaks’ score is then conducted, allowing the occupancy penalty to be applied evenly throughout the spectrum and distributing simulated peaks if many are assigned to a single experimental peak, if other experimental matches are available and appropriate. Agreement is assessed using weighted error functions incorporating peak position root-mean-square difference (RMSD), which is normalized by the approximate maximal shift determined by adding the DTW window in wavenumbers and frequency tolerance (in this study, approximately 65 cm^−1^), Pearson’s correlation error, and peak matching error. Three different weighting schemes have been used to separately emphasize positional agreement (Equation (1)), spectral-shape agreement (Equation (2)), and a balanced agreement between peak position and spectral shape (Equation (3)). These weights provide percentage windows into the different aspects of the scoring methodology, allowing for the quantitative differentiation of the multiple priorities that can be applied to an analytical spectral comparison method without negating and losing the resolution of any facet completely.

(1) $$S_{position}=0.6\left(RMSD\right)+0.3\left(1-Pearson\right)+0.1\left(1-Match\;Fraction\right)$$

(2) $$S_{shape}=0.4\left(RMSD\right)+0.4\left(1-Pearson\right)+0.2\left(1-Match\;Fraction\right)$$

(3) $$S_{balanced}=0.3\left(RMSD\right)+0.6\left(1-Pearson\right)+0.1\left(1-Match\;Fraction\right)$$

Existing Raman-matching approaches commonly rely upon point similarity scoring [58,59,60], peak-shift corrections [59,61], spectral segmentation [62], or unconstrained profile warping [63]. Although these methods are effective for spectral library searching and inter-instrument standardization, they are not designed for the comparison of simulated quantum-chemical spectra with experimental peptide spectra. Pointwise metrics are sensitive to frequency displacements [58,60], whereas unrestricted warping can produce favorable similarity scores through excessive or chemically ambiguous alignment [63]. The present methodology, therefore, combines constrained DTW with explicit peak detection, reduced redundant peak assignments, peak-position error, and matched-feature coverage. This framework tolerates expected calculated-to-experimental frequency shifts while retaining the magnitude of those shifts and identifying unmatched bands. It consequently provides both whole-spectrum similarity and mode-level interpretability, which are both necessary for evaluating basis-set and conformational effects in tubulin E-hook Raman spectra.

### 3.4. Structural Analysis

Backbone dihedral angles φ and ψ are calculated using the atomic coordinates from the optimized structures. Ramachandran plots are generated to visualize conformational distributions with unique marker shapes for each isotype and unique marker colors for each residue. These plots allow for identification of conformational trends both within and across isotypes.

The radius of gyration (R_g_) is the root-mean-square distance of the peptide’s atoms from its center of mass and is calculated for each peptide using atomic coordinates from the electronically optimized structures. The radius of gyration is defined as

(4) $$R_{g}=\sqrt{\frac{\sum_{i=1}^{N}{\left(r_{i}-r_{cm}\right)}^{2}}{N}}$$

where *r_i_* represents the position of atom *i* and *r_cm_* is the peptide’s center of mass. R_g_ provides a quantitative measure for peptide compactness [64].

## 4. Conclusions

The results herein demonstrate sequence-dependent differences in the optimized structures and vibrational characteristics of β-tubulin E-hook hexamers. Raman spectroscopy appears to provide an effective experimental framework for evaluating and differentiating computationally reconstructed β-tubulin E-hook peptides. By comparing experimentally obtained Raman spectra against quantum-chemically simulated spectra, structural models have been assessed according to their ability to reproduce observed vibrational frequencies and overall spectral behavior.

Rather than supporting a single universally preferred fragmentation strategy, the present results indicate that local amino acid composition determines how structural interactions and vibrational coupling are preserved during peptide reconstruction. βI, βIII, and βIV favor 4+2 fragmentation strategies, while βII and βVI favor 2+4 fragmentation strategies. Ramachandran analysis indicates that experimentally favored structures generally occupy more restricted distributions of backbone dihedral angles, while hydrogen bonding analysis reveals substantial sequence-dependent differences in local stabilization behavior driven primarily by polar and charged residues.

The level of the quantum-chemical theory employed also influences the predicted peptide geometry, although the magnitude of this effect is sequence- and reconstruction-dependent. Across the basis sets examined, changes in structural compactness are minimal for some E-hook structures, such as EA+QGPK (4.08–4.21 Å), but considerably larger for others, such as PEDK+GH (5.06–5.63 Å). The pronounced differences in compactness observed for the 2+4 reconstructions for the positively charged isotypes suggest that electrostatic interactions between acidic and basic residues influence E-hook conformational organization. Whether analogous interactions contribute to the conformational behavior of full-length E-hooks within a dynamic, solvated microtubule environment remains to be established.

These sequence-dependent structural differences may provide hypotheses for future investigation of E-hook interactions with positively charged regions of motor proteins and other microtubule-associated proteins. In particular, differences in charge distribution and conformational accessibility could potentially alter the presentation of E-hook residues to interacting proteins. However, such effects cannot be established from the static hexamer structures examined here and will require conformational sampling and explicit investigation of E-hook–protein interactions.

Other recent studies have shown that α-tubulin E-hooks are largely sequestered against the microtubule through interactions with basic residues in the tubulin core and become exposed only in the presence of specific MAPs [21]. In contrast, β-tubulin E-hooks are expected to access the surrounding cytoplasmic environment to a greater extent, thereby increasing their opportunity to interact with MAPs, motor proteins, and other binding partners. Understanding how the conformations of β-tubulin E-hooks vary with isotype-specific compositions therefore provides insight into the molecular basis of the tubulin code and how subtle sequence differences may give rise to distinct functional behaviors.

## Figures and Tables

**Figure 1:**
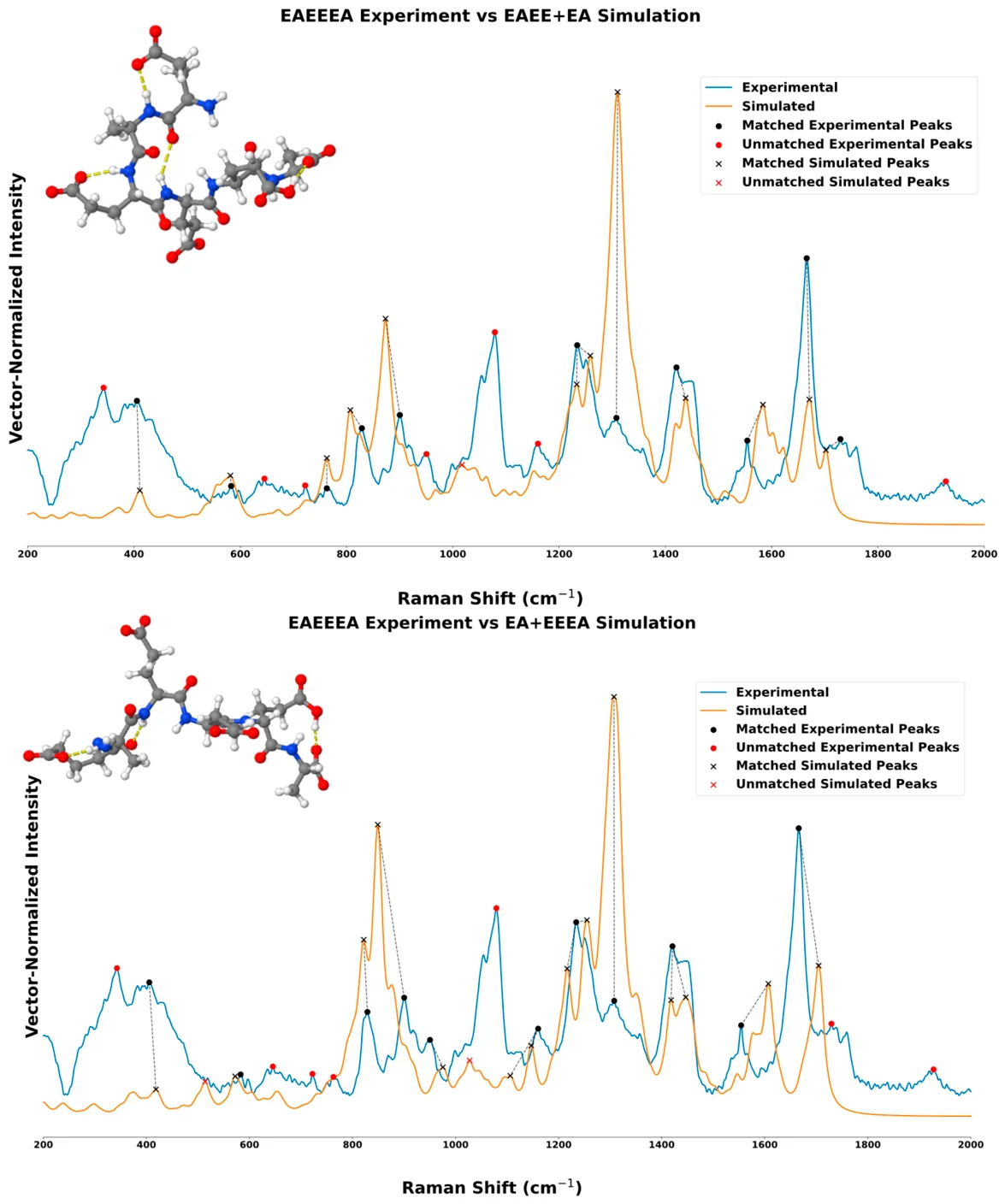
Raman spectral comparison of βI-tubulin E-hook hexamer (EAEEEA) experimental spectra with simulated spectra generated from alternative peptide construction strategies. The EAEE+EA (**top**) geometry demonstrates improved agreement with experimentally observed vibrational frequencies relative to the EA+EEEA (**bottom**) reconstruction, particularly through reduced peak-position deviations.

**Figure 2:**
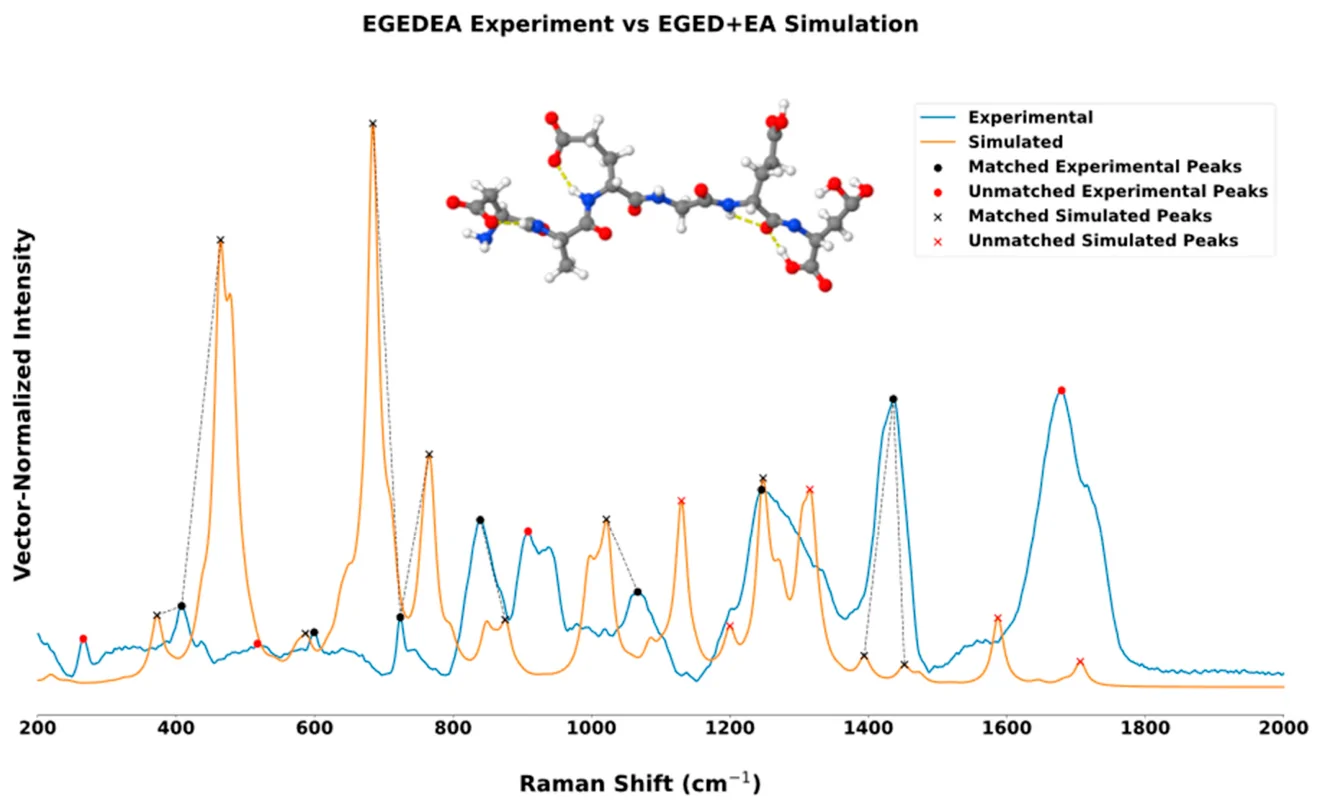

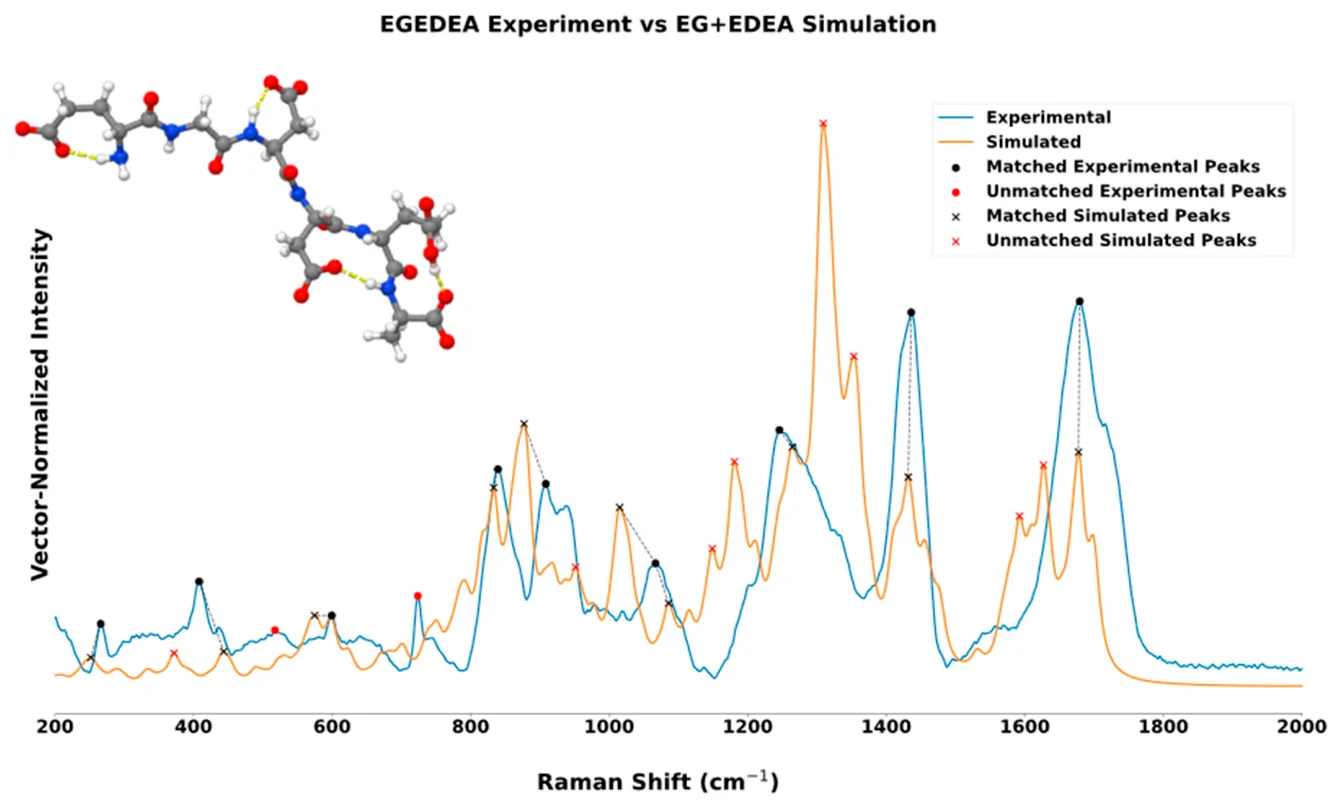
Raman spectral comparison of βII-tubulin E-hook hexamer (EGEDEA) experimental spectra with simulated spectra generated from alternative peptide construction strategies. The EA+EDEA (**bottom**) geometry demonstrates improved agreement with experimentally observed vibrational frequencies relative to the EGED+EA (**top**) reconstruction, particularly through reduced peak-position deviations and increased signal intensity similarity.

**Figure 3:**
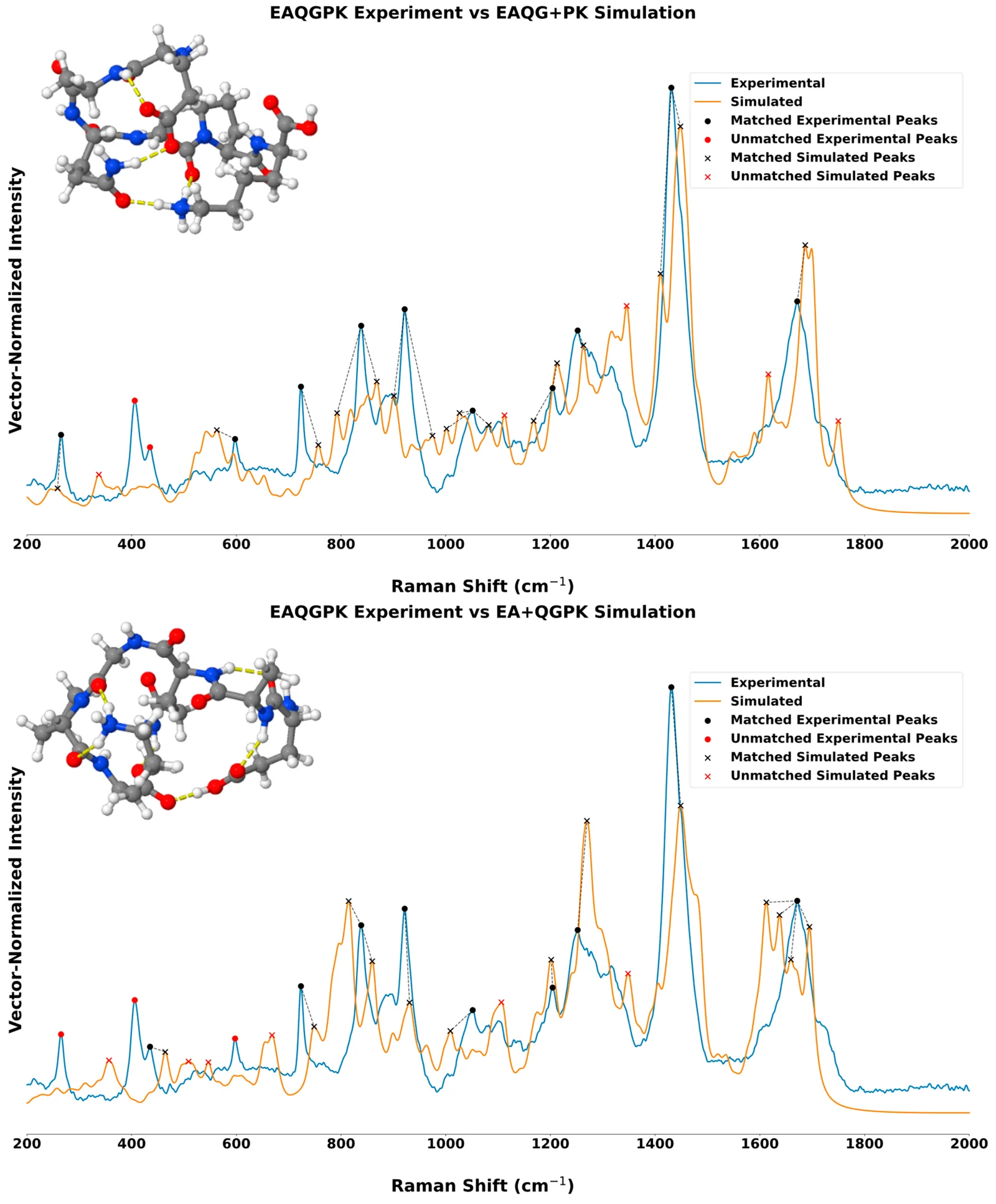
Raman spectral comparison of βIII-tubulin E-hook hexamer (EAQGPK) experimental spectra with simulated spectra generated from alternative peptide construction strategies. The EA+QGPK (**bottom**) geometry demonstrates improved agreement with experimentally observed vibrational frequencies relative to the EAQG+PK (**top**) reconstruction, particularly through reduced peak-position deviations.

**Figure 4:**
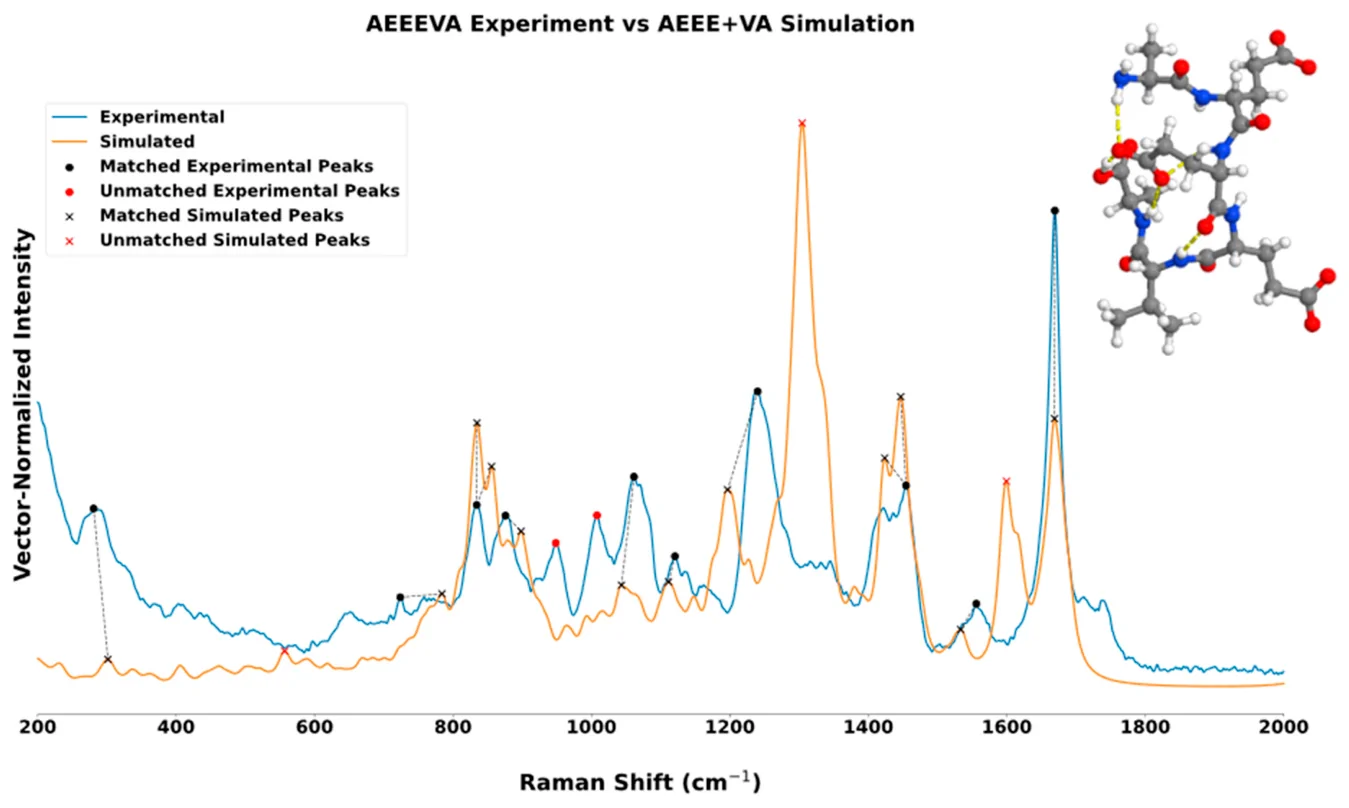

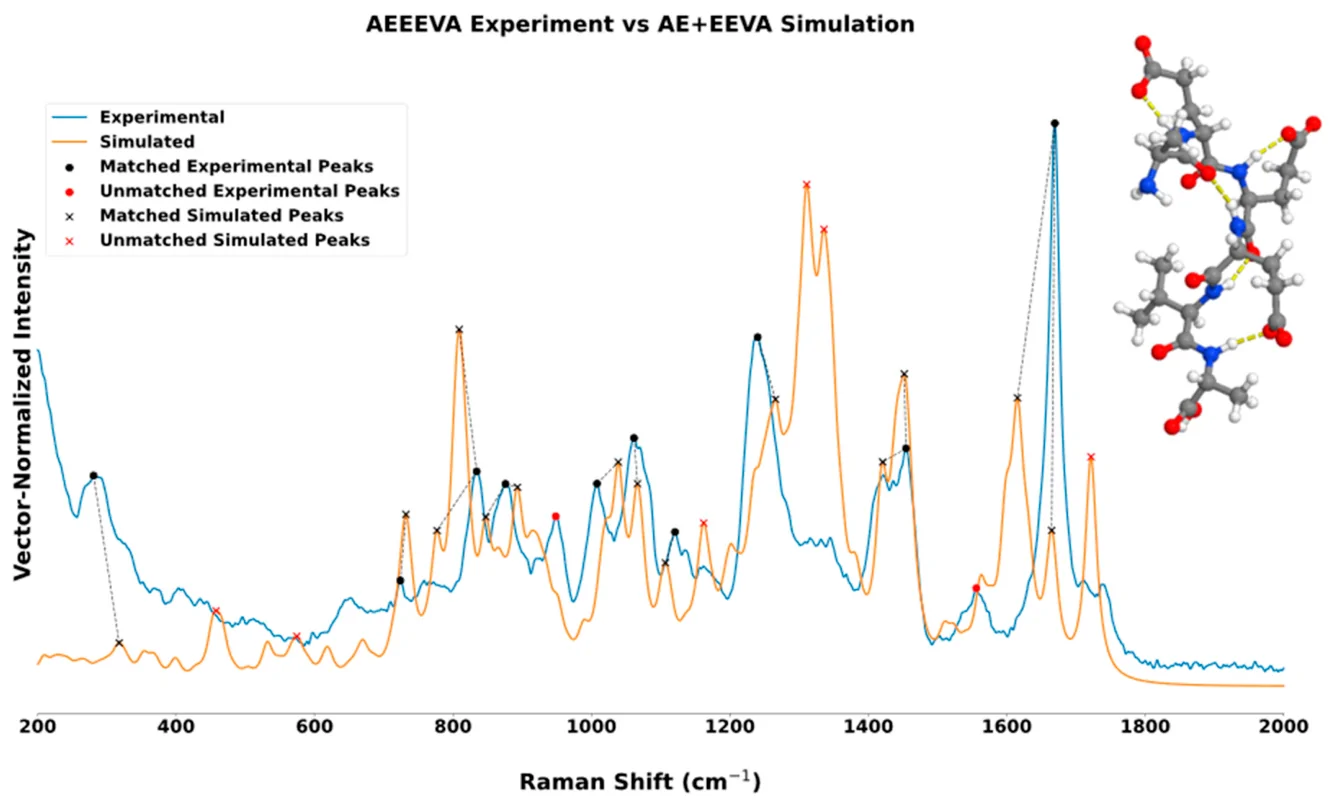
Raman spectral comparison of βIV-tubulin E-hook hexamer (AEEEVA) experimental spectra and its two simulated geometries. AEEE+VA reconstruction (**top**) shows slightly improved peak shift RMSD and Pearson coefficient compared to the AE+EEVA geometry (**bottom**).

**Figure 5:**
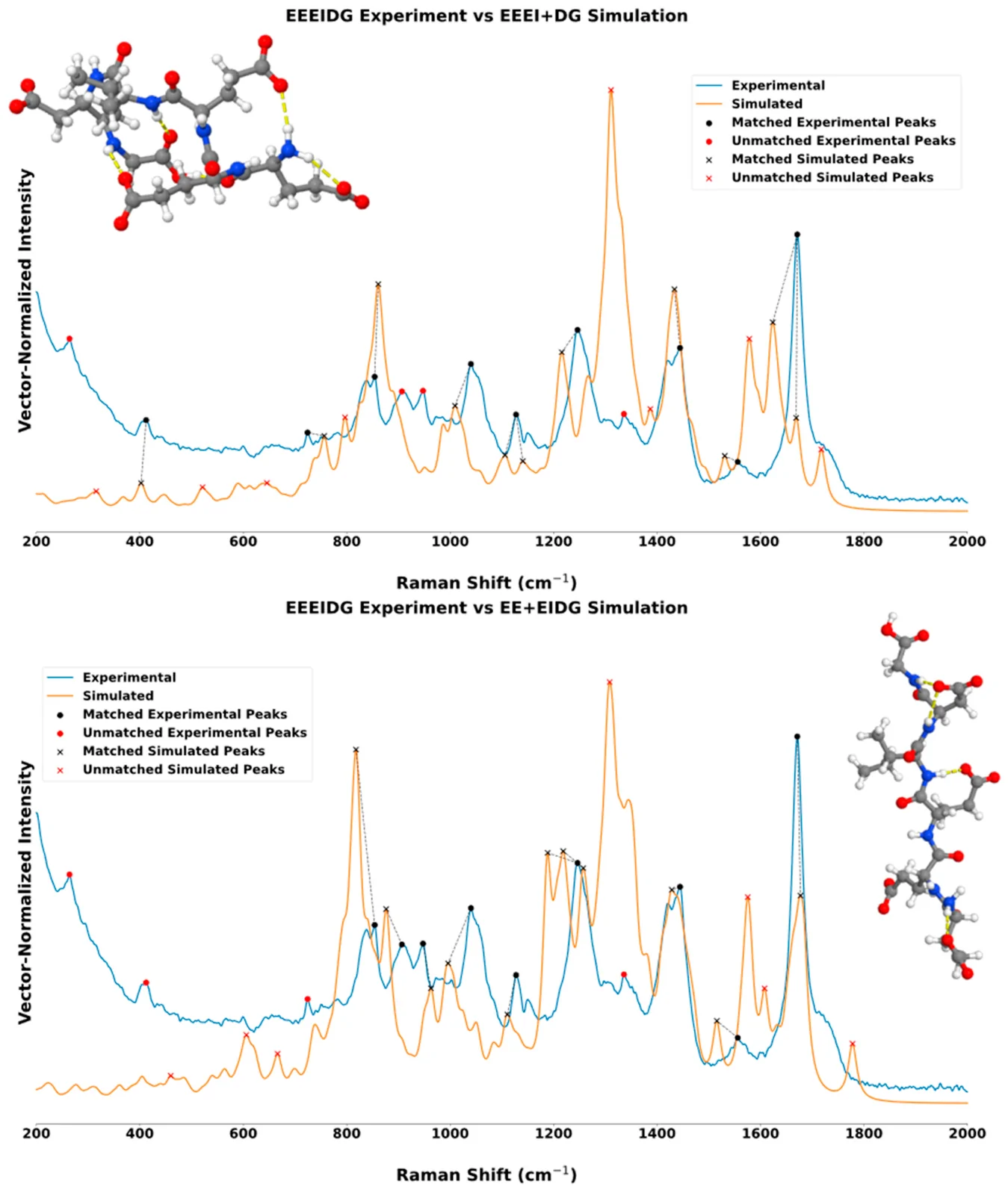
Raman spectral comparison of βV-tubulin E-hook hexamer (EEEIDG). Reconstruction using the EEEI+DG fragmentation strategy (**top**) substantially improves peak-position agreement despite reduced intensity correlation compared to EE+EIDG (**bottom**), indicating improved recovery of experimentally observed vibrational frequencies independent of exact intensity reproduction.

**Figure 6:**
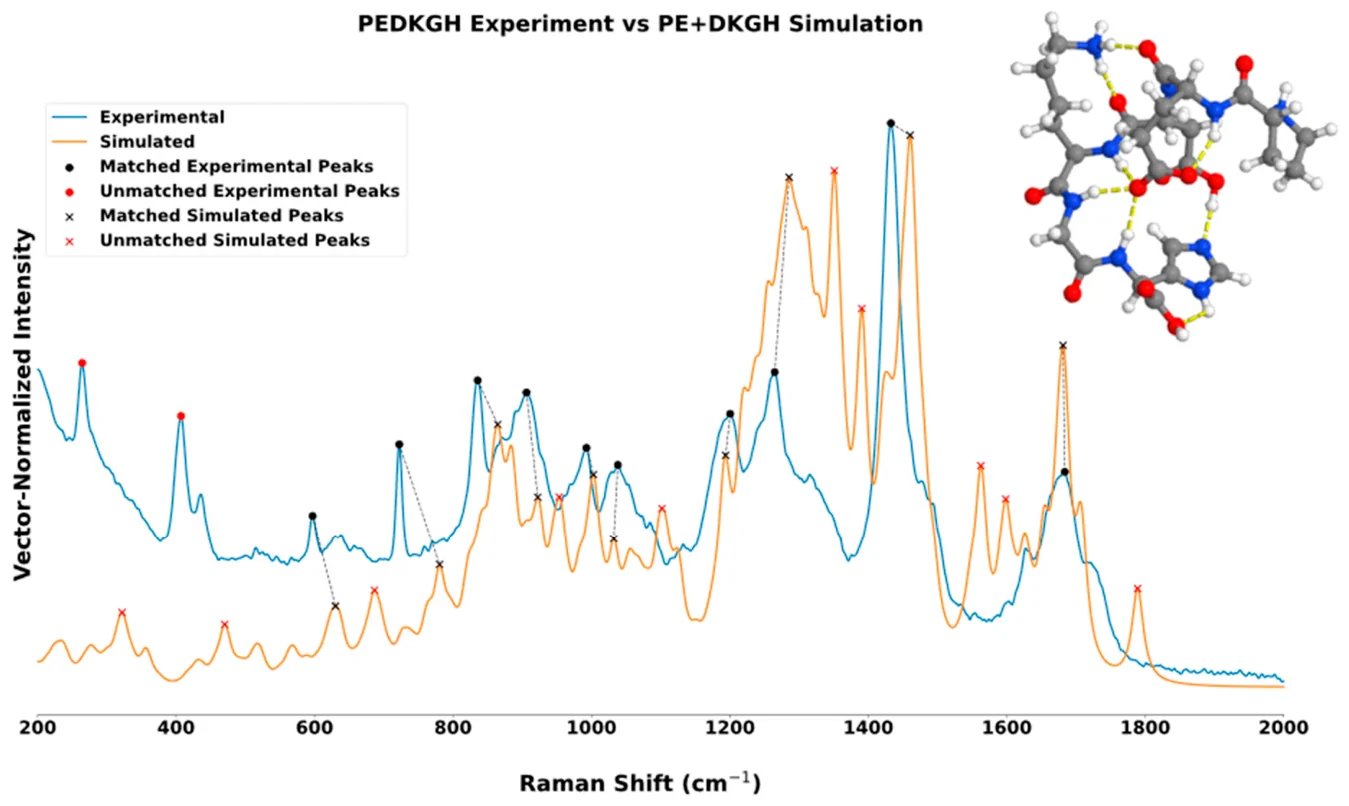

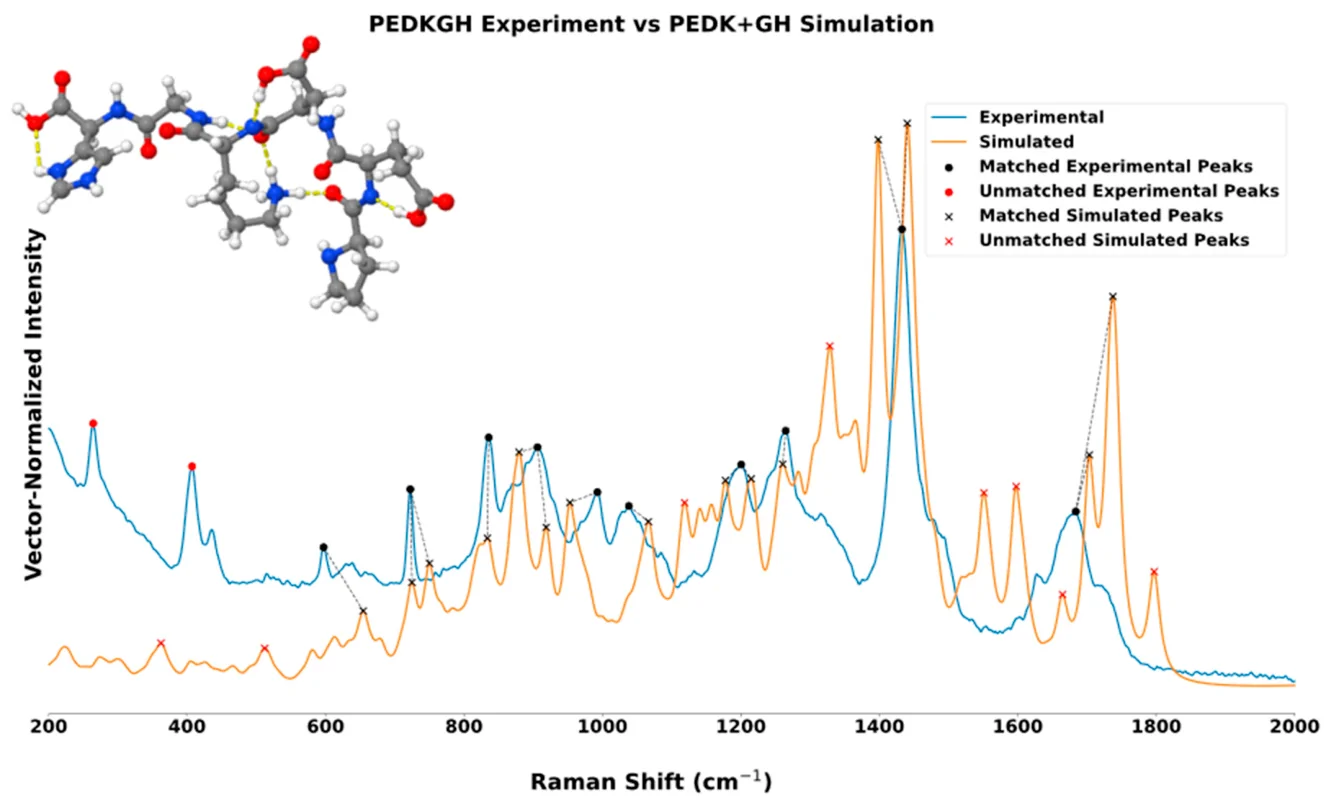
Experimental Raman spectra of the βVI-tubulin E-hook hexamer (PEDKGH) overlaid with simulated spectra. The PE+DKGH reconstruction (**bottom**) demonstrates improved overall agreement with experimentally observed vibrational frequencies relative to the PEDK+GH (**top**) geometry, although both structures exhibit comparatively similar spectral behavior.

**Figure 7:**
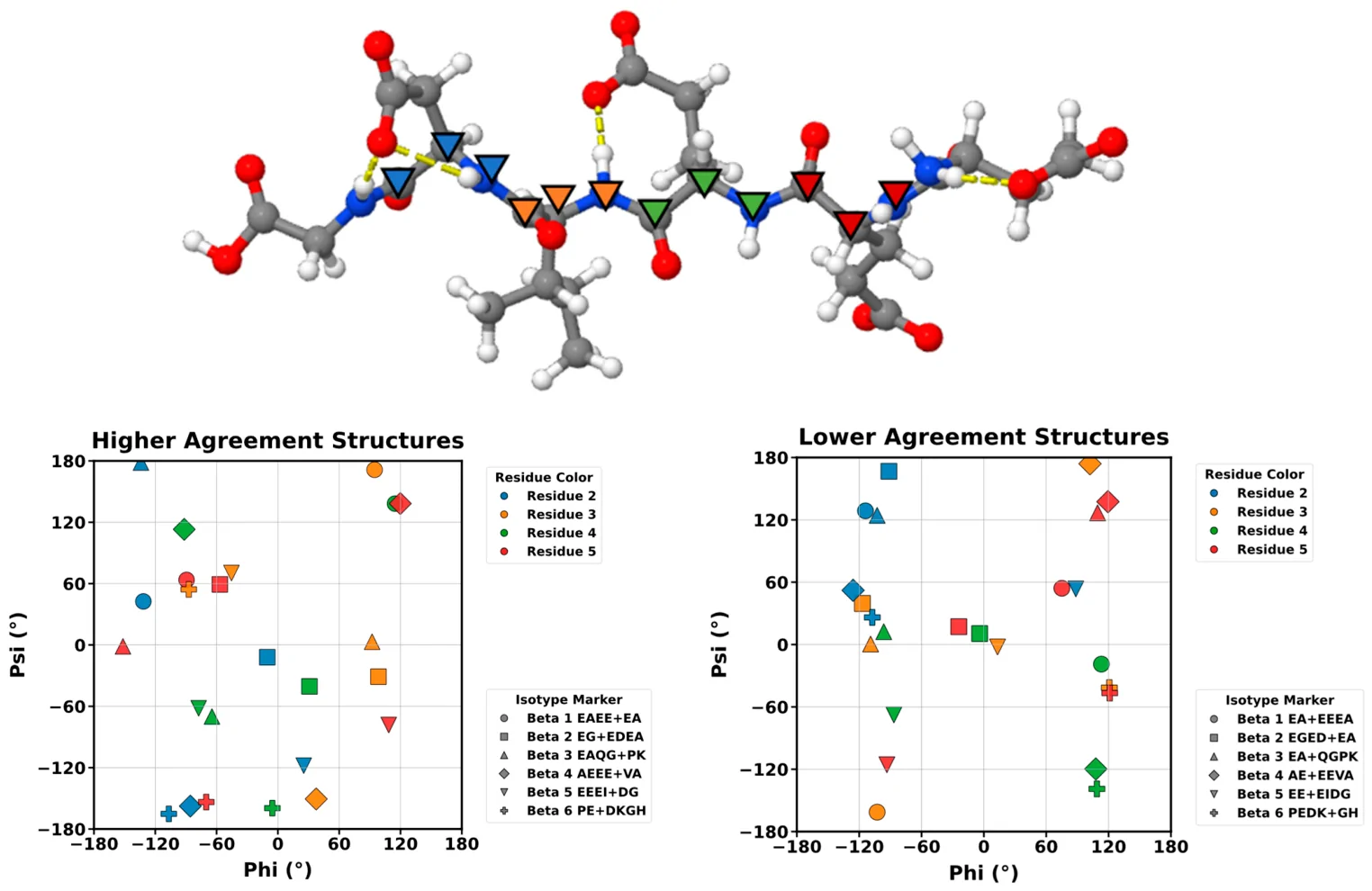
Ramachandran plots of higher-agreement simulated geometries (**left**) and lower-agreement simulated structures (**right**). Each structure is plotted by its isotype and residue number using unique marker shapes and marker colors, respectively. An example of the backbone labeling system is shown above, βV EE+EIDG (**top**).

**Figure 8:**
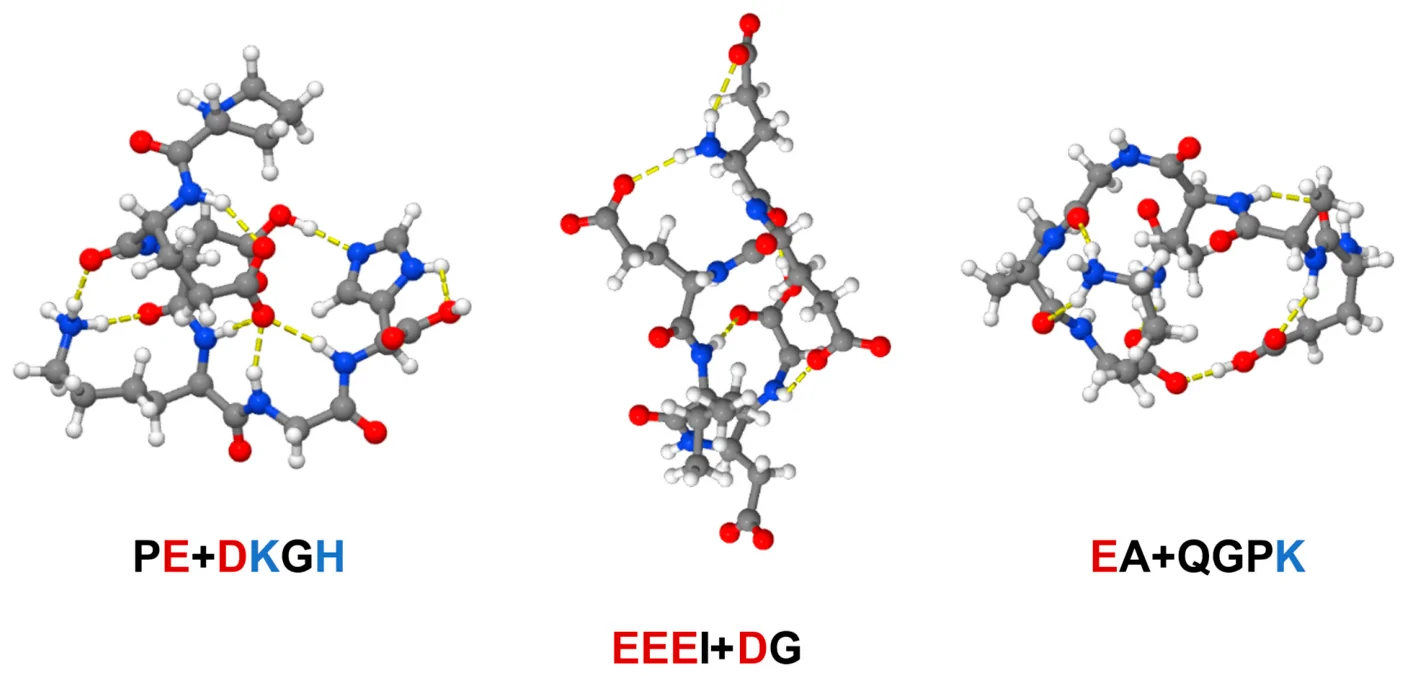
The three structures with the highest capacity for hydrogen bonding. Notice how the βVI (**left**) and βIII (**right**) structures obtain their high intramolecular interaction rate by adopting more closed conformers, while the βV (**middle**) structure has landed in a more sheet-like orientation.

**Figure 9:**
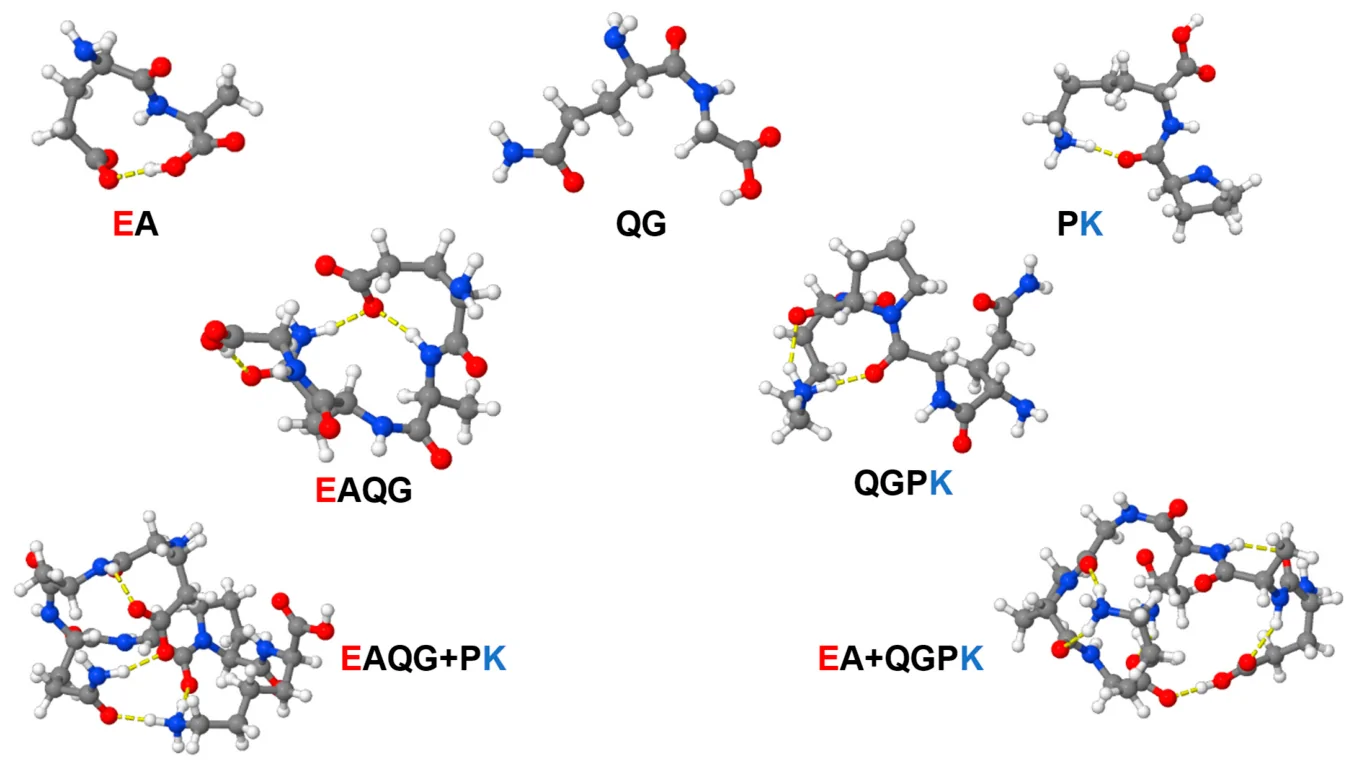
An example of the iterative optimization buildup method used to construct full hexamer E-hooks. Three dimers make two tetramers which, in turn, make the same hexamer but with two different conformations. Shown here is the process to build up to full hexamers of Beta III with hydrogen bonds shown in yellow.

**Table 1:** E-hooks and their sequences modeled and characterized in this study. Red denotes a negatively charged residue, and blue denotes a positively charged residue at pH 7. Histidine is partially protonated at this pH. Sequences were obtained from the NCBI protein database [37].

Beta I	EAEEEA
Beta II	EGEDEA
Beta III	EAQGPK
Beta IV	AEEEVA
Beta V	EEEIDG
Beta VI	PEDKGH

**Table 2:** Quantitative analysis performed on all simulated β-tubulin E-hook hexamers. The three metrics used for composite scoring and ranking are shown on the left: match fraction, peak shift RMSD, and the original Pearson coefficient. The Pearson coefficient of the warped spectra is also shown for reference. Each reconstruction with the lowest composite scores (highest agreement) across each isotype is bolded.

Simulated Peptide	Match Fraction	Peak Shift RMSD	Original Pearson	DTW Pearson	$S_{position}$	$S_{shape}$	$S_{balanced}$
β I							
EA+EEEA	0.65	0.46	0.22	0.40	0.55	0.57	0.64
**EAEE+EA**	0.65	0.27	0.30	0.51	**0.41**	**0.46**	**0.54**
β II							
**EG+EDEA**	0.82	0.39	0.55	0.77	**0.39**	**0.37**	**0.41**
EGED+EA	0.64	0.56	−0.10	0.06	0.70	0.74	0.86
β III							
EA+QGPK	0.75	0.43	0.71	0.92	0.37	0.34	0.33
**EAQG+PK**	0.83	0.47	0.80	0.96	**0.36**	**0.30**	**0.29**
β IV							
AE+EEVA	0.83	0.46	0.31	0.51	0.50	0.49	0.57
**AEEE+VA**	0.83	0.42	0.39	0.57	**0.45**	**0.44**	**0.51**
β V							
EE+EIDG	0.69	0.48	0.23	0.44	0.55	0.56	**0.64**
**EEEI+DG**	0.69	0.38	0.15	0.34	**0.51**	**0.55**	0.66
β VI							
**PE+DKGH**	0.83	0.41	0.40	0.60	**0.44**	**0.43**	**0.50**
PEDK+GH	0.83	0.44	0.34	0.57	0.48	0.48	0.55

**Table 3:** Average R_g_, in Å, of each β-tubulin E-hook isotype studied by basis set. βIII and βVI have lower R_g_ values due to their basic residues.

Basis Set	3-21G	6-31G	6-31+G(d,p)	6-311+G(2df,2pd)
Peptide				
β I				
EA+EEEA	5.43	5.11	5.20	5.22
EAEE+EA	5.94	6.07	6.07	6.07
β II				
EG+EDEA	6.66	6.67	6.70	6.69
EGED+EA	6.44	6.56	6.78	6.79
β III				
EA+QGPK	4.08	4.20	4.22	4.21
EAQG+PK	4.65	4.27	4.29	4.28
β IV				
AE+EEVA	5.01	5.11	5.26	5.25
AEEE+VA	4.70	4.78	4.88	4.87
β V				
EE+EIDG	6.22	6.44	6.53	6.51
EEEI+DG	4.79	4.99	5.04	5.04
β VI				
PE+DKGH	4.43	4.41	4.44	4.44
PEDK+GH	5.06	5.59	5.64	5.63

**Table 4:** Hydrogen bonds for each simulated geometry wherein the hydrogen’s distance to the heavy acceptor atom is less than or equal to 2.5 Å and their bond angle is greater than or equal to 120°. Intraresidue hydrogen bonds are hydrogen bonds wherein the acceptor and donor atoms are a part of the same residue. For example, a glutamic acid’s side-chain carboxylic acid group doubling back and interacting with its own backbone nitrogen or oxygen would be an intraresidue interaction.

Simulated Peptide	Total Hydrogen Bonds	Intraresidue Hydrogen Bonds	Interresidue Hydrogen Bonds
β I			
EA+EEEA	3	0	3
EAEE+EA	4	1	3
β II			
EG+EDEA	4	2	2
EGED+EA	3	1	2
β III			
EA+QGPK	6	0	6
EAQG+PK	4	0	4
β IV			
AE+EEVA	5	2	3
AEEE+VA	5	1	4
β V			
EE+EIDG	4	2	2
EEEI+DG	6	1	5
β VI			
PE+DKGH	7	1	6
PEDK+GH	3	0	3

**Table 5:** Hydrogen bonds formed by each amino acid throughout all simulation steps towards hexamer construction.

Amino Acid (AA)	Total Hydrogen Bonds	Hydrogen Bonds Per AA	Interresidue Hydrogen Bonds Per AA
Glu	54	1.8	1.5
Ala	14	1.4	1.2
Lys	11	2.8	2.8
Gly	9	1.1	1.1
Asp	8	1.3	1.2
Gln	4	2.0	2.0
His	2	1.0	1.0
Ile	2	1.0	1.0
Pro	2	0.5	0.5
Val	2	1.0	1.0

## Data Availability

The original contributions presented in this study are included in the article/Supplementary Materials. Further inquiries can be directed to the corresponding author(s).

## Supplementary Materials

The following supporting information can be downloaded at: https://www.mdpi.com/article/10.3390/molecules31193378/s1.
