# Supplementary material for: Tubulin E-Hook Hexamers Reveal Sequence-Dependent Compaction and Transient Secondary Structure Signatures
Source: Molecules. 2026 Sep 23;31(19):3378. doi: 10.3390/molecules31193378 (PMC13665854; doi:10.3390/molecules31193378)
Supplement: Supplementary file 1 [file molecules-31-03378-s001.zip › molecules-4527431-supplementary.pdf]

Supporting Information for "Tubulin E-hook Hexamers Reveal Sequence Dependent Compaction and Transient Secondary Structure Signatures"

Alexander C. Bromley[1], Nicholas A. Kruse[2], Connor R. Brower[2], Nathan I. Hammer[2], Ryan C. Fortenberry[2], Dana N. Reinemann[1,3]*

[1]Department of Biomedical Engineering, [2]Department of Chemistry and Biochemistry, [3]Department of Chemical Engineering, University of Mississippi, University, MS 38677 USA

*Corresponding Author: dnreinem@olemiss.edu

Table of Contents:

1. Table S1: EA 6-311+G(*2df,2pd*) Cartesian Coordinates
2. Table S2: EE 6-311+G(*2df,2pd*) Cartesian Coordinates
3. Table S3: EG 6-311+G(*2df,2pd*) Cartesian Coordinates
4. Table S4: ED 6-311+G(*2df,2pd*) Cartesian Coordinates
5. Table S5: QG 6-311+G(*2df,2pd*) Cartesian Coordinates
6. Table S6: PK 6-311+G(*2df,2pd*) Cartesian Coordinates
7. Table S7: AE 6-311+G(*2df,2pd*) Cartesian Coordinates
8. Table S8: VA 6-311+G(*2df,2pd*) Cartesian Coordinates
9. Table S9: EI 6-311+G(*2df,2pd*) Cartesian Coordinates
10. Table S10: DG 6-311+G(*2df,2pd*) Cartesian Coordinates
11. Table S11: PE 6-311+G(*2df,2pd*) Cartesian Coordinates
12. Table S12: DK 6-311+G(*2df,2pd*) Cartesian Coordinates
13. Table S13: GH 6-311+G(*2df,2pd*) Cartesian Coordinates
14. Table S14: EAEE 6-311+G(*2df,2pd*) Cartesian Coordinates
15. Table S15: EEEA 6-311+G(*2df,2pd*) Cartesian Coordinates
16. Table S16: EGED 6-311+G(*2df,2pd*) Cartesian Coordinates
17. Table S17: EDEA 6-311+G(*2df,2pd*) Cartesian Coordinates
18. Table S18: EAQG 6-311+G(*2df,2pd*) Cartesian Coordinates
19. Table S19: QGPK 6-311+G(*2df,2pd*) Cartesian Coordinates
20. Table S20: AEEE 6-311+G(*2df,2pd*) Cartesian Coordinates
21. Table S21: EEVA 6-311+G(*2df,2pd*) Cartesian Coordinates
22. Table S22: EEEI 6-311+G(*2df,2pd*) Cartesian Coordinates
23. Table S23: EIDG 6-311+G(*2df,2pd*) Cartesian Coordinates
24. Table S24: PEDK 6-311+G(*2df,2pd*) Cartesian Coordinates
25. Table S25: DKGH 6-311+G(*2df,2pd*) Cartesian Coordinates
26. Table S26: EAEE+EA 6-311+G(*2df,2pd*) Cartesian Coordinates
27. Table S27: EA+EEEA 6-311+G(*2df,2pd*) Cartesian Coordinates
28. Table S28: EGED+EA 6-311+G(*2df,2pd*) Cartesian Coordinates
29. Table S29: EG+EDEA 6-311+G(*2df,2pd*) Cartesian Coordinates
30. Table S30: EAQG+PK 6-311+G(*2df,2pd*) Cartesian Coordinates
31. Table S31: EA+QGPK 6-311+G(*2df,2pd*) Cartesian Coordinates
32. Table S32: AEEE+VA 6-311+G(*2df,2pd*) Cartesian Coordinates
33. Table S33: AE+EEVA 6-311+G(*2df,2pd*) Cartesian Coordinates
34. Table S34: EEEI+DG 6-311+G(*2df,2pd*) Cartesian Coordinates
35. Table S35: EE+EIDG 6-311+G(*2df,2pd*) Cartesian Coordinates
36. Table S36: PEDK+GH 6-311+G(*2df,2pd*) Cartesian Coordinates
37. Table S37: PE+DKGH 6-311+G(*2df,2pd*) Cartesian Coordinates
38. Figure S1: Structures of βI Buildup
39. Figure S2: Structures of βII Buildup
40. Figure S3: Structures of βIV Buildup
41. Figure S4: Structures of βV Buildup
42. Figure S5: Structures of βVI Buildup
43. Dynamic Time Warping Analysis Code

1. **Table S1:** Cartesian Coordinates for the low-energy geometry of the **EA** dipeptide calculated using the B3LYP method and the 6-311+G(*2df,2pd*) level of theory.

Standard Orientation					
Center Number	Atomic Number	Atomic Type	Coordinates (Angstroms)		
			X	Y	Z
1	6	0	-1.672265	1.534958	-0.184027
2	7	0	-2.106169	2.132056	1.081917
3	1	0	-2.842953	2.806596	0.925962
4	1	0	-1.313245	2.610039	1.497326
5	1	0	-1.713214	2.271015	-1.004739
6	6	0	-0.154902	1.254024	-0.065274
7	8	0	0.537720	1.969371	0.652477
8	7	0	0.356305	0.273272	-0.835901
9	6	0	1.768476	-0.096429	-0.836706
10	1	0	1.801957	-1.035156	-1.396550
11	6	0	2.354139	-0.458224	0.553591
12	8	0	3.477934	-0.116002	0.851205
13	8	0	1.638440	-1.225460	1.351496
14	1	0	0.736821	-1.559993	1.035473
15	6	0	2.634387	0.937577	-1.554595
16	1	0	2.252192	1.094486	-2.564470
17	1	0	3.665522	0.595606	-1.610094
18	1	0	2.618778	1.882392	-1.015958
19	1	0	-0.269425	-0.396477	-1.276595
20	6	0	-2.615709	0.387207	-0.598425
21	6	0	-2.598853	-0.879151	0.270007
22	1	0	-3.522313	-1.435250	0.084665
23	1	0	-2.569293	-0.622738	1.327185
24	6	0	-1.423575	-1.818622	-0.079764
25	8	0	-1.239370	-2.056253	-1.297697
26	8	0	-0.702653	-2.213611	0.876006
27	1	0	-2.421476	0.099347	-1.633694
28	1	0	-3.621063	0.820427	-0.587375

2. **Table S2:** Cartesian Coordinates for the low-energy geometry of the **EE** dipeptide calculated using the B3LYP method and the 6-311+G(*2df,2pd*) level of theory.

Center Number	Atomic Number	Atomic Type	Coordinates (Angstroms)		
			X	Y	Z
1	6	0	2.165583	0.532504	0.072056
2	6	0	2.751942	-0.522109	-0.886738
3	6	0	4.267913	-0.433359	-1.116486
4	6	0	5.253053	-0.885267	0.008081
5	8	0	6.462177	-0.792742	-0.292660
6	8	0	4.759254	-1.304437	1.089960
7	1	0	4.524627	-1.038649	-1.990897
8	1	0	4.541103	0.591114	-1.388275
9	1	0	2.489926	-1.511834	-0.504330
10	1	0	2.250570	-0.401618	-1.849429
11	7	0	2.348349	0.125338	1.469772
12	1	0	2.479767	0.936191	2.061695
13	1	0	3.198723	-0.453499	1.528517
14	6	0	0.685814	0.805700	-0.260402
15	7	0	-0.191248	0.347452	0.655357
16	6	0	-1.635236	0.566321	0.642993
17	6	0	-2.453884	-0.537339	-0.044753
18	6	0	-3.940308	-0.500848	0.315752
19	6	0	-4.775659	-1.768570	-0.077931
20	8	0	-6.017986	-1.588810	-0.084350
21	8	0	-4.136325	-2.819230	-0.308202
22	1	0	-4.047457	-0.404257	1.404388
23	1	0	-4.436132	0.368359	-0.115312
24	1	0	-2.050615	-1.499878	0.270613
25	1	0	-2.312912	-0.484418	-1.124362
26	6	0	-1.914243	2.004932	0.181482
27	8	0	-2.657860	2.101348	-0.936089
28	1	0	-2.738368	3.046926	-1.119315
29	8	0	-1.565631	2.984974	0.798185
30	1	0	-1.931150	0.584706	1.696041
31	1	0	0.247942	-0.096369	1.452827
32	8	0	0.355878	1.418268	-1.272812
33	1	0	2.658345	1.486933	-0.154655

3. **Table S3:** Cartesian Coordinates for the low-energy geometry of the **EG** dipeptide calculated using the B3LYP method and the 6-311+G(*2df,2pd*) level of theory.

**Standard Orientation**					
Center Number	Atomic Number	Atomic Type	Coordinates (Angstroms)		
			X	Y	Z
1	6	0	-1.383917	-1.357391	0.152249
2	7	0	-1.383350	-1.222938	1.617498
3	1	0	-0.605629	-1.733533	2.018541
4	1	0	-1.264296	-0.240351	1.847312
5	1	0	-1.632567	-2.393933	-0.082424
6	6	0	-2.478641	-0.461506	-0.454030
7	6	0	-2.524446	1.052728	-0.072236
8	8	0	-3.568351	1.643532	-0.373321
9	8	0	-1.499116	1.545854	0.501414
10	1	0	-2.428855	-0.515192	-1.545098
11	1	0	-3.447260	-0.876510	-0.172819
12	6	0	0.011808	-1.174933	-0.477961
13	8	0	0.613837	-2.124139	-0.984811
14	7	0	0.518138	0.075308	-0.401086
15	1	0	-0.130882	0.799591	-0.011882
16	6	0	1.798920	0.403170	-0.944027
17	1	0	2.105014	-0.389283	-1.632420
18	1	0	1.759932	1.334850	-1.511438
19	6	0	2.947286	0.560536	0.037984
20	8	0	4.020060	1.032757	-0.257588
21	8	0	2.682031	0.109521	1.280104
22	1	0	3.487291	0.251950	1.797090

4. **Table S4:** Cartesian Coordinates for the low-energy geometry of the **ED** dipeptide calculated using the B3LYP method and the 6-311+G(*2df,2pd*) level of theory.

**Standard Orientation**					
Center Number	Atomic Number	Atomic Type	Coordinates (Angstroms)		
			X	Y	Z
1	6	0	2.204382	0.529791	-0.320830
2	7	0	2.099997	1.200413	0.984306
3	1	0	1.837159	2.170957	0.851618
4	1	0	3.055537	1.187396	1.355390
5	1	0	2.693253	1.161268	-1.073231
6	6	0	3.046252	-0.749873	-0.179322
7	6	0	4.536318	-0.548845	0.256061
8	8	0	4.803291	0.480709	0.937072
9	8	0	5.320238	-1.456699	-0.089625
10	1	0	2.577854	-1.395833	0.571148
11	1	0	3.035286	-1.293486	-1.121786
12	6	0	0.824474	0.212714	-0.923033
13	8	0	0.693943	-0.039924	-2.116868
14	7	0	-0.196581	0.237988	-0.031909
15	1	0	0.077668	0.445446	0.919703
16	6	0	-1.606879	0.010766	-0.347741
17	1	0	-1.665650	-0.145141	-1.420816
18	6	0	-2.352127	1.314309	-0.070036
19	8	0	-2.610273	2.175618	-0.874723
20	8	0	-2.520122	1.530139	1.268001
21	1	0	-2.985086	2.373728	1.333977
22	6	0	-2.186577	-1.187972	0.405299
23	6	0	-3.725812	-1.349278	0.239517
24	8	0	-4.206774	-2.445464	0.597838
25	8	0	-4.321203	-0.341355	-0.220035
26	1	0	-1.684890	-2.096311	0.070423
27	1	0	-1.978034	-1.090703	1.474026

5. **Table S5:** Cartesian Coordinates for the low-energy geometry of the **QG** dipeptide calculated using the B3LYP method and the 6-311+G(*2df,2pd*) level of theory.

Standard Orientation					
Center Number	Atomic Number	Atomic Type	Coordinates (Angstroms)		
			X	Y	Z
1	6	0	-0.412220	1.377533	0.530833
2	7	0	-1.125142	2.585617	0.927396
3	1	0	-0.625965	3.063662	1.667744
4	1	0	-1.128985	3.227417	0.139967
5	1	0	-0.120439	0.850912	1.443413
6	6	0	-1.333426	0.453130	-0.282921
7	6	0	-2.538741	-0.046126	0.517684
8	6	0	-3.181000	-1.257521	-0.142401
9	7	0	-4.532281	-1.209516	-0.290433
10	1	0	-5.071507	-0.407888	-0.020352
11	1	0	-5.000875	-1.995950	-0.707872
12	8	0	-2.524559	-2.222126	-0.496290
13	1	0	-2.207499	-0.381952	1.504720
14	1	0	-3.250685	0.760078	0.684303
15	1	0	-0.780795	-0.413334	-0.645510
16	1	0	-1.671121	0.997037	-1.168666
17	6	0	0.845763	1.690056	-0.303627
18	8	0	0.926648	2.723153	-0.945597
19	7	0	1.847822	0.765676	-0.322060
20	1	0	2.654002	0.987437	-0.892786
21	6	0	1.912918	-0.491546	0.377671
22	1	0	1.857671	-0.364346	1.465059
23	1	0	1.102316	-1.170064	0.091969
24	6	0	3.236485	-1.178342	0.066892
25	8	0	4.101842	-0.690103	-0.598637
26	8	0	3.388098	-2.403745	0.611109
27	1	0	2.596176	-2.685993	1.084224

6. **Table S6:** Cartesian Coordinates for the low-energy geometry of the **PK** dipeptide calculated using the B3LYP method and the 6-311+G(*2df,2pd*) level of theory.

**Standard Orientation**					
Center Number	Atomic Number	Atomic Type	Coordinates (Angstroms)		
			X	Y	Z
1	6	0	2.062380	-1.114524	-0.475830
2	6	0	3.083226	-1.613153	0.582565
3	6	0	4.031437	-0.419337	0.757401
4	6	0	4.098730	0.168400	-0.650293
5	7	0	2.711470	0.017755	-1.148921
6	1	0	2.669068	-0.051343	-2.157354
7	1	0	4.812638	-0.392621	-1.264497
8	1	0	4.392595	1.218446	-0.663567
9	1	0	3.611846	0.311363	1.453701
10	1	0	5.010563	-0.712749	1.134720
11	1	0	3.625785	-2.468140	0.175346
12	1	0	2.602997	-1.937760	1.504508
13	1	0	1.805799	-1.925356	-1.164854
14	6	0	0.737287	-0.710569	0.181739
15	8	0	0.031591	-1.589975	0.724984
16	7	0	0.444452	0.582507	0.116979
17	1	0	1.140010	1.126393	-0.403436
18	6	0	-0.651814	1.310383	0.742019
19	6	0	-2.026304	0.614795	0.686583
20	1	0	-1.970579	-0.236517	1.362410
21	1	0	-2.752867	1.301358	1.123775
22	6	0	-2.489850	0.192211	-0.715417
23	1	0	-2.868464	1.070389	-1.242815
24	1	0	-1.643096	-0.165592	-1.308270
25	6	0	-3.590919	-0.884163	-0.703187
26	1	0	-4.282308	-0.718936	0.130826
27	6	0	-3.104626	-2.330523	-0.696138
28	1	0	-2.439681	-2.524913	-1.538223
29	1	0	-3.946153	-3.020485	-0.758610
30	7	0	-2.320376	-2.701167	0.541647
31	1	0	-2.865555	-2.529050	1.387727
32	1	0	-2.083863	-3.694792	0.532671
33	1	0	-1.389506	-2.163730	0.616540
34	1	0	-4.198142	-0.793710	-1.607390
35	1	0	-0.419598	1.468636	1.802301
36	6	0	-0.666243	2.705786	0.113788
37	8	0	0.109829	3.082762	-0.722580
38	8	0	-1.639047	3.467069	0.639715
39	1	0	-1.579108	4.350758	0.240218

7. **Table S7:** Cartesian Coordinates for the low-energy geometry of the **AE** dipeptide calculated using the B3LYP method and the 6-311+G(*2df,2pd*) level of theory.

Standard Orientation					
Center Number	Atomic Number	Atomic Type	Coordinates (Angstroms)		
			X	Y	Z
1	6	0	-2.466550	-1.643044	-0.197501
2	7	0	-2.604576	-2.528303	0.959797
3	1	0	-2.883349	-1.963758	1.757018
4	1	0	-1.702845	-2.923951	1.202161
5	1	0	-1.963039	-2.208586	-0.985418
6	6	0	-1.644623	-0.384510	0.138121
7	8	0	-1.863302	0.247797	1.162260
8	7	0	-0.722030	-0.019756	-0.782174
9	1	0	-0.489973	-0.691597	-1.494063
10	6	0	0.278871	1.031875	-0.571089
11	6	0	1.519873	0.542986	0.177171
12	1	0	1.239209	0.265090	1.194664
13	6	0	2.208115	-0.620354	-0.534225
14	1	0	1.626137	-1.541793	-0.469455
15	1	0	2.316531	-0.387512	-1.602746
16	6	0	3.661370	-0.938384	-0.023930
17	8	0	4.013347	-2.129712	-0.166304
18	8	0	4.303564	0.035195	0.425536
19	1	0	2.233110	1.361378	0.267123
20	6	0	-0.356860	2.286944	0.018286
21	8	0	0.105973	2.968352	0.890912
22	8	0	-1.482099	2.649817	-0.657185
23	1	0	-1.809083	3.445715	-0.216329
24	1	0	0.589432	1.338713	-1.576425
25	6	0	-3.850692	-1.221748	-0.695038
26	1	0	-4.371717	-0.666008	0.085635
27	1	0	-4.437691	-2.105185	-0.943988
28	1	0	-3.777379	-0.580266	-1.574050

8. **Table S8:** Cartesian Coordinates for the low-energy geometry of the **VA** dipeptide calculated using the B3LYP method and the 6-311+G(*2df,2pd*) level of theory.

Standard Orientation					
Center Number	Atomic Number	Atomic Type	Coordinates (Angstroms)		
			X	Y	Z
1	6	0	-1.816384	-0.399750	0.470839
2	7	0	-2.548873	-1.635168	0.757304
3	1	0	-2.059779	-2.194486	1.445514
4	1	0	-2.600984	-2.191436	-0.089963
5	1	0	-1.601488	0.085686	1.428246
6	6	0	-0.498822	-0.709402	-0.254786
7	8	0	-0.479198	-1.367635	-1.284518
8	7	0	0.635206	-0.232082	0.325715
9	1	0	0.570906	0.317763	1.165037
10	6	0	1.947143	-0.440684	-0.265042
11	6	0	2.591775	-1.766780	0.174695
12	1	0	2.733849	-1.791027	1.255610
13	1	0	3.557191	-1.897077	-0.311308
14	1	0	1.938136	-2.587320	-0.113434
15	6	0	2.899726	0.704018	0.030296
16	8	0	2.523106	1.454493	1.095098
17	1	0	3.201881	2.132908	1.224376
18	8	0	3.912879	0.909709	-0.579783
19	1	0	1.818620	-0.468432	-1.346435
20	6	0	-2.689075	0.558352	-0.371899
21	6	0	-3.971223	0.929240	0.377087
22	1	0	-4.623425	1.530296	-0.258290
23	1	0	-3.742779	1.521386	1.267622
24	1	0	-4.515613	0.042647	0.693301
25	6	0	-1.924208	1.816414	-0.794559
26	1	0	-1.583516	2.383012	0.076340
27	1	0	-2.570951	2.473837	-1.376399
28	1	0	-1.054789	1.585004	-1.409828
29	1	0	-2.959479	0.007005	-1.277683

9. **Table S9:** Cartesian Coordinates for the low-energy geometry of the **EI** dipeptide calculated using the B3LYP method and the 6-311+G(*2df,2pd*) level of theory.

Standard Orientation					
Center Number	Atomic Number	Atomic Type	Coordinates (Angstroms)		
			X	Y	Z
1	6	0	-1.435883	-1.114700	1.155853
2	7	0	-1.630989	-2.442053	1.743164
3	1	0	-2.480643	-2.457773	2.291201
4	1	0	-0.844736	-2.676799	2.336646
5	1	0	-1.530560	-0.311428	1.908334
6	6	0	-2.505659	-0.847254	0.085290
7	1	0	-2.347626	-1.531547	-0.752221
8	1	0	-3.470457	-1.115735	0.522277
9	6	0	-2.673690	0.597158	-0.397496
10	1	0	-2.315428	1.301640	0.361827
11	1	0	-2.121042	0.815506	-1.316724
12	6	0	-4.166645	1.030478	-0.685277
13	8	0	-5.050640	0.383886	-0.084210
14	8	0	-4.275354	2.004163	-1.459509
15	6	0	0.032182	-1.030148	0.724134
16	8	0	0.918746	-1.644711	1.309493
17	7	0	0.320733	-0.199494	-0.306384
18	1	0	-0.441123	0.271230	-0.769037
19	6	0	1.677502	0.040817	-0.762601
20	6	0	2.528086	0.992850	0.121464
21	6	0	1.678483	2.140410	0.690275
22	6	0	2.422874	3.039583	1.678759
23	1	0	1.744247	3.776985	2.109865
24	1	0	3.242563	3.585734	1.210628
25	1	0	2.840403	2.454014	2.501089
26	1	0	0.807125	1.723610	1.193172
27	1	0	1.288630	2.749002	-0.133117
28	6	0	3.718341	1.525838	-0.687172
29	1	0	4.304351	0.715585	-1.114149
30	1	0	3.372555	2.172333	-1.499674
31	1	0	4.386041	2.116031	-0.060177
32	1	0	2.908748	0.400983	0.956077
33	1	0	1.573232	0.503818	-1.749453
34	6	0	2.401000	-1.288650	-1.025612
35	8	0	1.624970	-2.140618	-1.729790
36	1	0	2.133094	-2.956760	-1.833093
37	8	0	3.540137	-1.548207	-0.736815

10. **Table S10:** Cartesian Coordinates for the low-energy geometry of the **DG** dipeptide calculated using the B3LYP method and the 6-311+G(*2df,2pd*) level of theory.

**Standard Orientation**					
Center Number	Atomic Number	Atomic Type	Coordinates (Angstroms)		
			X	Y	Z
1	6	0	1.156288	-0.127405	-0.605401
2	1	0	1.481796	-0.036260	-1.647906
3	7	0	1.022830	-1.523751	-0.167823
4	1	0	0.666650	-2.093177	-0.927242
5	1	0	1.989808	-1.824381	0.021085
6	6	0	-0.178814	0.609175	-0.520360
7	8	0	-0.422064	1.644784	-1.128073
8	7	0	-1.069319	0.028645	0.335090
9	1	0	-0.764098	-0.859323	0.717705
10	6	0	-2.346727	0.598083	0.605778
11	1	0	-2.491678	0.818991	1.668808
12	1	0	-2.402599	1.552298	0.075740
13	6	0	-3.518181	-0.252266	0.161245
14	8	0	-4.695615	0.344861	0.497098
15	1	0	-5.399077	-0.241476	0.186162
16	8	0	-3.479092	-1.314365	-0.397869
17	6	0	2.187337	0.603753	0.277894
18	6	0	3.639935	0.013167	0.267725
19	8	0	4.544557	0.838472	0.482986
20	8	0	3.742985	-1.231244	0.085049
21	1	0	1.841202	0.572410	1.316800
22	1	0	2.238218	1.649572	-0.016821

11. **Table S12:** Cartesian Coordinates for the low-energy geometry of the **PE** dipeptide calculated using the B3LYP method and the 6-311+G(*2df,2pd*) level of theory.

Standard Orientation					
Center Number	Atomic Number	Atomic Type	Coordinates (Angstroms)		
			X	Y	Z
1	6	0	-4.452961	-0.235661	0.055020
2	7	0	-3.413280	0.775920	-0.066789
3	1	0	-3.633044	1.560538	-0.664002
4	6	0	-2.223543	0.052609	-0.499462
5	1	0	-2.344208	-0.344262	-1.526177
6	6	0	-2.206867	-1.138787	0.471442
7	6	0	-3.712379	-1.420918	0.720527
8	1	0	-4.026977	-2.371094	0.288520
9	1	0	-3.926844	-1.464363	1.788095
10	1	0	-1.660484	-1.992271	0.078404
11	1	0	-1.715565	-0.839724	1.396115
12	6	0	-0.997507	0.960923	-0.563055
13	8	0	-1.124302	2.172121	-0.754366
14	7	0	0.186764	0.336640	-0.471762
15	1	0	0.298972	-0.708445	-0.384848
16	6	0	1.422415	1.051541	-0.669664
17	6	0	2.572092	0.080166	-1.022506
18	6	0	2.930517	-1.002414	0.011582
19	6	0	2.092567	-2.317314	-0.006979
20	8	0	0.873195	-2.214824	-0.350388
21	8	0	2.689893	-3.347945	0.334633
22	1	0	2.865344	-0.597866	1.026771
23	1	0	3.971481	-1.294444	-0.126306
24	1	0	3.449357	0.700887	-1.213838
25	1	0	2.304482	-0.397388	-1.967077
26	1	0	1.306568	1.758090	-1.494937
27	6	0	1.837423	1.938415	0.502071
28	8	0	1.242683	1.634215	1.673291
29	1	0	1.589531	2.262104	2.322399
30	8	0	2.653740	2.825098	0.412464
31	1	0	-4.854834	-0.554609	-0.922036
32	1	0	-5.290366	0.134252	0.649842

12. **Table S11:** Cartesian Coordinates for the low-energy geometry of the **DK** dipeptide calculated using the B3LYP method and the 6-311+G(*2df,2pd*) level of theory.

Standard Orientation					
Center Number	Atomic Number	Atomic Type	Coordinates (Angstroms)		
			X	Y	Z
1	6	0	0.530434	1.225753	-0.657763
2	7	0	-0.669302	0.421649	-0.463102
3	6	0	-0.756831	-0.674071	0.180057
4	6	0	-2.109766	-1.369940	0.329601
5	7	0	-1.916949	-2.810545	0.123206
6	1	0	-1.203148	-3.167250	0.744287
7	1	0	-2.777404	-3.306176	0.318010
8	1	0	-2.436954	-1.136530	1.355291
9	6	0	-3.197148	-0.897160	-0.650820
10	6	0	-3.899307	0.416371	-0.313267
11	8	0	-5.074183	0.469065	-0.050019
12	8	0	-3.135022	1.510385	-0.352701
13	1	0	-3.978753	-1.652966	-0.669998
14	1	0	-2.768990	-0.835504	-1.652414
15	8	0	0.180720	-1.368862	0.830463
16	1	0	-2.192709	1.228064	-0.513197
17	6	0	1.734615	0.441755	-1.280844
18	1	0	1.733466	0.635894	-2.353422
19	1	0	1.514339	-0.617266	-1.181649
20	6	0	3.126250	0.736386	-0.705239
21	1	0	3.531047	1.649802	-1.141920
22	6	0	4.126555	-0.409206	-0.898762
23	1	0	4.352358	-0.553661	-1.958101
24	6	0	3.666480	-1.754704	-0.323023
25	1	0	2.955477	-2.233094	-0.998172
26	7	0	2.982889	-1.592703	0.976877
27	1	0	3.576643	-1.098804	1.634598
28	1	0	2.797835	-2.501225	1.387179
29	1	0	1.118338	-1.060695	0.819590
30	1	0	4.529850	-2.424462	-0.257877
31	1	0	5.069534	-0.119635	-0.426755
32	1	0	3.052061	0.934882	0.363579
33	6	0	0.919392	2.047317	0.573624
34	8	0	1.426088	3.135523	0.507269
35	8	0	0.647075	1.441019	1.748328
36	1	0	0.898657	2.056678	2.452563
37	1	0	0.250430	2.001097	-1.368556

13. **Table S13:** Cartesian Coordinates for the low-energy geometry of the **GH** dipeptide calculated using the B3LYP method and the 6-311+G(*2df,2pd*) level of theory.

Standard Orientation					
Center Number	Atomic Number	Atomic Type	Coordinates (Angstroms)		
			X	Y	Z
1	6	0	3.353503	-0.090710	-0.402754
2	7	0	4.376717	0.780769	0.109195
3	1	0	4.146179	1.755717	-0.028663
4	1	0	4.526631	0.644091	1.099945
5	1	0	3.645352	-1.131465	-0.235350
6	1	0	3.283072	0.023948	-1.488797
7	6	0	1.950325	0.106252	0.177185
8	8	0	1.659979	0.965433	0.988214
9	7	0	0.981069	-0.750767	-0.299851
10	1	0	1.222264	-1.579688	-0.825686
11	6	0	-0.304679	-0.763583	0.344073
12	1	0	-0.181503	-0.761545	1.433924
13	6	0	-1.119074	0.452968	-0.006876
14	6	0	-1.143764	1.214964	-1.133137
15	7	0	-2.050701	2.230457	-0.908382
16	6	0	-2.573065	2.109406	0.307872
17	7	0	-2.020023	1.035210	0.864524
18	1	0	-2.229293	0.701018	1.794585
19	1	0	-3.302794	2.758271	0.758154
20	1	0	-2.280414	2.967507	-1.559083
21	1	0	-0.584543	1.131796	-2.045784
22	6	0	-1.038875	-2.062891	-0.017094
23	8	0	-0.577654	-2.923259	-0.706795
24	8	0	-2.247400	-2.097081	0.565283
25	1	0	-2.680017	-2.938502	0.349130

14. **Table S14:** Cartesian Coordinates for the low-energy geometry of the **EAEE** tetrapeptide calculated using the B3LYP method and the 6-311+G(*2df,2pd*) level of theory.

**Standard Orientation**					
Center Number	Atomic Number	Atomic Type	Coordinates (Angstroms)		
			X	Y	Z
1	6	0	2.669508	-2.764386	-0.806914
2	7	0	2.005286	-3.610446	0.195716
3	1	0	1.973600	-4.570776	-0.123867
4	1	0	1.048016	-3.291344	0.301356
5	1	0	2.330754	-3.021048	-1.824464
6	6	0	2.111601	-1.334718	-0.646360
7	8	0	0.880326	-1.204981	-0.692265
8	7	0	2.964714	-0.316524	-0.527176
9	6	0	2.516409	1.077826	-0.525236
10	1	0	3.356264	1.641180	-0.113272
11	6	0	1.372292	1.290655	0.498970
12	8	0	1.487638	0.864683	1.640222
13	7	0	0.284512	1.977058	0.066506
14	6	0	-0.810744	2.304497	0.963197
15	6	0	-1.521112	3.592062	0.507018
16	6	0	-0.659233	4.863863	0.499346
17	6	0	0.102449	5.201382	-0.817741
18	8	0	0.447024	6.395177	-0.952518
19	8	0	0.310537	4.246816	-1.617622
20	1	0	-1.281506	5.731565	0.728280
21	1	0	0.090579	4.818495	1.296937
22	1	0	-1.929182	3.430759	-0.493688
23	1	0	-2.367891	3.720636	1.180343
24	6	0	-1.867192	1.188990	1.138325
25	7	0	-1.712503	0.056762	0.426633
26	6	0	-2.629458	-1.084164	0.504818
27	6	0	-3.779118	-1.050316	-0.522017
28	6	0	-4.432389	-2.420075	-0.731369
29	6	0	-5.638821	-2.492241	-1.727317
30	8	0	-6.144161	-3.639360	-1.842046
31	8	0	-5.982025	-1.434805	-2.301511
32	1	0	-3.685069	-3.134858	-1.093187
33	1	0	-4.793963	-2.824362	0.216326
34	1	0	-3.360715	-0.703488	-1.467282
35	1	0	-4.523855	-0.312650	-0.229601
36	6	0	-3.063608	-1.310401	1.961473
37	8	0	-4.395926	-1.208427	2.173856
38	1	0	-4.512451	-1.360326	3.120722
39	8	0	-2.315040	-1.642218	2.848623
40	1	0	-2.004478	-1.953026	0.286971
41	1	0	-0.839933	-0.096779	-0.065968
42	8	0	-2.809944	1.367917	1.908279
43	1	0	-0.408226	2.452256	1.969146
44	1	0	0.342898	2.582484	-0.760529
45	6	0	2.253952	1.565485	-1.953124
46	1	0	3.157629	1.414730	-2.545892
47	1	0	1.990734	2.622650	-1.984255
48	1	0	1.444269	0.996528	-2.409393

49	1	0	3.990316	-0.468707	-0.445811
50	6	0	4.188374	-2.970999	-0.798479
51	6	0	4.951804	-2.686566	0.513816
52	1	0	5.373165	-3.602103	0.927236
53	1	0	4.259343	-2.294831	1.262412
54	6	0	6.086572	-1.644559	0.333565
55	8	0	5.740661	-0.583278	-0.266090
56	8	0	7.215789	-1.925442	0.785554
57	1	0	4.611677	-2.358777	-1.597177
58	1	0	4.357251	-4.012828	-1.094781

15. **Table S15:** Cartesian Coordinates for the low-energy geometry of the **EEEA** tetrapeptide calculated using the B3LYP method and the 6-311+G(*2df,2pd*) level of theory.

Standard Orientation					
Center Number	Atomic Number	Atomic Type	Coordinates (Angstroms)		
			X	Y	Z
1	6	0	4.591312	-0.954277	-0.079638
2	6	0	5.866910	-0.269510	0.448095
3	6	0	7.096610	-1.176489	0.623999
4	6	0	7.938639	-1.612041	-0.616876
5	8	0	9.001658	-2.214702	-0.339081
6	8	0	7.496327	-1.330993	-1.761886
7	1	0	7.802548	-0.683159	1.298355
8	1	0	6.802877	-2.095156	1.143045
9	1	0	6.109032	0.560745	-0.220522
10	1	0	5.620297	0.159343	1.421600
11	7	0	4.638755	-1.082390	-1.539777
12	1	0	4.109900	-1.890013	-1.844254
13	1	0	5.617270	-1.195868	-1.828790
14	6	0	3.328111	-0.204579	0.403439
15	8	0	2.975245	-0.261752	1.577342
16	7	0	2.685436	0.490914	-0.558461
17	6	0	1.367254	1.096598	-0.425041
18	6	0	1.359828	2.298462	0.551696
19	6	0	0.236311	3.331017	0.375504
20	6	0	0.613073	4.814252	0.755330
21	8	0	1.809201	5.145664	0.605866
22	8	0	-0.355475	5.518981	1.129089
23	1	0	-0.075865	3.372891	-0.674998
24	1	0	-0.662241	3.100524	0.954752
25	1	0	2.290101	2.839852	0.379765
26	1	0	1.401170	1.931994	1.577627
27	6	0	0.303210	-0.035788	-0.249027
28	8	0	0.517751	-1.158125	-0.671126
29	7	0	-0.862500	0.359003	0.337465
30	6	0	-2.111423	-0.382445	0.520576
31	1	0	-2.572622	0.068357	1.403620
32	6	0	-3.077468	-0.187264	-0.678137
33	8	0	-2.800528	-0.579356	-1.798952
34	7	0	-4.238082	0.442430	-0.346089
35	6	0	-5.437161	0.559071	-1.179872
36	1	0	-6.081166	1.256442	-0.634233
37	6	0	-6.298500	-0.754662	-1.255257
38	8	0	-7.143385	-0.801818	-2.157847
39	8	0	-6.110937	-1.622089	-0.347044
40	6	0	-5.155323	1.177552	-2.548522
41	1	0	-4.647946	2.137488	-2.432885
42	1	0	-6.100521	1.324512	-3.066371
43	1	0	-4.523286	0.526508	-3.145481
44	1	0	-4.389118	0.578372	0.638528
45	6	0	-1.922906	-1.878970	0.813844
46	6	0	-3.245867	-2.633417	1.047665
47	1	0	-2.995867	-3.675390	1.255722
48	1	0	-3.862476	-2.611332	0.150277

49	6	0	-4.040517	-2.133799	2.250131
50	8	0	-3.627107	-2.260693	3.387189
51	8	0	-5.201358	-1.543029	2.003768
52	1	0	-5.496261	-1.552614	1.010937
53	1	0	-1.281416	-1.975134	1.689848
54	1	0	-1.401647	-2.332434	-0.023355
55	1	0	-0.905640	1.329401	0.603087
56	1	0	1.139249	1.502077	-1.416765
57	1	0	3.062307	0.320594	-1.481376
58	1	0	4.512389	-1.926652	0.423918

16. **Table S16:** Cartesian Coordinates for the low-energy geometry of the **EGED** tetrapeptide calculated using the B3LYP method and the 6-311+G(*2df,2pd*) level of theory.

Standard Orientation					
Center Number	Atomic Number	Atomic Type	Coordinates (Angstroms)		
			X	Y	Z
1	6	0	5.636921	0.053937	0.514722
2	7	0	5.511749	0.767783	-0.755230
3	1	0	6.406666	0.702624	-1.253399
4	1	0	5.298026	1.745832	-0.605783
5	1	0	6.036259	0.686252	1.320872
6	6	0	6.596007	-1.146072	0.372263
7	6	0	8.091779	-0.832138	0.529508
8	6	0	8.865714	-0.117808	-0.623238
9	8	0	10.095383	0.008029	-0.422443
10	8	0	8.209849	0.253806	-1.631248
11	1	0	8.249663	-0.237592	1.435261
12	1	0	8.629281	-1.768583	0.707168
13	1	0	6.413654	-1.608438	-0.601508
14	1	0	6.319386	-1.871925	1.139311
15	6	0	4.277530	-0.412644	1.070747
16	8	0	4.189903	-0.874359	2.210400
17	7	0	3.216625	-0.265355	0.256429
18	1	0	3.363635	0.116330	-0.670255
19	6	0	1.875410	-0.618961	0.653596
20	6	0	0.845424	-0.076269	-0.348011
21	8	0	1.204239	0.445959	-1.401996
22	7	0	-0.430166	-0.238670	0.033185
23	6	0	-1.606894	0.028637	-0.794287
24	6	0	-2.768196	-0.853315	-0.252974
25	7	0	-3.781097	-0.960344	-1.046045
26	6	0	-5.023242	-1.669097	-0.691024
27	6	0	-4.907281	-3.120760	-0.183217
28	8	0	-5.642475	-3.997439	-0.608029
29	8	0	-4.011428	-3.394681	0.757601
30	1	0	-3.429650	-2.566063	0.940568
31	1	0	-5.579023	-1.773561	-1.622818
32	6	0	-5.960913	-0.858356	0.250565
33	1	0	-6.749300	-1.524853	0.605257
34	1	0	-5.384362	-0.521907	1.144601
35	6	0	-6.655587	0.337786	-0.396133
36	8	0	-7.450871	0.964757	0.594754
37	1	0	-6.853903	1.238323	1.338831
38	8	0	-5.620292	1.332597	-0.708593
39	1	0	-4.926056	0.835682	-1.165579
40	8	0	-2.557330	-1.369688	0.913239
41	1	0	-1.389458	-0.287065	-1.813839
42	6	0	-1.944281	1.530281	-0.847235
43	1	0	-1.037010	2.059176	-1.144220
44	1	0	-2.679115	1.681212	-1.636181
45	6	0	-2.494546	2.091947	0.471735
46	1	0	-1.964761	1.697102	1.336700
47	1	0	-3.551521	1.795493	0.571295
48	6	0	-2.477288	3.581887	0.589630

49	8	0	-2.312099	4.221663	1.607510
50	8	0	-2.716651	4.224437	-0.587380
51	1	0	-2.755186	5.164118	-0.364245
52	1	0	-0.675114	-0.782016	0.856935
53	1	0	1.758629	-1.706163	0.718675
54	1	0	1.670315	-0.232859	1.653586

17. **Table S17:** Cartesian Coordinates for the low-energy geometry of the **EDEA** tetrapeptide calculated using the B3LYP method and the 6-311+G(*2df,2pd*) level of theory.

Standard Orientation					
Center Number	Atomic Number	Atomic Type	Coordinates (Angstroms)		
			X	Y	Z
1	6	0	5.025402	0.662809	0.205199
2	7	0	5.128694	0.175579	1.587971
3	1	0	4.621063	0.792091	2.213008
4	1	0	6.121665	0.250360	1.816256
5	1	0	5.218939	1.740910	0.131980
6	6	0	6.074437	-0.044865	-0.667338
7	6	0	7.574236	0.211447	-0.300771
8	8	0	7.825633	0.667032	0.849057
9	8	0	8.397600	-0.091734	-1.192918
10	1	0	5.911667	-1.126058	-0.608569
11	1	0	5.927335	0.240641	-1.707608
12	6	0	3.608337	0.495343	-0.377441
13	8	0	3.273684	1.103686	-1.391572
14	7	0	2.792522	-0.328364	0.315521
15	1	0	3.188439	-0.798258	1.118566
16	6	0	1.391709	-0.612451	-0.003568
17	1	0	0.996322	0.231249	-0.561893
18	6	0	0.694898	-0.779673	1.357872
19	8	0	1.246346	-1.441183	2.239275
20	7	0	-0.491203	-0.174105	1.615825
21	6	0	-1.462005	0.497227	0.751926
22	1	0	-1.126718	0.406912	-0.276268
23	6	0	-2.811415	-0.230581	0.918855
24	8	0	-3.367433	-0.236971	2.021049
25	7	0	-3.291806	-0.767449	-0.208179
26	6	0	-4.614113	-1.366270	-0.348379
27	1	0	-4.657653	-1.690982	-1.391527
28	6	0	-5.794871	-0.335814	-0.224722
29	8	0	-6.919071	-0.819822	-0.013914
30	8	0	-5.531135	0.888708	-0.416734
31	6	0	-4.804966	-2.604844	0.532927
32	1	0	-4.025605	-3.338391	0.317177
33	1	0	-5.783368	-3.039682	0.337088
34	1	0	-4.747998	-2.332584	1.585019
35	1	0	-2.607635	-0.907372	-0.959235
36	6	0	-1.622983	1.973443	1.178065
37	6	0	-2.897762	2.690154	0.690624
38	1	0	-2.941933	3.655063	1.200974
39	1	0	-3.778447	2.119165	0.978461
40	6	0	-2.935938	3.021149	-0.799026
41	8	0	-2.213809	3.883671	-1.268045
42	8	0	-3.803842	2.366118	-1.544236
43	1	0	-4.406206	1.700028	-1.041791
44	1	0	-0.738093	2.526165	0.857523
45	1	0	-1.644245	1.993621	2.269844
46	1	0	-0.855408	-0.411232	2.529797
47	6	0	1.261063	-1.888919	-0.863027
48	6	0	-0.126965	-2.216347	-1.486015

49	8	0	-0.318599	-3.411997	-1.781106
50	8	0	-0.907902	-1.240267	-1.674798
51	1	0	1.954352	-1.770035	-1.699188
52	1	0	1.599917	-2.750011	-0.287120

18. **Table S18:** Cartesian Coordinates for the low-energy geometry of the **EAQG** tetrapeptide calculated using the B3LYP method and the 6-311+G(*2df,2pd*) level of theory.

Standard Orientation					
Center Number	Atomic Number	Atomic Type	Coordinates (Angstroms)		
			X	Y	Z
1	6	0	3.385515	1.963532	0.676394
2	7	0	3.276916	2.207009	2.117392
3	1	0	3.747331	3.066105	2.372396
4	1	0	3.726346	1.441442	2.607753
5	1	0	4.417720	2.114989	0.318164
6	6	0	3.202366	0.447037	0.457753
7	8	0	3.835397	-0.325267	1.171608
8	7	0	2.412290	0.048737	-0.557543
9	6	0	2.373966	-1.325342	-1.052365
10	1	0	1.577701	-1.326218	-1.798460
11	6	0	1.990890	-2.383570	0.007175
12	8	0	2.726495	-3.329719	0.238590
13	7	0	0.798822	-2.269781	0.658483
14	6	0	-0.407235	-1.497392	0.407808
15	1	0	-0.144170	-0.499678	0.060807
16	6	0	-1.364170	-2.202539	-0.617912
17	6	0	-1.395459	-1.616943	-2.043415
18	6	0	-2.376765	-0.462557	-2.184251
19	7	0	-1.898944	0.698996	-2.624342
20	1	0	-0.902449	0.960800	-2.494891
21	1	0	-2.521386	1.493542	-2.576055
22	8	0	-3.572726	-0.641780	-1.887808
23	1	0	-0.404978	-1.320007	-2.381996
24	1	0	-1.753283	-2.392421	-2.724199
25	1	0	-2.388849	-2.212567	-0.244929
26	1	0	-1.047759	-3.243549	-0.663964
27	6	0	-1.125013	-1.409111	1.776932
28	8	0	-0.905182	-2.249849	2.639347
29	7	0	-2.018250	-0.414195	1.990486
30	1	0	-2.470615	-0.451286	2.893619
31	6	0	-2.532036	0.596371	1.080400
32	1	0	-2.149900	1.589853	1.327236
33	1	0	-2.223500	0.389822	0.067013
34	6	0	-4.058274	0.662402	1.224800
35	8	0	-4.552407	0.830062	2.314711
36	8	0	-4.799302	0.555235	0.130325
37	1	0	-4.305782	0.212074	-0.662993
38	1	0	0.678239	-2.948431	1.400574
39	6	0	3.677431	-1.716413	-1.749077
40	1	0	3.890393	-0.999399	-2.542550
41	1	0	3.598896	-2.713290	-2.179712
42	1	0	4.497426	-1.723492	-1.035670
43	1	0	1.813752	0.727460	-1.053839
44	6	0	2.514878	2.945018	-0.120634
45	6	0	1.008644	2.968238	0.197946
46	1	0	0.687564	3.959218	0.513043
47	1	0	0.806759	2.314076	1.050979
48	6	0	0.070948	2.529874	-0.939721

49	8	0	0.520060	1.638291	-1.747809
50	8	0	-1.066645	3.019929	-0.978589
51	1	0	2.664053	2.759675	-1.185299
52	1	0	2.931015	3.939090	0.067627

19. **Table S19:** Cartesian Coordinates for the low-energy geometry of the **QGPK** tetrapeptide calculated using the B3LYP method and the 6-311+G(*2df,2pd*) level of theory.

Standard Orientation					
Center Number	Atomic Number	Atomic Type	Coordinates (Angstroms)		
			X	Y	Z
1	6	0	3.792555	1.461862	0.046739
2	7	0	5.066165	2.086983	0.372405
3	1	0	5.573394	2.336366	-0.467393
4	1	0	4.894562	2.953870	0.871992
5	1	0	3.972051	0.742369	-0.752177
6	6	0	3.253469	0.710896	1.283304
7	6	0	4.094146	-0.506230	1.649722
8	6	0	3.939624	-1.663249	0.674073
9	7	0	4.847327	-2.656585	0.802366
10	1	0	5.602634	-2.605326	1.462361
11	1	0	4.788546	-3.457632	0.196494
12	8	0	3.045107	-1.715567	-0.166068
13	1	0	5.145549	-0.221491	1.717547
14	1	0	3.804311	-0.878238	2.636267
15	1	0	2.219662	0.401761	1.127641
16	1	0	3.251794	1.411583	2.120021
17	6	0	2.736273	2.482532	-0.411717
18	8	0	2.761222	3.633673	-0.012965
19	7	0	1.753056	2.066614	-1.267668
20	1	0	1.067836	2.774593	-1.486406
21	6	0	1.489889	0.739405	-1.761182
22	1	0	1.832965	0.610369	-2.797430
23	1	0	2.009809	-0.011460	-1.168151
24	6	0	-0.002340	0.445961	-1.752141
25	8	0	-0.823854	1.362996	-1.577659
26	7	0	-0.391273	-0.820061	-1.965242
27	6	0	-1.816929	-1.141370	-2.123266
28	6	0	-1.811496	-2.601157	-2.634414
29	6	0	-0.455220	-3.161990	-2.186825
30	6	0	0.489610	-1.966866	-2.294893
31	1	0	0.867661	-1.855132	-3.314292
32	1	0	1.332954	-2.022285	-1.612014
33	1	0	-0.499391	-3.513488	-1.155269
34	1	0	-0.126963	-3.997488	-2.800800
35	1	0	-1.878897	-2.592385	-3.721921
36	1	0	-2.660168	-3.169565	-2.260667
37	1	0	-2.271172	-0.472451	-2.852073
38	6	0	-2.654004	-0.953578	-0.848211
39	8	0	-3.805145	-0.523356	-0.947341
40	7	0	-2.075152	-1.295938	0.313096
41	1	0	-1.095307	-1.547347	0.317524
42	6	0	-2.688558	-1.256613	1.637310
43	6	0	-3.487980	0.027755	1.947461
44	1	0	-4.381376	-0.013796	1.331223
45	1	0	-3.815149	-0.034431	2.984470
46	6	0	-2.739535	1.344958	1.688485
47	1	0	-2.146769	1.619876	2.560945

48	1	0	-2.014953	1.198733	0.886509
49	6	0	-3.697296	2.504508	1.323461
50	1	0	-4.724660	2.136980	1.266549
51	6	0	-3.372698	3.225473	0.021643
52	1	0	-2.397516	3.707078	0.055121
53	1	0	-4.122309	3.985003	-0.188770
54	7	0	-3.339126	2.283907	-1.165669
55	1	0	-3.640290	2.756706	-2.014542
56	1	0	-2.373914	1.912682	-1.340108
57	1	0	-3.920534	1.441177	-1.042473
58	1	0	-3.701628	3.265682	2.103969
59	1	0	-3.385270	-2.093758	1.741770
60	6	0	-1.562923	-1.509738	2.634924
61	8	0	-0.404306	-1.641145	2.331023
62	8	0	-2.022517	-1.585189	3.888201
63	1	0	-1.278016	-1.769641	4.480706

20. **Table S20:** Cartesian Coordinates for the low-energy geometry of the **AEEE** tetrapeptide calculated using the B3LYP method and the 6-311+G(*2df,2pd*) level of theory.

Standard Orientation					
Center Number	Atomic Number	Atomic Type	Coordinates (Angstroms)		
			X	Y	Z
1	6	0	4.681788	-1.010717	1.458990
2	7	0	5.155825	-2.390477	1.385528
3	1	0	5.568234	-2.525203	0.467145
4	1	0	4.356312	-3.026562	1.438666
5	1	0	3.891216	-0.978922	2.208959
6	6	0	4.104071	-0.513521	0.118664
7	8	0	4.638555	-0.813094	-0.945696
8	7	0	3.026731	0.301022	0.213574
9	1	0	2.623972	0.425151	1.128011
10	6	0	2.368638	0.942924	-0.914403
11	6	0	2.162480	2.452381	-0.659317
12	1	0	1.689784	2.609861	0.313293
13	6	0	3.466935	3.250714	-0.720770
14	1	0	4.216172	2.772382	-0.080531
15	1	0	3.871888	3.230149	-1.734826
16	6	0	3.386751	4.739567	-0.251210
17	8	0	4.182079	5.525581	-0.826803
18	8	0	2.578566	4.993560	0.671681
19	1	0	1.464064	2.824456	-1.409810
20	6	0	1.080198	0.234984	-1.388069
21	8	0	0.617094	0.516903	-2.487588
22	7	0	0.513253	-0.703807	-0.580316
23	6	0	-0.419210	-1.669007	-1.152741
24	6	0	-0.514620	-2.933063	-0.272580
25	6	0	0.772367	-3.756980	-0.132942
26	6	0	1.756809	-3.347833	0.994572
27	8	0	2.637271	-4.190230	1.279565
28	8	0	1.606178	-2.206561	1.510325
29	1	0	0.518431	-4.804848	0.041923
30	1	0	1.339407	-3.744103	-1.069333
31	1	0	-0.869514	-2.648285	0.719610
32	1	0	-1.291162	-3.553583	-0.723475
33	6	0	-1.853626	-1.182583	-1.411463
34	7	0	-2.411791	-0.324103	-0.527538
35	6	0	-3.806089	0.085990	-0.640075
36	6	0	-4.243482	0.918924	0.588979
37	6	0	-5.607981	1.596316	0.440870
38	6	0	-6.300801	2.031545	1.778691
39	8	0	-7.177387	2.920136	1.647999
40	8	0	-5.952705	1.426463	2.818515
41	1	0	-5.540430	2.469877	-0.210107
42	1	0	-6.314460	0.915857	-0.042257
43	1	0	-3.473550	1.677826	0.754879
44	1	0	-4.260863	0.287820	1.477970
45	6	0	-4.731700	-1.115214	-0.793249
46	8	0	-4.431696	-2.126298	0.056559
47	1	0	-5.101055	-2.805386	-0.097407
48	8	0	-5.696509	-1.161568	-1.516143

49	1	0	-3.943873	0.667728	-1.552972
50	1	0	-1.840893	0.067293	0.203934
51	8	0	-2.500753	-1.663074	-2.336717
52	1	0	-0.065109	-1.951017	-2.146750
53	1	0	1.015358	-1.054424	0.247486
54	1	0	3.045335	0.821807	-1.759824
55	6	0	5.824466	-0.072165	1.871972
56	1	0	6.627073	-0.114479	1.132775
57	1	0	6.226609	-0.394203	2.833554
58	1	0	5.491410	0.964233	1.951277

21. **Table S21:** Cartesian Coordinates for the low-energy geometry of the **EEVA** tetrapeptide calculated using the B3LYP method and the 6-311+G(*2df,2pd*) level of theory.

Standard Orientation					
Center Number	Atomic Number	Atomic Type	Coordinates (Angstroms)		
			X	Y	Z
1	6	0	-4.040357	-0.410818	-0.168444
2	6	0	-4.976629	0.474058	-1.014538
3	6	0	-6.320354	-0.163143	-1.398599
4	6	0	-7.469025	-0.222369	-0.341871
5	8	0	-8.560980	-0.637410	-0.781132
6	8	0	-7.201682	0.149054	0.833978
7	1	0	-6.734125	0.375635	-2.255088
8	1	0	-6.159637	-1.185987	-1.754814
9	1	0	-5.155127	1.402739	-0.465856
10	1	0	-4.436077	0.731633	-1.927367
11	7	0	-4.481520	-0.464283	1.228262
12	1	0	-4.346875	-1.388823	1.616035
13	1	0	-5.486664	-0.235719	1.271300
14	6	0	-2.590218	0.100953	-0.297573
15	7	0	-2.097674	0.650694	0.826908
16	6	0	-0.805407	1.324516	1.019889
17	6	0	-0.623208	2.508909	0.049903
18	6	0	0.490942	3.488351	0.426958
19	6	0	1.914016	3.172731	-0.103750
20	8	0	2.007235	2.270547	-0.984070
21	8	0	2.839465	3.862083	0.371840
22	1	0	0.242170	4.475678	0.026217
23	1	0	0.562503	3.620898	1.509397
24	1	0	-1.584053	3.031514	0.049242
25	1	0	-0.452402	2.147512	-0.961285
26	6	0	0.351336	0.295430	1.084758
27	8	0	0.974054	0.118337	2.125319
28	7	0	0.576829	-0.401742	-0.047819
29	6	0	1.497321	-1.522561	-0.156516
30	1	0	1.519698	-1.752535	-1.226516
31	6	0	2.965918	-1.165689	0.191630
32	8	0	3.693155	-1.980562	0.758606
33	7	0	3.440911	0.009044	-0.262511
34	1	0	2.829305	0.776519	-0.593206
35	6	0	4.824137	0.341955	0.019847
36	6	0	5.199350	1.706895	-0.572674
37	1	0	5.087635	1.698018	-1.657299
38	1	0	6.236549	1.933561	-0.322213
39	1	0	4.545085	2.485975	-0.178627
40	6	0	5.808824	-0.693452	-0.503835
41	8	0	5.438911	-1.265475	-1.675790
42	1	0	6.152196	-1.877272	-1.901335
43	8	0	6.883714	-0.929601	-0.003776
44	1	0	4.993478	0.358363	1.098586
45	6	0	1.046270	-2.816936	0.581238
46	6	0	-0.476638	-2.958997	0.640250
47	1	0	-0.739354	-3.910876	1.108322
48	1	0	-0.923004	-2.944577	-0.357051

49	1	0	-0.940130	-2.164351	1.220597
50	6	0	1.657364	-4.054082	-0.090151
51	1	0	1.270562	-4.169857	-1.108117
52	1	0	1.394145	-4.958724	0.464082
53	1	0	2.741417	-3.983055	-0.132473
54	1	0	1.430613	-2.757253	1.599429
55	1	0	-0.058672	-0.226520	-0.818690
56	1	0	-0.840623	1.722559	2.033114
57	1	0	-2.761214	0.605678	1.594474
58	8	0	-1.969163	-0.011819	-1.358335
59	1	0	-4.016747	-1.402480	-0.636995

22. **Table S22:** Cartesian Coordinates for the low-energy geometry of the **EEEI** tetrapeptide calculated using the B3LYP method and the 6-311+G(*2df,2pd*) level of theory.

**Standard Orientation**					
Center Number	Atomic Number	Atomic Type	Coordinates (Angstroms)		
			X	Y	Z
1	6	0	-3.983945	-0.370521	-0.261349
2	6	0	-4.801243	0.326687	-1.366053
3	6	0	-5.994167	-0.469494	-1.923223
4	6	0	-7.347409	-0.484682	-1.144365
5	8	0	-8.318835	-0.938845	-1.789309
6	8	0	-7.349692	-0.055594	0.041062
7	1	0	-6.231226	-0.096385	-2.922616
8	1	0	-5.703443	-1.515430	-2.070246
9	1	0	-5.149087	1.287927	-0.978201
10	1	0	-4.112417	0.538567	-2.185681
11	7	0	-4.693670	-0.323159	1.020484
12	1	0	-4.540945	-1.170679	1.551355
13	1	0	-5.702965	-0.220608	0.844895
14	6	0	-2.570949	0.263907	-0.181253
15	7	0	-2.377473	1.009807	0.925266
16	6	0	-1.279328	1.897393	1.334967
17	6	0	-0.510050	2.599403	0.198216
18	6	0	0.430734	3.695828	0.708954
19	6	0	0.866432	4.752598	-0.363245
20	8	0	2.016550	5.233128	-0.206368
21	8	0	0.014689	5.049736	-1.233369
22	1	0	-0.071610	4.266613	1.498730
23	1	0	1.328143	3.274592	1.163182
24	1	0	-1.242129	3.062939	-0.463893
25	1	0	0.038102	1.883242	-0.410213
26	6	0	-0.386346	1.271857	2.435769
27	8	0	-0.375034	1.794857	3.549630
28	7	0	0.379953	0.174724	2.205051
29	6	0	0.587240	-0.655187	1.028929
30	1	0	-0.033136	-0.293742	0.212432
31	6	0	0.222573	-2.123780	1.358696
32	1	0	0.926418	-2.464807	2.121194
33	1	0	-0.768052	-2.136958	1.812113
34	6	0	0.258363	-3.073367	0.137419
35	1	0	-0.731632	-3.120028	-0.314199
36	1	0	0.960546	-2.709271	-0.607513
37	6	0	0.660755	-4.485761	0.536068
38	8	0	1.906046	-4.852753	0.262538
39	1	0	2.450006	-4.153694	-0.237553
40	8	0	-0.106478	-5.247320	1.096746
41	6	0	2.084457	-0.615160	0.662934
42	8	0	2.934533	-0.721815	1.539680
43	7	0	2.363153	-0.508278	-0.649819
44	1	0	1.577324	-0.450992	-1.274316
45	6	0	3.682496	-0.754532	-1.241951
46	6	0	4.740490	0.324714	-0.902071
47	6	0	4.127348	1.734776	-0.848182
48	6	0	5.082178	2.853427	-0.427685

49	1	0	4.525495	3.785638	-0.319836
50	1	0	5.883087	3.014380	-1.154005
51	1	0	5.548960	2.622603	0.534703
52	1	0	3.292445	1.737808	-0.151299
53	1	0	3.697004	1.983787	-1.824897
54	6	0	5.885558	0.252575	-1.922152
55	1	0	6.286304	-0.757374	-1.969284
56	1	0	5.530489	0.542759	-2.918035
57	1	0	6.695233	0.933801	-1.653063
58	1	0	5.144157	0.081924	0.082685
59	1	0	3.504334	-0.705462	-2.320924
60	6	0	4.123962	-2.253296	-0.981782
61	8	0	3.172994	-3.085255	-1.110024
62	8	0	5.312743	-2.495025	-0.751846
63	1	0	1.001144	-0.037203	2.974016
64	1	0	-1.771419	2.692702	1.894621
65	1	0	-3.194715	0.981717	1.528150
66	8	0	-1.747814	0.061836	-1.072080
67	1	0	-3.792678	-1.397915	-0.594297

23. **Table S23:** Cartesian Coordinates for the low-energy geometry of the **EIDG** tetrapeptide calculated using the B3LYP method and the 6-311+G(*2df,2pd*) level of theory.

Standard Orientation					
Center Number	Atomic Number	Atomic Type	Coordinates (Angstroms)		
			X	Y	Z
1	6	0	3.641026	0.449818	0.467123
2	7	0	3.242519	1.302024	-0.655595
3	1	0	3.980591	1.284679	-1.374059
4	1	0	3.101784	2.258863	-0.360153
5	1	0	4.040476	1.031427	1.307239
6	6	0	4.727515	-0.560328	0.050536
7	1	0	4.755294	-1.328890	0.824500
8	1	0	4.409190	-1.039300	-0.880002
9	6	0	6.140283	0.014453	-0.133144
10	1	0	6.354624	0.739374	0.659640
11	1	0	6.872200	-0.785497	0.003721
12	6	0	6.503095	0.700383	-1.488142
13	8	0	5.555397	0.963723	-2.276580
14	8	0	7.720935	0.924084	-1.653602
15	6	0	2.444304	-0.318215	1.080780
16	8	0	2.579399	-0.983058	2.100887
17	7	0	1.300652	-0.195910	0.362465
18	1	0	1.439928	0.386988	-0.459315
19	6	0	-0.003369	-0.816353	0.594853
20	6	0	0.069009	-2.295133	1.041805
21	6	0	0.899133	-3.133544	0.056177
22	6	0	1.286993	-4.515086	0.587301
23	1	0	1.910011	-5.048313	-0.134165
24	1	0	0.416896	-5.142076	0.793777
25	1	0	1.858705	-4.420166	1.512292
26	1	0	1.812421	-2.595254	-0.190821
27	1	0	0.344031	-3.242916	-0.883400
28	6	0	-1.342238	-2.872900	1.205103
29	1	0	-1.917199	-2.312616	1.940723
30	1	0	-1.884101	-2.861327	0.254168
31	1	0	-1.301548	-3.908718	1.544983
32	1	0	0.564689	-2.303936	2.010858
33	1	0	-0.500828	-0.793335	-0.376072
34	6	0	-0.821724	0.037279	1.607253
35	8	0	-0.719686	-0.170005	2.806790
36	7	0	-1.665734	0.997601	1.125222
37	6	0	-1.770245	1.492317	-0.242637
38	1	0	-0.792424	1.721602	-0.671336
39	6	0	-2.441103	0.510372	-1.206176
40	8	0	-2.228143	0.515642	-2.409936
41	7	0	-3.347499	-0.335593	-0.629359
42	1	0	-3.448761	-0.290186	0.372591
43	6	0	-4.089247	-1.279993	-1.401897
44	1	0	-3.860099	-1.098123	-2.453684
45	1	0	-3.796865	-2.316604	-1.194949
46	6	0	-5.585177	-1.198598	-1.205034
47	8	0	-6.222302	-2.095515	-2.009738
48	1	0	-7.166759	-2.003357	-1.823456

49	8	0	-6.183558	-0.487497	-0.445280
50	6	0	-2.639639	2.781759	-0.280492
51	6	0	-2.394162	3.863039	0.825874
52	8	0	-2.410027	5.041927	0.438629
53	8	0	-2.279171	3.422985	2.003747
54	1	0	-3.689879	2.492871	-0.175295
55	1	0	-2.520389	3.237786	-1.259526
56	1	0	-1.941040	1.746279	1.797573

24. **Table S24:** Cartesian Coordinates for the low-energy geometry of the **PEDK** tetrapeptide calculated using the B3LYP method and the 6-311+G(*2df,2pd*) level of theory.

Standard Orientation					
Center Number	Atomic Number	Atomic Type	Coordinates (Angstroms)		
			X	Y	Z
1	6	0	-4.953704	-3.529357	-0.313401
2	7	0	-3.850469	-2.953573	0.454357
3	1	0	-3.766495	-3.324203	1.391227
4	6	0	-4.102532	-1.511304	0.455220
5	1	0	-4.983620	-1.257308	1.070355
6	6	0	-4.446048	-1.235650	-1.020100
7	6	0	-5.110459	-2.552508	-1.498715
8	1	0	-6.159923	-2.412746	-1.755534
9	1	0	-4.609259	-2.936552	-2.386628
10	1	0	-5.076224	-0.358238	-1.134140
11	1	0	-3.528259	-1.043085	-1.573500
12	6	0	-2.934805	-0.735805	1.053996
13	8	0	-2.137758	-1.325345	1.828801
14	7	0	-2.860458	0.544013	0.756400
15	1	0	-3.619478	1.072802	0.158064
16	6	0	-1.830811	1.402442	1.298107
17	6	0	-2.259117	2.879791	1.139685
18	6	0	-2.576891	3.360220	-0.290839
19	6	0	-4.031503	3.122330	-0.782512
20	8	0	-4.541666	1.978396	-0.510544
21	8	0	-4.577509	4.034997	-1.404395
22	1	0	-1.902868	2.880303	-1.006624
23	1	0	-2.390263	4.430201	-0.360071
24	1	0	-1.462159	3.501716	1.552637
25	1	0	-3.132073	3.036076	1.777166
26	1	0	-1.710968	1.186421	2.364919
27	6	0	-0.453301	1.127919	0.644097
28	8	0	-0.317013	0.469642	-0.377605
29	7	0	0.595134	1.672257	1.308926
30	6	0	1.963037	1.755943	0.788733
31	6	0	2.508404	0.340612	0.490283
32	7	0	3.370822	0.262651	-0.508583
33	6	0	4.025444	-0.960553	-0.926234
34	6	0	3.070277	-2.048744	-1.494479
35	1	0	3.543637	-2.491110	-2.371998
36	1	0	2.202617	-1.501132	-1.862172
37	6	0	2.636161	-3.188420	-0.553931
38	1	0	3.338240	-4.015748	-0.647463
39	6	0	1.218813	-3.712678	-0.826366
40	1	0	1.106364	-3.965884	-1.884328
41	6	0	0.082562	-2.760649	-0.461013
42	1	0	0.102171	-1.838805	-1.037054
43	7	0	0.144655	-2.338151	0.970728
44	1	0	0.322048	-3.132770	1.579162
45	1	0	-0.769392	-1.902077	1.282166
46	1	0	0.910231	-1.631209	1.113115
47	1	0	-0.887519	-3.230345	-0.617653
48	1	0	1.077619	-4.651910	-0.279865

49	1	0	2.706455	-2.856817	0.480701
50	6	0	5.042482	-1.527671	0.062571
51	8	0	5.546030	-2.620382	-0.048109
52	8	0	5.398656	-0.665016	1.028241
53	1	0	6.084757	-1.101064	1.552937
54	1	0	4.661398	-0.645100	-1.759477
55	1	0	3.763763	1.208634	-0.921737
56	8	0	2.178608	-0.622458	1.213412
57	1	0	2.568201	2.107689	1.629419
58	6	0	2.060605	2.800853	-0.345750
59	6	0	3.489728	3.343434	-0.640769
60	8	0	3.734345	4.505643	-0.314048
61	8	0	4.285507	2.520612	-1.211621
62	1	0	1.455176	3.653421	-0.047648
63	1	0	1.629804	2.378444	-1.254227
64	1	0	0.394294	2.213692	2.131514
65	1	0	-5.890781	-3.565074	0.263960
66	1	0	-4.718934	-4.548527	-0.624567

25. **Table S25:** Cartesian Coordinates for the low-energy geometry of the **DKGH** tetrapeptide calculated using the B3LYP method and the 6-311+G(*2df,2pd*) level of theory.

Standard Orientation					
Center Number	Atomic Number	Atomic Type	Coordinates (Angstroms)		
			X	Y	Z
1	6	0	-2.237798	-0.892019	-0.905883
2	7	0	-2.137856	0.419566	-0.234206
3	6	0	-3.046219	1.318693	0.139791
4	6	0	-2.476697	2.651249	0.692243
5	7	0	-3.564594	3.618030	0.762223
6	1	0	-4.426495	3.173747	1.043110
7	1	0	-3.348904	4.363915	1.409464
8	1	0	-2.075590	2.384935	1.681869
9	6	0	-1.333866	3.300724	-0.121588
10	6	0	0.060180	2.759844	0.110686
11	8	0	1.001454	3.638963	-0.100566
12	8	0	0.268534	1.598339	0.453006
13	1	0	-1.320932	4.363707	0.112826
14	1	0	-1.541964	3.253820	-1.194645
15	8	0	-4.281832	1.162719	0.125077
16	1	0	-1.183060	0.767715	-0.128002
17	6	0	-3.628909	-1.527041	-0.985869
18	1	0	-3.517113	-2.396837	-1.632720
19	1	0	-4.281987	-0.826416	-1.502679
20	6	0	-4.216892	-1.978564	0.351450
21	1	0	-3.616754	-2.797005	0.749160
22	6	0	-5.667092	-2.469572	0.252515
23	1	0	-5.722031	-3.361445	-0.375644
24	6	0	-6.651608	-1.468050	-0.336281
25	1	0	-6.452826	-1.264013	-1.385863
26	7	0	-6.563134	-0.135508	0.365445
27	1	0	-6.744518	-0.235113	1.363577
28	1	0	-7.246531	0.523080	-0.005515
29	1	0	-5.600224	0.305861	0.254318
30	1	0	-7.677498	-1.819679	-0.245501
31	1	0	-6.011070	-2.778453	1.243244
32	1	0	-4.152491	-1.171634	1.084439
33	6	0	-1.212675	-1.939140	-0.390359
34	8	0	-1.328251	-3.086753	-0.778038
35	7	0	-0.220326	-1.523266	0.435690
36	6	0	0.951633	-2.345359	0.680132
37	1	0	0.720863	-3.360306	0.360552
38	1	0	1.183322	-2.360599	1.745407
39	6	0	2.132399	-1.821479	-0.152723
40	8	0	1.986374	-1.462092	-1.302306
41	7	0	3.328368	-1.753935	0.494000
42	1	0	3.452337	-2.075440	1.442383
43	6	0	4.476021	-1.137682	-0.132544
44	1	0	4.628711	-1.565267	-1.128028
45	6	0	4.271445	0.350264	-0.271205
46	6	0	3.348188	1.154884	0.323496
47	7	0	3.395242	2.416127	-0.220045
48	6	0	4.363177	2.390846	-1.110946

49	7	0	4.924374	1.158617	-1.175836
50	1	0	5.698096	0.892398	-1.762244
51	1	0	4.681034	3.213278	-1.728245
52	1	0	1.947001	3.223462	-0.085525
53	1	0	2.610489	0.894584	1.056771
54	6	0	5.709654	-1.459985	0.709904
55	8	0	5.679868	-1.978027	1.791200
56	8	0	6.830294	-1.066564	0.083787
57	1	0	7.588381	-1.260669	0.655284
58	1	0	-0.106222	-0.540345	0.625097
59	1	0	-1.915697	-0.723045	-1.937979

26. **Table S26:** Cartesian Coordinates for the low-energy geometry of the **EAEE+EA** hexapeptide calculated using the B3LYP method and the 6-311+G(*2df,2pd*) level of theory. Final Energy = -1550200 kcal/mol, Relative Energy = 24661 kcal/mol, Net Charge = -4, Multiplicity = 1.

Standard Orientation					
Center Number	Atomic Number	Atomic Type	Coordinates (Angstroms)		
			X	Y	Z
1	6	0	2.553524	-0.355534	0.975038
2	7	0	1.344759	-0.883399	0.367472
3	1	0	0.570000	-0.258430	0.197069
4	6	0	1.254055	-2.178376	-0.048769
5	6	0	-0.062016	-2.540170	-0.782598
6	7	0	-1.243244	-1.947225	-0.140903
7	6	0	-1.690550	-2.260563	1.094701
8	6	0	-3.020206	-1.603340	1.544782
9	7	0	-3.522228	-0.572531	0.650213
10	6	0	-3.097753	0.714098	0.747477
11	6	0	-3.765225	1.732692	-0.210898
12	7	0	-2.774404	2.645465	-0.778994
13	6	0	-1.628008	2.215746	-1.318474
14	6	0	-0.680730	3.242467	-1.972638
15	7	0	0.555260	3.272678	-1.185295
16	1	0	1.264544	3.812079	-1.666990
17	1	0	0.939531	2.341146	-1.068321
18	1	0	-0.516971	2.800973	-2.969719
19	6	0	-1.210304	4.664199	-2.220341
20	6	0	-1.394999	5.599977	-1.001420
21	1	0	-0.683271	6.424335	-1.036588
22	1	0	-1.186281	5.044065	-0.085237
23	6	0	-2.829548	6.174763	-0.890874
24	8	0	-3.749917	5.305452	-0.906809
25	8	0	-2.961819	7.414779	-0.791621
26	1	0	-2.157956	4.578956	-2.755759
27	1	0	-0.509082	5.131440	-2.921394
28	8	0	-1.287602	1.025744	-1.342824
29	1	0	-3.059765	3.639447	-0.827251
30	1	0	-4.383684	2.355831	0.439758
31	6	0	-4.661540	1.145529	-1.307058
32	1	0	-5.095259	1.967820	-1.878190
33	1	0	-5.472821	0.545232	-0.895364
34	1	0	-4.077606	0.524803	-1.986399
35	8	0	-2.256957	1.083507	1.555776
36	1	0	-4.434204	-0.786994	0.232149
37	6	0	-4.072626	-2.710537	1.738829
38	6	0	-5.395674	-2.272976	2.388827
39	6	0	-6.544018	-1.813254	1.441031
40	8	0	-7.699959	-1.883493	1.916376
41	8	0	-6.211922	-1.403196	0.294656
42	1	0	-5.798756	-3.091564	2.988870
43	1	0	-5.217208	-1.450948	3.091039
44	1	0	-4.283526	-3.168222	0.769369
45	1	0	-3.593103	-3.472733	2.352506
46	1	0	-2.789139	-1.150181	2.511592
47	8	0	-1.121910	-3.042443	1.847757

48	1	0	-1.712574	-1.195694	-0.625659
49	6	0	-0.199086	-4.046850	-1.096604
50	6	0	0.594222	-4.491717	-2.330637
51	6	0	0.352705	-5.950705	-2.843864
52	8	0	1.033420	-6.266397	-3.856883
53	8	0	-0.477864	-6.660454	-2.229973
54	1	0	0.366989	-3.830931	-3.175863
55	1	0	1.664872	-4.384965	-2.151183
56	1	0	-1.256049	-4.252482	-1.266326
57	1	0	0.093501	-4.631023	-0.227644
58	1	0	0.011398	-2.009911	-1.736662
59	8	0	2.176279	-2.969683	0.080582
60	1	0	3.043813	-1.201709	1.455756
61	6	0	3.504046	0.248310	-0.086069
62	8	0	3.185516	1.240395	-0.730857
63	7	0	4.691861	-0.390716	-0.189988
64	6	0	5.876889	0.031398	-0.933891
65	1	0	6.522995	-0.851474	-0.947899
66	6	0	6.749885	1.118286	-0.204414
67	8	0	7.695900	1.571944	-0.869051
68	8	0	6.466444	1.398041	0.996447
69	6	0	5.577230	0.395820	-2.389975
70	1	0	5.068799	-0.430449	-2.890423
71	1	0	6.517479	0.607435	-2.894951
72	1	0	4.935897	1.271060	-2.445372
73	1	0	4.798863	-1.196564	0.400856
74	6	0	2.212758	0.711513	2.025302
75	6	0	3.434786	1.417938	2.645498
76	1	0	3.059599	2.193057	3.315453
77	1	0	4.025310	1.903201	1.869639
78	6	0	4.314818	0.509449	3.497398
79	8	0	3.942836	0.088564	4.577000
80	8	0	5.511605	0.195040	3.021392
81	1	0	5.781796	0.663822	2.150865
82	1	0	1.617113	0.242732	2.808388
83	1	0	1.592516	1.475566	1.554474

27. **Table S27:** Cartesian Coordinates for the low-energy geometry of the **EA+EEEA** hexapeptide calculated using the B3LYP method and the 6-311+G(*2df,2pd*) level of theory. Final Energy = -1550137 kcal/mol, Relative Energy = 24724 kcal/mol, Net Charge = -4, Multiplicity = 1.

Standard Orientation					
Center Number	Atomic Number	Atomic Type	Coordinates (Angstroms)		
			X	Y	Z
1	6	0	-2.197851	1.774344	-0.220429
2	6	0	-2.967658	3.120039	-0.145732
3	6	0	-2.634530	4.068983	-1.303902
4	6	0	-3.505991	5.360512	-1.442577
5	8	0	-3.248389	6.055937	-2.461777
6	8	0	-4.363283	5.587173	-0.557461
7	1	0	-1.591153	4.384520	-1.239364
8	1	0	-2.723959	3.534924	-2.257013
9	1	0	-4.034223	2.901413	-0.163213
10	1	0	-2.774200	3.606920	0.806655
11	7	0	-3.021346	0.574728	0.014234
12	1	0	-3.089036	-0.116561	-0.727269
13	6	0	-3.770456	0.331268	1.111214
14	6	0	-4.486749	-1.052181	1.204172
15	7	0	-5.319719	-1.548287	0.092106
16	6	0	-4.884488	-1.882855	-1.122941
17	6	0	-5.797546	-2.688420	-2.071931
18	7	0	-5.910392	-1.917163	-3.319588
19	1	0	-6.182226	-2.526512	-4.081698
20	1	0	-4.998955	-1.531001	-3.541262
21	1	0	-5.192909	-3.597751	-2.225514
22	6	0	-7.166736	-3.172482	-1.587587
23	6	0	-8.257181	-2.119744	-1.290552
24	1	0	-9.098239	-2.225400	-1.974968
25	1	0	-7.852441	-1.116514	-1.439988
26	6	0	-8.770535	-2.219086	0.168580
27	8	0	-7.856882	-2.190206	1.046213
28	8	0	-9.999180	-2.326006	0.353476
29	1	0	-7.003697	-3.778680	-0.694530
30	1	0	-7.532679	-3.862777	-2.357106
31	8	0	-3.750660	-1.627809	-1.561320
32	1	0	-6.290445	-1.797364	0.385569
33	1	0	-5.195360	-0.895274	2.012907
34	6	0	-3.469918	-2.115122	1.651112
35	1	0	-3.991989	-3.029237	1.938862
36	1	0	-2.905689	-1.751330	2.509871
37	1	0	-2.773645	-2.352182	0.847578
38	8	0	-3.837738	1.066807	2.084716
39	6	0	-0.851602	1.773039	0.558647
40	8	0	-0.194909	2.793638	0.672870
41	7	0	-0.428866	0.559429	1.011617
42	6	0	0.933255	0.250896	1.442946
43	6	0	1.328963	0.958569	2.764239
44	6	0	2.501909	0.345243	3.548899
45	6	0	2.438778	0.477255	5.118525
46	8	0	1.303924	0.489014	5.638578
47	8	0	3.561515	0.506762	5.682720

48	1	0	2.560417	-0.733513	3.358847
49	1	0	3.471986	0.764499	3.267475
50	1	0	0.458429	0.895123	3.417224
51	1	0	1.496476	2.017864	2.571818
52	6	0	1.894830	0.324005	0.209658
53	8	0	1.462605	0.194257	-0.920837
54	7	0	3.217954	0.457936	0.519264
55	6	0	4.396895	0.357651	-0.342386
56	1	0	5.151679	0.977802	0.149753
57	6	0	4.939691	-1.095849	-0.404365
58	8	0	4.298347	-2.003735	-0.905404
59	7	0	6.180738	-1.238453	0.138827
60	6	0	7.051868	-2.411698	0.043904
61	1	0	7.861272	-2.208475	0.752767
62	6	0	7.798745	-2.557426	-1.332675
63	8	0	8.357736	-3.645209	-1.532507
64	8	0	7.832061	-1.539533	-2.088448
65	6	0	6.368307	-3.702015	0.496781
66	1	0	5.959013	-3.580957	1.501694
67	1	0	7.103334	-4.504016	0.495719
68	1	0	5.554431	-3.965196	-0.172615
69	1	0	6.643379	-0.382004	0.389589
70	6	0	4.202382	0.911641	-1.761631
71	6	0	5.469146	0.822688	-2.636149
72	1	0	5.220436	1.242111	-3.612654
73	1	0	5.758348	-0.217087	-2.783514
74	6	0	6.650931	1.632225	-2.111837
75	8	0	6.631372	2.847534	-2.077063
76	8	0	7.710022	0.954467	-1.686222
77	1	0	7.664678	-0.066581	-1.825196
78	1	0	3.878112	1.949074	-1.682165
79	1	0	3.402763	0.357867	-2.243707
80	1	0	3.424760	0.525037	1.502506
81	1	0	0.914109	-0.820726	1.670711
82	1	0	-0.997900	-0.221511	0.737528
83	1	0	-1.862570	1.652560	-1.254714

28. **Table S28:** Cartesian Coordinates for the low-energy geometry of the **EGED+EA** hexapeptide calculated using the B3LYP method and the 6-311+G(*2df,2pd*) level of theory. Final Energy = -1476844 kcal/mol, Relative Energy = 98017 kcal/mol, Net Charge = -4, Multiplicity = 1.

**Standard Orientation**					
Center Number	Atomic Number	Atomic Type	Coordinates (Angstroms)		
			X	Y	Z
1	6	0	-2.697149	0.842304	0.106045
2	7	0	-3.933658	0.080246	0.033533
3	1	0	-4.755123	0.584948	-0.332130
4	6	0	-4.002351	-1.111177	0.643192
5	6	0	-5.317239	-1.926591	0.650356
6	7	0	-6.555887	-1.275647	0.248770
7	6	0	-7.050125	-1.189319	-1.007322
8	6	0	-8.524927	-0.731934	-1.098884
9	1	0	-8.579540	-0.280820	-2.089499
10	7	0	-9.372239	-1.953463	-1.139767
11	1	0	-10.336448	-1.650036	-1.239507
12	1	0	-9.335057	-2.373708	-0.214255
13	6	0	-9.014197	0.317381	-0.067773
14	1	0	-9.821477	0.890042	-0.527967
15	1	0	-8.203265	1.018334	0.145168
16	6	0	-9.565431	-0.287694	1.251278
17	8	0	-10.750115	-0.025558	1.554582
18	8	0	-8.766131	-1.026677	1.902237
19	8	0	-6.431561	-1.470209	-2.028485
20	1	0	-7.250291	-1.115399	0.997960
21	1	0	-5.451628	-2.137119	1.713546
22	6	0	-5.072265	-3.275504	-0.048190
23	1	0	-4.928253	-3.134191	-1.115881
24	1	0	-5.937735	-3.924535	0.099746
25	1	0	-4.189521	-3.744191	0.383131
26	8	0	-3.035268	-1.610460	1.228335
27	1	0	-2.433961	0.983422	1.160705
28	6	0	-2.851751	2.224128	-0.555047
29	6	0	-3.788510	3.220370	0.149167
30	6	0	-5.275208	3.221966	-0.315940
31	8	0	-5.866158	4.322289	-0.238605
32	8	0	-5.738626	2.119844	-0.710205
33	1	0	-3.789568	3.035364	1.229499
34	1	0	-3.408990	4.235275	0.018121
35	1	0	-3.177835	2.071714	-1.585070
36	1	0	-1.848319	2.655755	-0.598822
37	6	0	-1.506438	0.122915	-0.570346
38	8	0	-1.583688	-0.407713	-1.667967
39	7	0	-0.327171	0.242899	0.096775
40	1	0	-0.297417	0.561142	1.053290
41	6	0	0.913618	-0.297253	-0.404194
42	6	0	2.071274	0.092722	0.527479
43	8	0	1.848973	0.589684	1.631439
44	7	0	3.300540	-0.163800	0.049115
45	6	0	4.542590	-0.028305	0.813237
46	6	0	5.610084	-0.957526	0.171994
47	7	0	6.629250	-1.210232	0.924620

48	6	0	7.808834	-1.980264	0.494443
49	6	0	7.598707	-3.342223	-0.201195
50	8	0	8.293919	-4.303858	0.089716
51	8	0	6.677134	-3.442714	-1.147639
52	1	0	6.130731	-2.566348	-1.208054
53	1	0	8.332491	-2.250379	1.411908
54	6	0	8.836258	-1.137155	-0.318024
55	1	0	9.565854	-1.816886	-0.761142
56	1	0	8.308034	-0.625074	-1.157998
57	6	0	9.625204	-0.112688	0.493459
58	8	0	10.494523	0.571117	-0.394405
59	1	0	9.935358	1.016260	-1.084006
60	8	0	8.685510	0.916188	0.944048
61	1	0	7.912089	0.431644	1.271127
62	8	0	5.333554	-1.352898	-1.026877
63	1	0	4.357638	-0.374046	1.828852
64	6	0	4.999846	1.440461	0.911275
65	1	0	4.147149	2.028050	1.254423
66	1	0	5.766936	1.502968	1.681370
67	6	0	5.558410	2.005846	-0.400519
68	1	0	4.958351	1.715715	-1.261394
69	1	0	6.568494	1.600664	-0.562033
70	6	0	5.712172	3.493539	-0.454390
71	8	0	5.656100	4.183327	-1.450512
72	8	0	5.978114	4.061370	0.753792
73	1	0	6.124571	4.999049	0.570042
74	1	0	3.438624	-0.664370	-0.822656
75	1	0	0.870926	-1.390357	-0.469160
76	1	0	1.102654	0.063239	-1.416869

29. **Table S29:** Cartesian Coordinates for the low-energy geometry of the **EG+EDEA** hexapeptide calculated using the B3LYP method and the 6-311+G(*2df,2pd*) level of theory. Final Energy = -1476154 kcal/mol, Relative Energy = 98707 kcal/mol, Net Charge = -4, Multiplicity = 1.

Standard Orientation					
Center Number	Atomic Number	Atomic Type	Coordinates (Angstroms)		
			X	Y	Z
1	6	0	-1.614630	1.860259	-0.634929
2	7	0	-2.856774	1.840114	0.118708
3	1	0	-3.197298	2.796183	0.304915
4	6	0	-3.748117	0.835701	0.064703
5	6	0	-5.044768	1.141752	0.827920
6	7	0	-6.080815	0.177554	0.525411
7	6	0	-7.230455	0.114529	1.218705
8	6	0	-8.224906	-0.997223	0.826441
9	7	0	-7.676992	-1.938325	-0.149098
10	1	0	-8.457999	-2.413778	-0.610823
11	1	0	-7.096236	-2.635656	0.299752
12	1	0	-8.459528	-1.473192	1.790006
13	6	0	-9.538691	-0.377822	0.303927
14	6	0	-10.824546	-1.169121	0.591945
15	6	0	-11.161903	-2.447645	-0.237342
16	8	0	-12.296410	-2.932015	-0.007056
17	8	0	-10.297511	-2.871870	-1.045803
18	1	0	-10.846153	-1.461352	1.647238
19	1	0	-11.682499	-0.501605	0.464997
20	1	0	-9.430532	-0.221714	-0.772647
21	1	0	-9.637849	0.603654	0.770922
22	8	0	-7.500654	0.883886	2.144674
23	1	0	-5.909169	-0.508126	-0.198517
24	1	0	-5.375250	2.157119	0.595758
25	1	0	-4.830040	1.128007	1.898940
26	8	0	-3.579430	-0.240477	-0.502723
27	1	0	-1.799875	1.450995	-1.632579
28	6	0	-1.116813	3.325465	-0.754610
29	6	0	-2.186477	4.405495	-1.108947
30	8	0	-3.179871	4.464489	-0.324372
31	8	0	-1.950734	5.138844	-2.088666
32	1	0	-0.696447	3.608570	0.214113
33	1	0	-0.317532	3.359352	-1.491212
34	6	0	-0.479978	1.039932	0.036081
35	8	0	-0.342208	0.989973	1.249074
36	7	0	0.387357	0.487796	-0.846734
37	1	0	0.182627	0.521938	-1.833447
38	6	0	1.660720	-0.163698	-0.520188
39	1	0	2.057039	0.287439	0.385733
40	6	0	2.560093	0.106652	-1.740819
41	8	0	2.065800	0.057664	-2.870098
42	7	0	3.869607	0.421621	-1.597854
43	6	0	4.771294	0.430313	-0.446311
44	1	0	4.239163	0.029237	0.410602
45	6	0	5.965427	-0.485195	-0.786570
46	8	0	6.693822	-0.206056	-1.743719
47	7	0	6.131400	-1.509367	0.057469

48	6	0	7.259214	-2.433934	0.034125
49	1	0	7.057027	-3.130341	0.852571
50	6	0	8.631615	-1.778112	0.428827
51	8	0	9.651227	-2.417056	0.117379
52	8	0	8.601107	-0.697786	1.090650
53	6	0	7.332416	-3.249486	-1.260953
54	1	0	6.389826	-3.776575	-1.419506
55	1	0	8.148754	-3.966285	-1.190950
56	1	0	7.515022	-2.594054	-2.110384
57	1	0	5.309256	-1.752318	0.624214
58	6	0	5.287210	1.864015	-0.185749
59	6	0	6.577040	1.986005	0.650172
60	1	0	6.895790	3.029858	0.599337
61	1	0	7.363746	1.375526	0.211001
62	6	0	6.433394	1.685218	2.139967
63	8	0	5.820041	2.438317	2.875885
64	8	0	7.034720	0.606625	2.602820
65	1	0	7.567946	0.063214	1.910895
66	1	0	4.486737	2.444814	0.275446
67	1	0	5.496724	2.313799	-1.158599
68	1	0	4.344257	0.509321	-2.487309
69	6	0	1.474521	-1.681097	-0.288624
70	6	0	2.690560	-2.492061	0.243161
71	8	0	2.746761	-3.687383	-0.103235
72	8	0	3.487882	-1.876544	1.010279
73	1	0	0.680813	-1.780303	0.455169
74	1	0	1.115524	-2.143998	-1.207231

30. **Table S30:** Cartesian Coordinates for the low-energy geometry of the **EAQG+PK** hexapeptide calculated using the B3LYP method and the 6-311+G(*2df,2pd*) level of theory. Electronic Energy = -1385855 kcal/mol, Relative Energy = 189006 kcal/mol, Net Charge = 0, Multiplicity = 1.

**Standard Orientation**					
Center Number	Atomic Number	Atomic Type	Coordinates (Angstroms)		
			X	Y	Z
1	6	0	-3.263688	-2.177616	-1.651357
2	6	0	-3.648617	-3.641872	-1.360900
3	6	0	-2.864984	-3.983572	-0.088109
4	6	0	-1.534185	-3.255359	-0.280135
5	7	0	-1.918001	-2.058347	-1.063533
6	6	0	-1.121002	-1.016998	-1.385202
7	6	0	0.325917	-1.049189	-0.908368
8	7	0	1.142049	-1.817421	-1.849140
9	6	0	2.501451	-1.795591	-1.995308
10	6	0	3.289658	-0.565463	-1.518708
11	7	0	4.521372	-1.025652	-0.888628
12	6	0	4.838968	-0.986181	0.449229
13	6	0	4.319117	0.221447	1.251488
14	7	0	3.310459	-0.113256	2.256743
15	6	0	2.404982	-1.105992	2.133231
16	6	0	1.335613	-1.175055	3.240839
17	7	0	0.320580	-2.144200	2.813898
18	1	0	-0.231374	-2.439562	3.609379
19	1	0	0.788860	-2.956214	2.428525
20	1	0	1.888453	-1.532176	4.122062
21	6	0	0.674761	0.156830	3.653486
22	6	0	-0.044927	0.939777	2.551908
23	1	0	-0.844190	1.533685	3.003054
24	1	0	-0.545314	0.261612	1.862442
25	6	0	0.804783	1.943823	1.746002
26	8	0	1.897271	2.328484	2.212864
27	8	0	0.302319	2.328989	0.643864
28	1	0	1.412126	0.804690	4.129128
29	1	0	-0.043477	-0.098434	4.437217
30	8	0	2.446765	-1.960781	1.252773
31	1	0	2.986737	0.714294	2.751316
32	1	0	3.869388	0.934421	0.562016
33	6	0	5.501402	0.903915	1.940500
34	1	0	5.154288	1.765190	2.511950
35	1	0	6.226438	1.248901	1.202573
36	1	0	5.998578	0.201931	2.605815
37	8	0	5.639860	-1.761261	0.931956
38	1	0	4.911990	-1.841523	-1.346172
39	1	0	2.709818	-0.015107	-0.787623
40	6	0	3.566761	0.324085	-2.756658
41	6	0	3.889795	1.792623	-2.428068
42	6	0	2.646164	2.493261	-1.911021
43	7	0	2.659259	3.008479	-0.682104
44	1	0	3.467663	2.895255	-0.095347
45	1	0	1.764054	3.094366	-0.172318
46	8	0	1.630913	2.525417	-2.630812
47	1	0	4.713766	1.862797	-1.717411

48	1	0	4.199219	2.295407	-3.345512
49	1	0	2.697671	0.313210	-3.414693
50	1	0	4.392668	-0.121909	-3.309905
51	8	0	3.081237	-2.696976	-2.576878
52	1	0	0.757328	-2.692460	-2.175537
53	1	0	0.401359	-1.458339	0.098692
54	1	0	0.654484	-0.015645	-0.879669
55	8	0	-1.556555	-0.069057	-2.048195
56	1	0	-0.833138	-3.874373	-0.845564
57	1	0	-1.061908	-2.966089	0.656367
58	1	0	-3.379121	-3.597400	0.793010
59	1	0	-2.726478	-5.053813	0.051385
60	1	0	-3.319169	-4.271159	-2.188582
61	1	0	-4.724362	-3.763844	-1.261476
62	1	0	-3.214454	-1.974102	-2.718978
63	6	0	-4.317554	-1.188135	-1.129522
64	8	0	-5.320720	-0.984326	-1.794141
65	7	0	-4.095817	-0.608124	0.071714
66	1	0	-3.252950	-0.803574	0.587807
67	6	0	-4.965065	0.429874	0.592685
68	6	0	-4.784133	1.781821	-0.143253
69	1	0	-5.099946	1.609067	-1.172650
70	1	0	-5.476024	2.506707	0.287702
71	6	0	-3.346251	2.298675	-0.117871
72	1	0	-3.059642	2.607096	0.890175
73	1	0	-2.682484	1.475140	-0.375640
74	6	0	-3.094314	3.462303	-1.083328
75	1	0	-3.591107	3.270752	-2.040779
76	6	0	-1.609678	3.724401	-1.320136
77	1	0	-1.046399	3.697499	-0.389952
78	1	0	-1.448202	4.682952	-1.811252
79	7	0	-0.991833	2.680861	-2.200595
80	1	0	-1.344966	2.750039	-3.152335
81	1	0	0.064583	2.751126	-2.253800
82	1	0	-1.188390	1.709138	-1.901665
83	1	0	-3.540697	4.379509	-0.693268
84	1	0	-6.001878	0.117749	0.456005
85	6	0	-4.711822	0.548718	2.083713
86	8	0	-3.839110	-0.020446	2.687091
87	8	0	-5.586225	1.387944	2.670460
88	1	0	-5.365222	1.429732	3.612351

31. **Table S31:** Cartesian Coordinates for the low-energy geometry of the **EA+QGPK** hexapeptide calculated using the B3LYP method and the 6-311+G(*2df,2pd*) level of theory. Final Energy = -1385855 kcal/mol, Relative Energy = 189006 kcal/mol, Net Charge = 0, Multiplicity = 1.

Standard Orientation					
Center Number	Atomic Number	Atomic Type	Coordinates (Angstroms)		
			X	Y	Z
1	6	0	5.764620	-0.582945	-0.543221
2	7	0	5.804794	-0.876775	-1.971979
3	1	0	6.672601	-1.328538	-2.228128
4	1	0	5.723838	-0.011750	-2.493197
5	1	0	6.720557	-0.168853	-0.180931
6	6	0	4.817630	0.612627	-0.331047
7	8	0	4.718369	1.461155	-1.220648
8	7	0	4.232103	0.728836	0.876811
9	6	0	3.485774	1.900054	1.357050
10	1	0	3.308578	1.703494	2.412184
11	6	0	2.063417	2.001399	0.772886
12	8	0	1.097415	2.041593	1.528941
13	7	0	1.968499	2.004974	-0.573150
14	6	0	0.697214	1.878211	-1.269105
15	1	0	0.923991	1.845840	-2.336826
16	6	0	0.066389	0.521635	-0.876546
17	6	0	0.675870	-0.669058	-1.631857
18	6	0	-0.095426	-1.011314	-2.892059
19	7	0	-0.293336	-2.331794	-3.102164
20	1	0	-0.299830	-2.949381	-2.298010
21	1	0	-0.846540	-2.591902	-3.902588
22	8	0	-0.499242	-0.144665	-3.668726
23	1	0	1.704038	-0.448033	-1.930479
24	1	0	0.718897	-1.541803	-0.987452
25	1	0	-1.006057	0.509826	-1.017980
26	1	0	0.224064	0.389073	0.187297
27	6	0	-0.135903	3.173017	-1.092585
28	8	0	0.337275	4.157405	-0.561844
29	7	0	-1.385284	3.262969	-1.666309
30	1	0	-1.812818	4.146379	-1.433565
31	6	0	-2.272834	2.242482	-2.162545
32	1	0	-2.959905	2.702129	-2.879306
33	1	0	-1.724613	1.485167	-2.720102
34	6	0	-3.120049	1.615837	-1.044857
35	8	0	-3.174589	2.154350	0.066923
36	7	0	-3.801886	0.489001	-1.314559
37	6	0	-4.727608	-0.081279	-0.315052
38	6	0	-5.561191	-1.086527	-1.138607
39	6	0	-4.670660	-1.448206	-2.336215
40	6	0	-3.928309	-0.147999	-2.642666
41	1	0	-4.517671	0.491768	-3.305806
42	1	0	-2.953049	-0.305193	-3.098333
43	1	0	-3.961847	-2.233449	-2.072326
44	1	0	-5.243441	-1.800461	-3.191862
45	1	0	-6.471579	-0.594006	-1.482224
46	1	0	-5.857583	-1.951932	-0.550111
47	1	0	-5.345400	0.713985	0.095632

48	6	0	-4.045587	-0.706428	0.927858
49	8	0	-4.433329	-0.345851	2.050041
50	7	0	-3.105783	-1.619266	0.699854
51	1	0	-2.802499	-1.828014	-0.248358
52	6	0	-2.324238	-2.433939	1.643779
53	6	0	-1.908159	-1.724077	2.932468
54	1	0	-2.807897	-1.487572	3.498289
55	1	0	-1.334635	-2.446755	3.511191
56	6	0	-1.073576	-0.458956	2.705222
57	1	0	-0.037538	-0.734248	2.519856
58	1	0	-1.408106	0.027227	1.787419
59	6	0	-1.148656	0.555816	3.866002
60	1	0	-1.833025	0.199239	4.641593
61	6	0	-1.574681	1.946886	3.422573
62	1	0	-0.875055	2.348174	2.693036
63	1	0	-1.635021	2.630807	4.266873
64	7	0	-2.948667	1.928583	2.767128
65	1	0	-3.558832	2.636431	3.164428
66	1	0	-2.911213	2.100764	1.741607
67	1	0	-3.442902	1.018891	2.831711
68	1	0	-0.176114	0.667098	4.345356
69	1	0	-2.926689	-3.305579	1.920410
70	6	0	-1.111289	-2.985637	0.821889
71	8	0	-1.243680	-2.981245	-0.423407
72	8	0	-0.130817	-3.373214	1.494326
73	1	0	2.835541	1.995830	-1.098825
74	6	0	4.264080	3.213374	1.242338
75	1	0	5.212665	3.128174	1.773005
76	1	0	3.682979	4.013380	1.700156
77	1	0	4.467584	3.479456	0.209399
78	1	0	4.047155	-0.135116	1.380716
79	6	0	5.541419	-1.874133	0.270843
80	6	0	4.343897	-2.741674	-0.130058
81	1	0	4.611595	-3.798967	-0.110406
82	1	0	4.066128	-2.540361	-1.166745
83	6	0	3.096610	-2.601464	0.721784
84	8	0	2.962558	-1.761145	1.598818
85	8	0	2.187675	-3.490488	0.389072
86	1	0	1.292774	-3.413847	0.880228
87	1	0	5.509841	-1.646459	1.337014
88	1	0	6.448362	-2.465965	0.129131

32. **Table S32:** Cartesian Coordinates for the low-energy geometry of the **AEEE+VA** hexapeptide calculated using the B3LYP method and the 6-311+G(*2df,2pd*) level of theory. Final Energy = -1456889 kcal/mol, Relative Energy = 117972 kcal/mol, Net Charge = -3, Multiplicity = 1.

**Standard Orientation**					
Center Number	Atomic Number	Atomic Type	Coordinates (Angstroms)		
			X	Y	Z
1	6	0	3.925358	-1.821712	-0.081309
2	7	0	3.363588	-0.571002	0.405847
3	1	0	3.045178	-0.515263	1.365998
4	6	0	3.259188	0.564869	-0.338370
5	6	0	2.736267	1.777873	0.474836
6	7	0	1.337941	1.553394	0.876831
7	6	0	1.021842	0.920826	2.023351
8	6	0	-0.450747	0.934649	2.471358
9	7	0	-1.410043	1.010591	1.377116
10	6	0	-2.555690	1.748314	1.498661
11	6	0	-3.730353	1.412657	0.555613
12	7	0	-3.682409	0.054269	0.014740
13	6	0	-4.335560	-0.990597	0.586152
14	6	0	-4.392381	-2.295585	-0.229236
15	7	0	-4.328509	-3.475880	0.632716
16	1	0	-4.856683	-3.271002	1.475785
17	1	0	-3.366863	-3.622852	0.929958
18	1	0	-3.541471	-2.340215	-0.909733
19	6	0	-5.692673	-2.304763	-1.044534
20	1	0	-6.555215	-2.305145	-0.374908
21	1	0	-5.726854	-3.208666	-1.652527
22	1	0	-5.771135	-1.433365	-1.696278
23	8	0	-4.872597	-0.913466	1.687171
24	1	0	-3.338885	-0.069275	-0.924669
25	6	0	-3.936309	2.460708	-0.554291
26	1	0	-3.090538	2.433356	-1.250145
27	6	0	-5.256067	2.282124	-1.309433
28	1	0	-5.227397	1.418373	-1.975843
29	1	0	-6.055435	2.077774	-0.587668
30	6	0	-5.749054	3.511471	-2.150276
31	8	0	-6.595840	3.228092	-3.033054
32	8	0	-5.292239	4.632040	-1.835458
33	1	0	-3.924047	3.450730	-0.101405
34	1	0	-4.594946	1.445431	1.217496
35	8	0	-2.683292	2.641257	2.323291
36	1	0	-1.430422	0.190004	0.787479
37	6	0	-0.753341	-0.207010	3.477697
38	6	0	-1.443758	-1.474163	2.954255
39	6	0	-0.830371	-2.186819	1.746920
40	8	0	-1.185112	-3.402963	1.588250
41	8	0	-0.087485	-1.545112	0.976422
42	1	0	-1.511040	-2.199894	3.766851
43	1	0	-2.480396	-1.254460	2.686226
44	1	0	0.193539	-0.462673	3.950358
45	1	0	-1.399659	0.206437	4.255380
46	1	0	-0.557041	1.878896	3.010116
47	8	0	1.887055	0.435261	2.757623

48	1	0	0.594064	1.915491	0.301942
49	6	0	2.957791	3.123849	-0.215341
50	6	0	4.438302	3.519636	-0.271620
51	6	0	4.746254	5.044508	-0.467204
52	8	0	5.943511	5.296439	-0.753398
53	8	0	3.803705	5.847542	-0.287104
54	1	0	4.923101	3.248352	0.674645
55	1	0	4.962747	2.965531	-1.048825
56	1	0	2.424230	3.896267	0.340272
57	1	0	2.536421	3.102609	-1.222466
58	1	0	3.297430	1.780630	1.410276
59	8	0	3.566246	0.614256	-1.517164
60	1	0	3.746594	-2.538223	0.727204
61	6	0	3.157774	-2.402300	-1.302236
62	8	0	3.745314	-2.960432	-2.225876
63	7	0	1.818611	-2.323966	-1.224062
64	1	0	1.365962	-1.928857	-0.403117
65	6	0	0.957283	-2.960718	-2.214515
66	6	0	0.676974	-2.056018	-3.409610
67	1	0	0.145649	-1.158665	-3.097469
68	1	0	0.057067	-2.574574	-4.141998
69	1	0	1.617609	-1.764659	-3.874446
70	6	0	-0.320832	-3.421848	-1.497238
71	8	0	-0.067415	-4.215735	-0.477611
72	1	0	-0.635797	-3.941746	0.378561
73	8	0	-1.427081	-3.064629	-1.852921
74	1	0	1.482496	-3.856311	-2.556538
75	6	0	5.457335	-1.784574	-0.335079
76	6	0	6.188615	-0.870249	0.651319
77	1	0	7.266054	-0.924749	0.476881
78	1	0	6.005360	-1.171353	1.687221
79	1	0	5.890322	0.170357	0.548311
80	6	0	6.045356	-3.200337	-0.266881
81	1	0	5.937749	-3.614550	0.741665
82	1	0	7.113275	-3.181438	-0.500760
83	1	0	5.551719	-3.864475	-0.972074
84	1	0	5.607628	-1.398828	-1.343357

33. **Table S33:** Cartesian Coordinates for the low-energy geometry of the **AE+EEVA** hexapeptide calculated using the B3LYP method and the 6-311+G(*2df,2pd*) level of theory. Final Energy = -1456952 kcal/mol, Relative Energy = 117909 kcal/mol, Net Charge = -3, Multiplicity = 1.

**Standard Orientation**					
**Center Number**	**Atomic Number**	Atomic Type	Coordinates (Angstroms)		
			X	Y	Z
1	6	0	-1.822220	2.245320	-0.976400
2	6	0	-2.005476	3.756150	-0.731984
3	6	0	-3.306959	4.366185	-1.277988
4	6	0	-4.528540	4.393071	-0.308543
5	8	0	-5.358745	5.304084	-0.506246
6	8	0	-4.572367	3.490514	0.574954
7	1	0	-3.128851	5.397180	-1.588096
8	1	0	-3.625466	3.831429	-2.179844
9	1	0	-1.950185	3.942480	0.343406
10	1	0	-1.143812	4.243978	-1.186154
11	7	0	-2.917607	1.505821	-0.385604
12	1	0	-3.586020	2.080919	0.158310
13	6	0	-3.233825	0.253084	-0.780848
14	6	0	-4.472884	-0.348423	-0.085572
15	7	0	-4.165993	-1.654801	0.478486
16	6	0	-3.155944	-1.875898	1.318819
17	6	0	-3.023590	-3.323191	1.827059
18	7	0	-1.662817	-3.846395	1.646733
19	1	0	-1.006197	-3.159807	2.008571
20	1	0	-1.457394	-3.909439	0.655405
21	1	0	-3.705910	-3.958770	1.261630
22	6	0	-3.402821	-3.379893	3.307276
23	1	0	-2.741382	-2.734320	3.887712
24	1	0	-3.305883	-4.402343	3.675193
25	1	0	-4.429561	-3.043608	3.458860
26	8	0	-2.347717	-1.017027	1.699690
27	1	0	-4.832866	-2.413474	0.210036
28	6	0	-5.627940	-0.485516	-1.091522
29	1	0	-5.289491	-1.121996	-1.911229
30	6	0	-6.963053	-1.012091	-0.527633
31	1	0	-7.105914	-0.636192	0.491619
32	1	0	-7.786507	-0.598994	-1.110858
33	6	0	-7.186653	-2.553628	-0.478961
34	8	0	-8.366276	-2.938374	-0.606793
35	8	0	-6.167704	-3.283817	-0.291926
36	1	0	-5.796273	0.510793	-1.506507
37	1	0	-4.775784	0.332411	0.715051
38	8	0	-2.598921	-0.376169	-1.621434
39	6	0	-0.415992	1.842346	-0.480301
40	7	0	-0.337123	0.952614	0.521291
41	6	0	0.869737	0.572711	1.279711
42	6	0	1.510486	1.789633	1.980964
43	6	0	2.579665	1.446530	3.029526
44	6	0	4.061169	1.673278	2.626405
45	8	0	4.301443	1.969858	1.422520
46	8	0	4.901597	1.527855	3.542657
47	1	0	2.416335	2.033231	3.936107

48	1	0	2.504354	0.401958	3.343872
49	1	0	0.682391	2.320257	2.457215
50	1	0	1.942749	2.457631	1.239524
51	6	0	1.870658	-0.296730	0.478640
52	8	0	2.180600	-1.416630	0.870926
53	7	0	2.371391	0.263574	-0.642471
54	6	0	3.208367	-0.432254	-1.605215
55	1	0	3.521360	0.348244	-2.307306
56	6	0	4.536356	-0.959993	-1.006477
57	8	0	5.024453	-2.028438	-1.374223
58	7	0	5.204444	-0.139570	-0.168914
59	1	0	4.764582	0.673416	0.293037
60	6	0	6.470941	-0.599469	0.365619
61	6	0	7.123853	0.462490	1.261666
62	1	0	7.317989	1.374045	0.695871
63	1	0	8.067274	0.072471	1.647708
64	1	0	6.468897	0.726723	2.092669
65	6	0	7.470610	-0.982080	-0.716030
66	8	0	7.419228	-0.197106	-1.820828
67	1	0	8.104576	-0.539070	-2.409708
68	8	0	8.329372	-1.825331	-0.591276
69	1	0	6.327884	-1.517570	0.940135
70	6	0	2.477543	-1.524611	-2.437384
71	6	0	1.016950	-1.161853	-2.715993
72	1	0	0.560795	-1.922556	-3.353241
73	1	0	0.933674	-0.204305	-3.236743
74	1	0	0.413431	-1.092972	-1.815169
75	6	0	3.209760	-1.767491	-3.765114
76	1	0	3.176609	-0.868005	-4.389790
77	1	0	2.721639	-2.569552	-4.325024
78	1	0	4.248630	-2.044115	-3.602481
79	1	0	2.506770	-2.446972	-1.856505
80	1	0	1.979565	1.167041	-0.901232
81	1	0	0.501784	-0.108176	2.043903
82	1	0	-1.195970	0.505153	0.828391
83	8	0	0.566557	2.373308	-1.014991
84	1	0	-1.797247	2.056294	-2.053515

34. **Table S34:** Cartesian Coordinates for the low-energy geometry of the **EEEI+DG** hexapeptide calculated using the B3LYP method and the 6-311+G(*2df,2pd*) level of theory. Final Energy = -1574798 kcal/mol, Relative Energy = 63 kcal/mol, Net Charge = -4, Multiplicity = 1.

**Standard Orientation**					
Center Number	Atomic Number	Atomic Type	Coordinates (Angstroms)		
			X	Y	Z
1	6	0	-4.725172	-0.843764	-1.883601
2	1	0	-5.397045	-1.004642	-2.724440
3	7	0	-4.419480	0.600763	-1.987217
4	1	0	-4.777841	1.022959	-2.829487
5	6	0	-3.673769	1.507724	-1.292455
6	6	0	-3.056892	1.120444	0.064852
7	7	0	-1.586569	1.191550	0.002940
8	6	0	-0.738587	2.135776	0.471341
9	6	0	0.754774	1.709649	0.530330
10	7	0	1.017801	1.590912	1.977262
11	6	0	1.641994	0.667451	2.742757
12	6	0	2.137384	-0.661803	2.134389
13	7	0	3.149403	-0.382208	1.102056
14	6	0	3.561081	-1.058419	0.021411
15	6	0	4.883278	-0.516558	-0.580703
16	6	0	5.969977	-1.600609	-0.423371
17	6	0	7.246231	-1.388170	-1.267065
18	6	0	8.522789	-0.933524	-0.493994
19	8	0	9.617538	-1.369063	-0.936206
20	8	0	8.338851	-0.158164	0.476071
21	1	0	7.496407	-2.302139	-1.808704
22	1	0	7.063098	-0.622559	-2.028444
23	1	0	6.247675	-1.642511	0.633152
24	1	0	5.513537	-2.555805	-0.684058
25	7	0	5.287779	0.746543	0.022523
26	1	0	4.998696	1.548977	-0.538960
27	1	0	6.290506	0.760357	0.192424
28	1	0	4.664373	-0.404448	-1.648986
29	8	0	3.012376	-2.041045	-0.500405
30	1	0	3.825005	0.362151	1.299250
31	6	0	0.981591	-1.593568	1.727992
32	6	0	0.013002	-1.914303	2.871540
33	6	0	-1.190626	-2.796461	2.422416
34	8	0	-1.447136	-3.800681	3.122219
35	8	0	-1.808678	-2.397457	1.395441
36	1	0	0.530892	-2.420352	3.688163
37	1	0	-0.404435	-0.988895	3.279363
38	1	0	1.418811	-2.519166	1.358300
39	1	0	0.419158	-1.164243	0.907012
40	1	0	2.656667	-1.127050	2.976073
41	8	0	1.805144	0.866744	3.948922
42	1	0	0.744192	2.412560	2.499276
43	1	0	0.898436	0.742353	0.061094
44	6	0	1.718078	2.738487	-0.082921
45	1	0	1.492703	3.714933	0.354945
46	1	0	2.723266	2.466233	0.236802
47	6	0	1.731619	2.840722	-1.609752

48	1	0	1.721110	1.833660	-2.039489
49	1	0	0.853086	3.365623	-1.984062
50	6	0	3.023472	3.544649	-2.140934
51	8	0	2.863387	4.469637	-2.972046
52	8	0	4.112501	3.096880	-1.692576
53	8	0	-1.054730	3.223334	0.944676
54	1	0	-1.156632	0.365809	-0.399453
55	6	0	-3.690173	1.963951	1.205023
56	6	0	-3.232676	1.429366	2.573957
57	6	0	-3.473550	2.403789	3.729192
58	1	0	-3.093025	1.992496	4.667294
59	1	0	-4.534877	2.621233	3.871592
60	1	0	-2.956286	3.348080	3.545053
61	1	0	-2.169153	1.199794	2.539177
62	1	0	-3.738745	0.480757	2.776257
63	6	0	-5.221614	2.007051	1.108380
64	1	0	-5.552528	2.435338	0.163050
65	1	0	-5.685368	1.025026	1.207006
66	1	0	-5.623603	2.638370	1.903734
67	1	0	-3.311004	2.975911	1.085139
68	1	0	-3.270914	0.073253	0.272282
69	8	0	-3.541897	2.646424	-1.727726
70	6	0	-3.495730	-1.700552	-2.260632
71	8	0	-3.240024	-1.881763	-3.451944
72	7	0	-2.760822	-2.253920	-1.273220
73	1	0	-2.929950	-2.100416	-0.283722
74	6	0	-1.591560	-3.047405	-1.545814
75	1	0	-1.537991	-3.862698	-0.827691
76	1	0	-1.662323	-3.451784	-2.556080
77	6	0	-0.290603	-2.265451	-1.441258
78	8	0	0.759680	-3.075015	-1.390992
79	1	0	1.584632	-2.577842	-1.122431
80	8	0	-0.218062	-1.056892	-1.410847
81	6	0	-5.557850	-1.269423	-0.673971
82	6	0	-7.087962	-0.927146	-0.810508
83	8	0	-7.714032	-0.858971	0.271494
84	8	0	-7.536509	-0.809410	-1.977173
85	1	0	-5.188540	-0.885376	0.273010
86	1	0	-5.507086	-2.359293	-0.594726

35. **Table S35:** Cartesian Coordinates for the low-energy geometry of the **EE+EIDG** hexapeptide calculated using the B3LYP method and the 6-311+G(*2df,2pd*) level of theory. Final Energy = -1574861 kcal/mol, Relative Energy = 0 kcal/mol, Net Charge = -4, Multiplicity = 1.

Standard Orientation					
Center Number	Atomic Number	Atomic Type	Coordinates (Angstroms)		
			X	Y	Z
1	6	0	0.828394	0.685469	0.882502
2	7	0	2.089496	0.876074	0.177631
3	1	0	1.975100	1.090131	-0.803237
4	6	0	3.320741	0.698983	0.715604
5	6	0	4.500644	0.960905	-0.261236
6	7	0	5.396381	-0.202611	-0.287483
7	6	0	6.496610	-0.487896	0.444865
8	6	0	7.129774	-1.870942	0.120377
9	6	0	8.512405	-1.678678	-0.532156
10	6	0	9.531868	-2.811408	-0.319135
11	6	0	9.456880	-4.109040	-1.182846
12	8	0	10.452428	-4.866686	-1.076920
13	8	0	8.434260	-4.288521	-1.893328
14	1	0	10.538677	-2.412808	-0.474237
15	1	0	9.511157	-3.130076	0.728951
16	1	0	8.363541	-1.511470	-1.602553
17	1	0	8.931924	-0.762145	-0.114427
18	7	0	6.276326	-2.719928	-0.717998
19	1	0	5.628147	-3.252284	-0.151465
20	1	0	6.869487	-3.374877	-1.237894
21	1	0	7.298110	-2.305161	1.114664
22	8	0	7.019622	0.223324	1.292947
23	1	0	5.151870	-0.962768	-0.910624
24	6	0	5.222774	2.302973	-0.015853
25	6	0	4.387614	3.543780	-0.350988
26	6	0	5.222868	4.850751	-0.588556
27	8	0	4.730669	5.908726	-0.121264
28	8	0	6.274961	4.724757	-1.261448
29	1	0	3.840260	3.373740	-1.287274
30	1	0	3.639755	3.745571	0.415855
31	1	0	6.109392	2.318877	-0.650017
32	1	0	5.572773	2.337285	1.014102
33	1	0	4.083165	1.010921	-1.271149
34	8	0	3.498455	0.353235	1.875693
35	6	0	0.579557	1.763085	1.985820
36	1	0	1.453129	2.413865	1.977041
37	1	0	-0.274337	2.383185	1.705420
38	6	0	0.383325	1.247730	3.419102
39	1	0	1.121660	0.467508	3.622426
40	1	0	0.616250	2.054214	4.117404
41	6	0	-1.006152	0.700368	3.837029
42	8	0	-1.805732	0.343447	2.917129
43	8	0	-1.222333	0.642338	5.062942
44	6	0	-0.275763	0.671670	-0.215109
45	8	0	0.021980	0.887744	-1.390927
46	7	0	-1.511362	0.451946	0.265764
47	1	0	-1.593873	0.376687	1.311422

48	6	0	-2.787146	0.500917	-0.452080
49	6	0	-2.849548	1.523977	-1.618269
50	6	0	-2.550968	2.944004	-1.109669
51	6	0	-2.229114	3.946893	-2.219605
52	1	0	-1.988910	4.927271	-1.801789
53	1	0	-3.063226	4.080566	-2.913163
54	1	0	-1.362825	3.613483	-2.793554
55	1	0	-1.702613	2.909350	-0.428495
56	1	0	-3.404451	3.299995	-0.521625
57	6	0	-4.211425	1.468944	-2.324169
58	1	0	-4.399591	0.484264	-2.752436
59	1	0	-5.025145	1.705606	-1.635128
60	1	0	-4.244868	2.188684	-3.144829
61	1	0	-2.073694	1.240112	-2.326094
62	1	0	-3.513032	0.821077	0.295858
63	6	0	-3.183011	-0.902491	-0.968174
64	8	0	-2.554245	-1.437961	-1.871567
65	7	0	-4.268567	-1.532197	-0.427933
66	6	0	-5.201535	-1.138031	0.627147
67	1	0	-4.688854	-0.483198	1.327203
68	6	0	-6.370261	-0.293722	0.069216
69	8	0	-6.522803	0.887988	0.368778
70	7	0	-7.180078	-0.944766	-0.798761
71	1	0	-7.007028	-1.970046	-0.906277
72	6	0	-8.196065	-0.252856	-1.516027
73	1	0	-7.905937	0.791905	-1.654812
74	1	0	-8.324312	-0.687336	-2.511039
75	6	0	-9.569527	-0.228074	-0.868571
76	8	0	-10.457933	0.449992	-1.670217
77	1	0	-11.296651	0.436191	-1.190198
78	8	0	-9.918243	-0.706219	0.173144
79	6	0	-5.669024	-2.379912	1.419092
80	6	0	-6.293365	-3.596977	0.666456
81	8	0	-6.573450	-4.573739	1.374337
82	8	0	-6.459450	-3.493624	-0.593516
83	1	0	-6.395096	-2.061535	2.170230
84	1	0	-4.810479	-2.758909	1.974729
85	1	0	-4.511149	-2.401934	-0.888244
86	1	0	0.837510	-0.296508	1.361863

36. **Table S36:** Cartesian Coordinates for the low-energy geometry of the **PEDK+GH** hexapeptide calculated using the B3LYP method and the 6-311+G(*2df,2pd*) level of theory. Final Energy = -1490147 kcal/mol, Relative Energy = 84714 kcal/mol, Net Charge = -1, Multiplicity = 1.

Standard Orientation					
Center Number	Atomic Number	Atomic Type	Coordinates (Angstroms)		
			X	Y	Z
1	6	0	4.910310	-5.205473	-0.390965
2	7	0	4.253992	-4.017952	-0.936771
3	1	0	3.929185	-4.138740	-1.886978
4	6	0	5.268730	-2.956404	-0.859409
5	1	0	6.070918	-3.137273	-1.596177
6	6	0	5.853232	-3.152755	0.550402
7	6	0	5.638459	-4.660069	0.851928
8	1	0	6.574986	-5.187940	1.027891
9	1	0	5.023412	-4.788913	1.742375
10	1	0	6.897797	-2.857922	0.599558
11	1	0	5.315269	-2.530544	1.262698
12	6	0	4.688534	-1.588550	-1.209984
13	8	0	3.713897	-1.571172	-2.042360
14	7	0	5.259229	-0.540934	-0.688836
15	6	0	4.775081	0.754864	-1.117500
16	6	0	5.786115	1.851600	-0.706715
17	6	0	6.123236	1.930912	0.797422
18	6	0	7.303849	1.082189	1.269756
19	8	0	7.334392	-0.175301	0.854491
20	1	0	6.520279	-0.398966	0.261664
21	8	0	8.157238	1.538961	1.999421
22	1	0	5.251014	1.638648	1.388171
23	1	0	6.365287	2.955233	1.071470
24	1	0	5.392939	2.819241	-1.024307
25	1	0	6.699483	1.688827	-1.282379
26	1	0	4.680500	0.778526	-2.210270
27	6	0	3.384743	1.108662	-0.550632
28	8	0	2.962975	0.694350	0.521446
29	7	0	2.670554	1.979987	-1.313236
30	6	0	1.482385	2.700975	-0.857270
31	6	0	0.367765	1.721297	-0.417189
32	7	0	-0.284338	2.037138	0.662787
33	6	0	-1.303046	1.176860	1.256684
34	6	0	-0.760740	-0.217063	1.721836
35	1	0	-1.161568	-0.417888	2.717900
36	1	0	0.310769	-0.069132	1.852260
37	6	0	-1.034647	-1.442473	0.832773
38	1	0	-2.004107	-1.875112	1.091492
39	6	0	0.033407	-2.540310	0.936509
40	1	0	0.181025	-2.831302	1.980484
41	6	0	1.402468	-2.169716	0.370095
42	1	0	1.869757	-1.350544	0.911468
43	7	0	1.339594	-1.723968	-1.053548
44	1	0	0.806651	-2.378094	-1.619683
45	1	0	2.344304	-1.667800	-1.474608
46	1	0	0.905149	-0.777432	-1.139812
47	1	0	2.082605	-3.019781	0.403166

48	1	0	-0.331845	-3.436981	0.424053
49	1	0	-1.122913	-1.140216	-0.207162
50	6	0	-2.618790	1.072269	0.480456
51	8	0	-3.711735	1.090259	1.071551
52	7	0	-2.546087	0.977415	-0.855252
53	6	0	-3.730914	0.990266	-1.673185
54	1	0	-3.428800	0.857832	-2.712120
55	1	0	-4.243543	1.952761	-1.594165
56	6	0	-4.698858	-0.141670	-1.331775
57	8	0	-4.367892	-1.303203	-1.204562
58	7	0	-6.014963	0.236846	-1.204123
59	1	0	-6.282595	1.207356	-1.239035
60	6	0	-7.000236	-0.723700	-0.809720
61	1	0	-6.790525	-1.664103	-1.331149
62	6	0	-6.935417	-1.006973	0.675889
63	6	0	-6.045130	-0.543727	1.595466
64	7	0	-6.365192	-1.162023	2.785262
65	6	0	-7.405705	-1.975305	2.622525
66	7	0	-7.763895	-1.894556	1.342647
67	1	0	-8.551790	-2.366343	0.921977
68	1	0	-7.875844	-2.575973	3.379350
69	1	0	-5.869156	-1.023665	3.653186
70	1	0	-5.194028	0.127446	1.480468
71	6	0	-8.392981	-0.260152	-1.234319
72	8	0	-8.650587	0.787926	-1.743149
73	8	0	-9.317923	-1.211519	-0.948283
74	1	0	-10.183646	-0.889458	-1.243766
75	1	0	-1.605038	0.867024	-1.261442
76	1	0	-1.610777	1.694020	2.165346
77	8	0	0.132406	0.738460	-1.204627
78	1	0	1.089024	3.193167	-1.750686
79	6	0	1.884369	3.788025	0.168658
80	6	0	0.904703	4.948337	0.344964
81	8	0	1.197647	6.082818	0.043411
82	8	0	-0.276069	4.636557	0.867475
83	1	0	-0.328995	3.617637	0.932426
84	1	0	2.817057	4.234692	-0.165343
85	1	0	2.057218	3.308991	1.132590
86	1	0	3.054180	2.241115	-2.204680
87	1	0	5.642416	-5.635933	-1.091778
88	1	0	4.181360	-5.982726	-0.155691

37. **Table S37:** Cartesian Coordinates for the low-energy geometry of the **PE+DKGH** hexapeptide calculated using the B3LYP method and the 6-311+G(*2df,2pd*) level of theory. Final Energy = -1490210 kcal/mol, Relative Energy = 84651 kcal/mol, Net Charge = -1, Multiplicity = 1.

Standard Orientation					
Center Number	Atomic Number	Atomic Type	Coordinates (Angstroms)		
			X	Y	Z
1	6	0	-0.228737	-3.244215	-1.304250
2	7	0	-0.546330	-1.856825	-0.938784
3	6	0	-1.615423	-1.217251	-1.389880
4	6	0	-1.850064	0.240550	-0.919998
5	7	0	-3.208925	0.223911	-0.366754
6	1	0	-3.848999	-0.208726	-1.018878
7	6	0	-3.578390	0.016980	0.928461
8	6	0	-2.997434	0.874530	2.063778
9	7	0	-2.334217	2.079113	1.611390
10	6	0	-3.067873	3.121372	1.151808
11	6	0	-2.341402	4.403706	0.760280
12	7	0	-2.638475	4.693403	-0.646606
13	6	0	-1.786315	5.829888	-0.994217
14	6	0	-0.442378	5.479614	-0.327246
15	6	0	-0.806723	4.482502	0.806689
16	1	0	-0.454299	4.809808	1.783293
17	1	0	-0.349273	3.516371	0.606083
18	1	0	0.056578	6.371013	0.050455
19	1	0	0.223811	5.002496	-1.042480
20	1	0	-2.173248	6.776868	-0.588482
21	1	0	-1.708125	5.935886	-2.076039
22	1	0	-3.625848	4.862606	-0.787905
23	1	0	-2.757041	5.178624	1.426029
24	8	0	-4.286530	3.057424	1.033222
25	1	0	-1.332014	2.139769	1.780672
26	6	0	-2.153344	0.090676	3.090779
27	6	0	-0.951173	-0.703725	2.578792
28	6	0	0.191080	0.079321	1.902620
29	8	0	0.307356	1.292000	2.114652
30	8	0	0.942386	-0.619049	1.139481
31	1	0	-1.273803	-1.493358	1.899440
32	1	0	-0.497304	-1.225359	3.426443
33	1	0	-2.830397	-0.601058	3.593629
34	1	0	-1.825063	0.811873	3.839329
35	1	0	-3.913961	1.179957	2.574881
36	8	0	-4.482514	-0.790068	1.190572
37	1	0	-1.149642	0.508987	-0.137499
38	6	0	-1.736579	1.252635	-2.076296
39	6	0	-0.306263	1.294780	-2.583580
40	8	0	0.260175	2.487254	-2.468168
41	1	0	1.255001	2.421301	-2.594088
42	8	0	0.254937	0.305554	-3.004843
43	1	0	-2.058845	2.232141	-1.728250
44	1	0	-2.383411	0.931673	-2.894672
45	8	0	-2.504074	-1.757416	-2.082186
46	1	0	0.087141	-1.331632	-0.328085
47	6	0	-1.353425	-4.256317	-0.947917

48	1	0	-0.918382	-5.251808	-1.035757
49	1	0	-2.109230	-4.177886	-1.725947
50	6	0	-1.978711	-4.051136	0.434509
51	1	0	-1.304558	-4.419816	1.209322
52	6	0	-3.350386	-4.703555	0.653878
53	1	0	-3.265087	-5.792532	0.631388
54	6	0	-4.441332	-4.346134	-0.355624
55	1	0	-4.271096	-4.831429	-1.314532
56	7	0	-4.542401	-2.873788	-0.649806
57	1	0	-4.583436	-2.233663	0.184641
58	1	0	-5.383615	-2.688473	-1.192048
59	1	0	-3.732715	-2.534446	-1.231924
60	1	0	-5.412441	-4.669406	0.014693
61	1	0	-3.698360	-4.445951	1.656522
62	1	0	-2.079620	-2.983988	0.631859
63	6	0	1.128401	-3.755814	-0.775317
64	8	0	1.599611	-4.737491	-1.328953
65	7	0	1.705834	-3.124019	0.270363
66	6	0	3.043268	-3.465206	0.720956
67	1	0	3.356366	-4.377411	0.223035
68	1	0	3.038270	-3.653326	1.797499
69	6	0	4.132373	-2.417486	0.459997
70	8	0	5.297163	-2.765992	0.364443
71	7	0	3.733006	-1.119999	0.366123
72	1	0	2.791150	-0.839128	0.640155
73	6	0	4.708774	-0.075246	0.206901
74	1	0	5.596219	-0.532080	-0.243373
75	6	0	4.162228	1.003702	-0.690189
76	6	0	3.098946	0.972746	-1.546970
77	7	0	2.946170	2.195937	-2.159080
78	6	0	3.909161	2.957113	-1.691832
79	7	0	4.676709	2.277759	-0.803659
80	1	0	5.463836	2.629787	-0.284645
81	1	0	4.087657	3.986843	-1.949898
82	1	0	2.429973	0.160278	-1.759358
83	6	0	5.190393	0.445210	1.571547
84	8	0	4.819477	0.067922	2.642006
85	8	0	6.159908	1.393016	1.421360
86	1	0	6.447541	1.647405	2.310575
87	1	0	1.259452	-2.324316	0.719709
88	1	0	-0.107990	-3.277037	-2.386901

38.

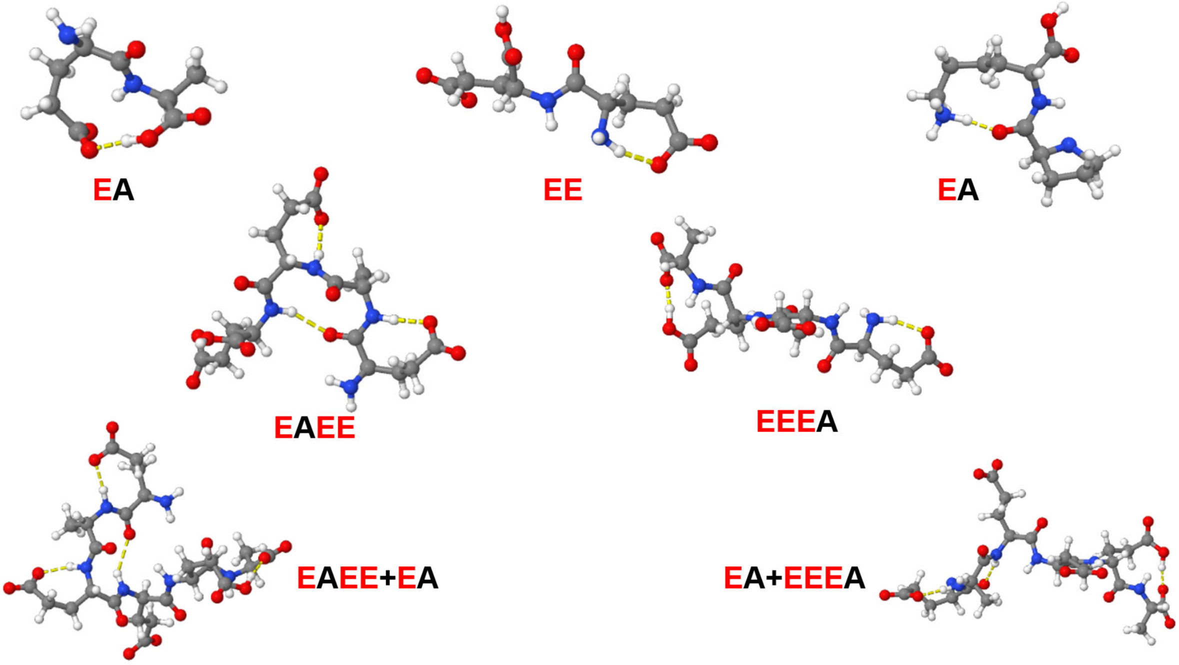

Figure S1: All structures optimized at the 6-311+G(2df,pd) basis set used in the buildup of the βI E-hook hexamers.

39.

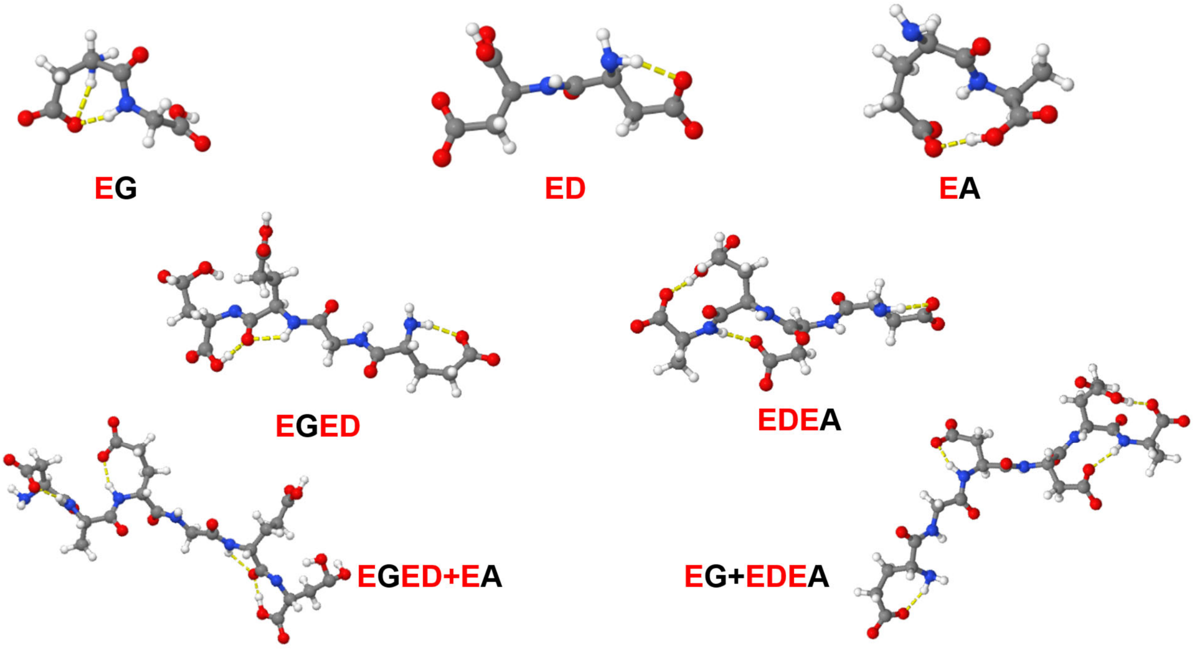

Figure S2: All structures optimized at the 6-311+G(2df,pd) basis set used in the buildup of the βII E-hook hexamers.

40.

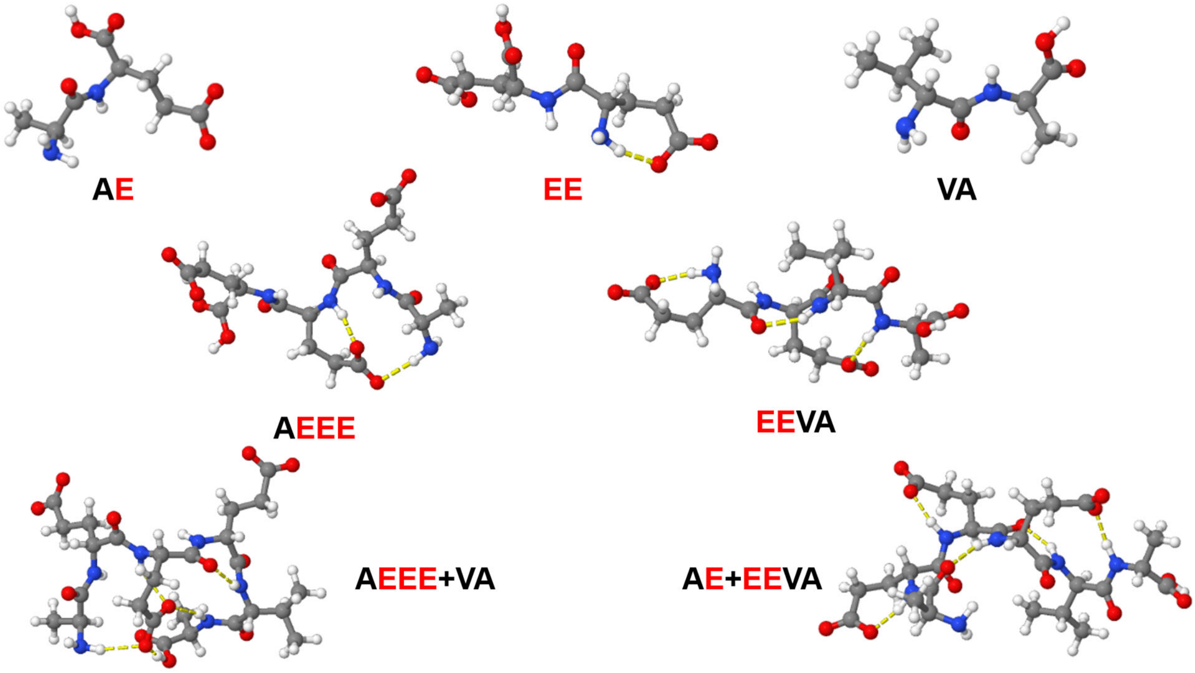

Figure S3: All structures optimized at the 6-311+G(2df,pd) basis set used in the buildup of the βIV E-hook hexamers.

41.

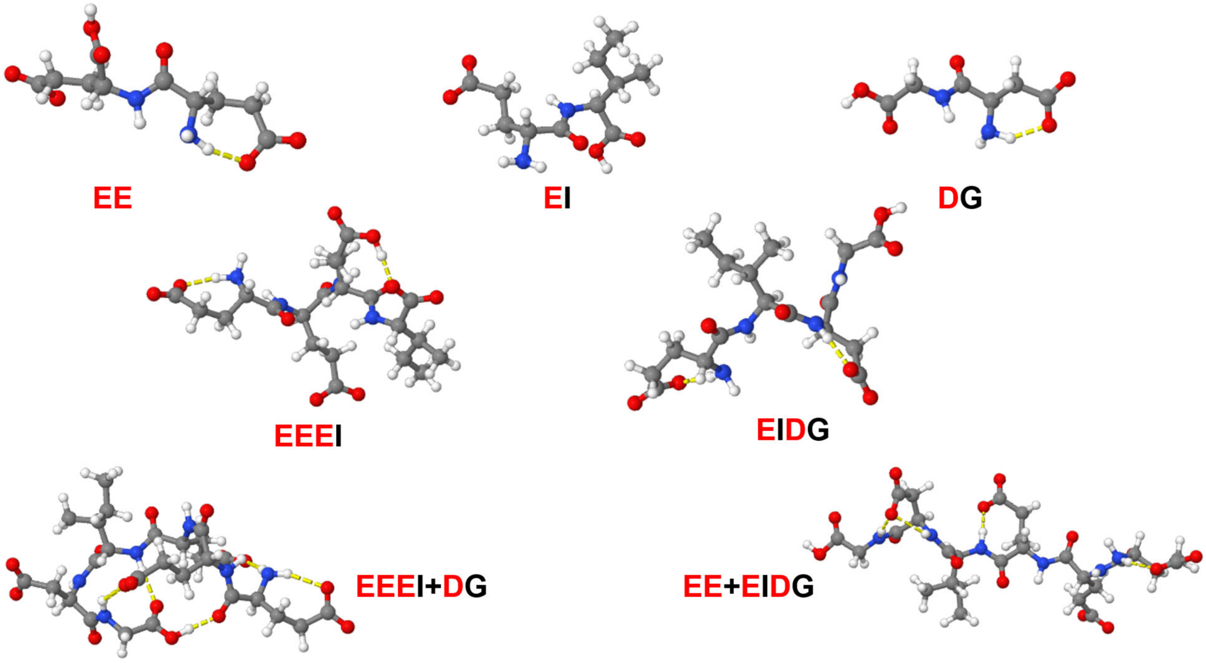

Figure S4: All structures optimized at the 6-311+G(2df,pd) basis set used in the buildup of the βV E-hook hexamers.

42.

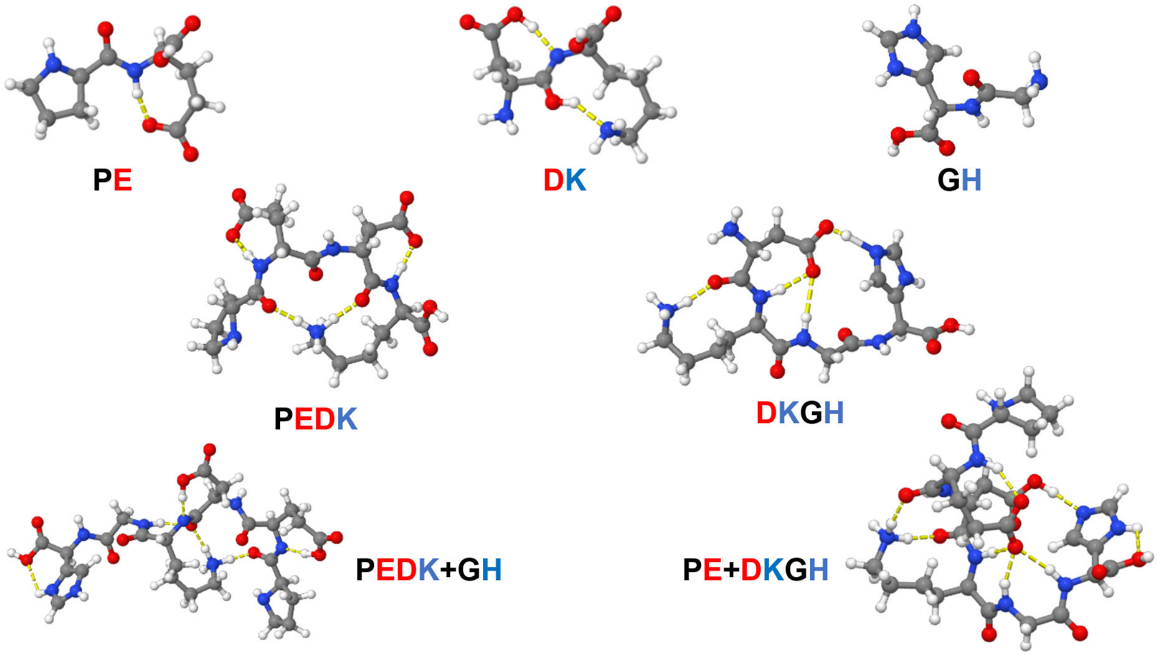

Figure S5: All structures optimized at the 6-311+G(2df,pd) basis set used in the buildup of the βVI E-hook hexamers.

43. Python code used in the dynamic time warping analysis of the experimental and computed Raman spectra:

```
import os
import numpy as np
import matplotlib.pyplot as plt

from scipy.ndimage import gaussian_filter1d
from scipy.signal import find_peaks
from scipy.stats import pearsonr

def calculate_peak_metrics(results):
    matched_exp = results["matched_exp_peak_indices"]
    exp_peaks = results["exp_peaks"]
    assignments = results["dtw_peak_assignments"]

    match_fraction = len(matched_exp) / len(exp_peaks) if len(exp_peaks) > 0 else np.nan

    shifts = np.array([item["shift"] for item in assignments])

    peak_rms = np.sqrt(np.mean(shifts**2)) if len(shifts) > 0 else np.nan

    return match_fraction, peak_rms

################################################################################
# GRAPH / ANALYSIS OPTIONS
################################################################################

GRAPH = {
    "figsize": (32, 16),
    "dpi": 300,
    "save_dpi": 1200,
    "spectrum_linewidth": 2.5,
    "match_linewidth": 1.5,
    "warped_match_linewidth": 1.0,
    "marker_size": 100,
    "axis_fontsize": 32,
    "tick_fontsize": 26,
    "legend_fontsize": 24,
    "matched_color": "black",
    "unmatched_color": "red",
    "match_line_color": "black",
    "match_line_alpha": 0.7,
    "overall_original_title": "PEDKGH Experiment vs PE+DKGH Simulation",
    "overall_warped_title": "DTW-Warped Spectra: PEDKGH vs PE+DKGH",
}

FINGERPRINT_REGION = (200, 2000)
```

```
EXP_SMOOTH_SIGMA = 4
SIM_SMOOTH_SIGMA = 2

DTW_WINDOW = 50
EXP_PEAK_SNAP_TOLERANCE = 40

EXP_PEAK_HEIGHT = 0.002
EXP_PEAK_PROMINENCE = 0.002
EXP_PEAK_DISTANCE = 10

SIM_PEAK_HEIGHT = 0.002
SIM_PEAK_PROMINENCE = 0.002
SIM_PEAK_DISTANCE = 5

WAVENUMBER_WEIGHT = 2.0
INTENSITY_WEIGHT = 100.0
COVERAGE_BONUS = 10.0
OCCUPANCY_PENALTY_WEIGHT = 15.0

SAVE_ORIGINAL_FIGURE = True
SAVE_WARPED_FIGURE = True
ORIGINAL_FIGURE_NAME = "Originals.svg"
WARPED_FIGURE_NAME = "Warped.svg"

PRINT_FIRST_PASS_ATTEMPTS = True
PRINT_SECOND_PASS_ATTEMPTS = True

NEWFILENAME = input("Select new figure name: ")

ORIGINAL_FIGURE_NAME = NEWFILENAME + ORIGINAL_FIGURE_NAME
WARPED_FIGURE_NAME = NEWFILENAME + WARPED_FIGURE_NAME

##################################################################################
# LIST TXT FILES AND SELECT INPUTS
##################################################################################

script_dir = os.path.dirname(os.path.abspath(__file__))

txt_files = [
    file for file in os.listdir(script_dir)
    if file.lower().endswith(".txt")
]

if len(txt_files) < 2:
    raise RuntimeError("Need at least two .txt files in this directory.")

print("\nAvailable .txt files:\n")
```

```
for i, file in enumerate(txt_files):
    print(f"{i}: {file}")

exp_choice = int(input("\nEnter number for experimental spectrum: "))
sim_choice = int(input("Enter number for simulated spectrum: "))

exp_file = os.path.join(script_dir, txt_files[exp_choice])
sim_file = os.path.join(script_dir, txt_files[sim_choice])

print(f"\nExperimental file: {txt_files[exp_choice]}")
print(f"Simulated file:    {txt_files[sim_choice]}")

###############################################################################
# LOAD DATA
###############################################################################

exp_data = np.loadtxt(exp_file)
sim_data = np.loadtxt(sim_file)

exp_x_raw = exp_data[:, 0]
exp_y_raw = exp_data[:, 1]

sim_x_raw = sim_data[:, 0]
sim_y_raw = sim_data[:, 1]

###############################################################################
# REGION PREPROCESSING FUNCTION
###############################################################################

def prepare_region(exp_x_raw, exp_y_raw, sim_x_raw, sim_y_raw, xmin, xmax):
    """
    Restrict, interpolate, vector normalize, and smooth one spectral region.

    The experimental and simulated spectra are normalized separately within the
    requested region.
    """

    exp_mask = (exp_x_raw >= xmin) & (exp_x_raw <= xmax)
    sim_mask = (sim_x_raw >= xmin) & (sim_x_raw <= xmax)

    exp_x_region = exp_x_raw[exp_mask]
    exp_y_region = exp_y_raw[exp_mask]

    sim_x_region = sim_x_raw[sim_mask]
    sim_y_region = sim_y_raw[sim_mask]

    if len(exp_x_region) == 0:
```

```
        raise ValueError(f"No experimental data found in {xmin}-{xmax} cm^-1.")

    if len(sim_x_region) == 0:
        raise ValueError(f"No simulated data found in {xmin}-{xmax} cm^-1.")

    sim_y_interp_region = np.interp(
        exp_x_region,
        sim_x_region,
        sim_y_region
    )

    # Remove offsets before vector normalization.
    exp_y_region = exp_y_region - np.min(exp_y_region)
    sim_y_interp_region = sim_y_interp_region - np.min(sim_y_interp_region)

    exp_norm = np.linalg.norm(exp_y_region)
    sim_norm = np.linalg.norm(sim_y_interp_region)

    if exp_norm == 0:
        raise ValueError(f"Experimental spectrum norm is zero in {xmin}-{xmax} cm^-1.")

    if sim_norm == 0:
        raise ValueError(f"Simulated spectrum norm is zero in {xmin}-{xmax} cm^-1.")

    exp_y_region = exp_y_region / exp_norm
    sim_y_interp_region = sim_y_interp_region / sim_norm

    exp_y_region = gaussian_filter1d(exp_y_region, sigma=EXP_SMOOTH_SIGMA)
    sim_y_interp_region = gaussian_filter1d(sim_y_interp_region, sigma=SIM_SMOOTH_SIGMA)

    return exp_x_region, exp_y_region, sim_y_interp_region

###############################################################################
# DTW FUNCTIONS
###############################################################################

def dtw_distance(y1, y2, window=None):
    n = len(y1)
    m = len(y2)

    if window is None:
        window = max(n, m)
    else:
        window = max(window, abs(n - m))

    dtw = np.full((n + 1, m + 1), np.inf)
    dtw[0, 0] = 0
```

```
    for i in range(1, n + 1):

        j_start = max(1, i - window)
        j_end = min(m, i + window)

        for j in range(j_start, j_end + 1):

            cost = abs(y1[i - 1] - y2[j - 1])

            dtw[i, j] = cost + min(
                dtw[i - 1, j],
                dtw[i, j - 1],
                dtw[i - 1, j - 1]
            )

    return dtw[n, m], dtw

def get_dtw_path(dtw_matrix):
    i = dtw_matrix.shape[0] - 1
    j = dtw_matrix.shape[1] - 1

    path = []

    while i > 0 and j > 0:

        path.append((i - 1, j - 1))

        steps = [
            dtw_matrix[i - 1, j],
            dtw_matrix[i, j - 1],
            dtw_matrix[i - 1, j - 1]
        ]

        best_step = np.argmin(steps)

        if best_step == 0:
            i -= 1
        elif best_step == 1:
            j -= 1
        else:
            i -= 1
            j -= 1

    path.reverse()

    return np.array(path)

##########################################################################################
```

```
# REGION ANALYSIS FUNCTION
###################################################################################

def analyze_region(region_name, exp_x_region, exp_y_region, sim_y_interp_region):
    """
    Run DTW, peak finding, and DTW-derived peak assignment for one region.
    """

    dtw_score, dtw_matrix = dtw_distance(
        exp_y_region,
        sim_y_interp_region,
        window=DTW_WINDOW
    )

    dtw_score_norm = dtw_score / len(exp_y_region)
    dtw_path = get_dtw_path(dtw_matrix)

    exp_path_indices = dtw_path[:, 0]
    sim_path_indices = dtw_path[:, 1]

    warped_x_exp = exp_x_region[exp_path_indices]
    warped_exp_y = exp_y_region[exp_path_indices]
    warped_sim_y = sim_y_interp_region[sim_path_indices]
    warped_sim_original_x = exp_x_region[sim_path_indices]

    pearson_original, _ = pearsonr(exp_y_region, sim_y_interp_region)
    pearson_warped, _ = pearsonr(warped_exp_y, warped_sim_y)

    exp_peaks, _ = find_peaks(
        exp_y_region,
        height=EXP_PEAK_HEIGHT,
        prominence=EXP_PEAK_PROMINENCE,
        distance=EXP_PEAK_DISTANCE
    )

    sim_peaks, _ = find_peaks(
        sim_y_interp_region,
        height=SIM_PEAK_HEIGHT,
        prominence=SIM_PEAK_PROMINENCE,
        distance=SIM_PEAK_DISTANCE
    )

    exp_peak_positions = exp_x_region[exp_peaks]
    exp_peak_intensities = exp_y_region[exp_peaks]

    sim_peak_positions = exp_x_region[sim_peaks]
    sim_peak_intensities = sim_y_interp_region[sim_peaks]
```

```
if len(exp_peaks) == 0:
    raise RuntimeError(
        f"No experimental peaks detected in {region_name}. "
        "Lower experimental height/prominence thresholds."
    )

if len(sim_peaks) == 0:
    raise RuntimeError(
        f"No simulated peaks detected in {region_name}. "
        "Lower simulated height/prominence thresholds."
    )

################################################################################
# PASS 1: BUILD CANDIDATES AND INITIAL DISTANCE-ONLY ASSIGNMENTS
################################################################################

candidate_records = []
initial_matched_exp_peak_indices = set()

for sim_peak_index in sim_peaks:

    matching_rows = dtw_path[dtw_path[:, 1] == sim_peak_index]

    if len(matching_rows) == 0:
        continue

    aligned_exp_indices = matching_rows[:, 0]
    aligned_exp_position = np.mean(exp_x_region[aligned_exp_indices])

    exp_peak_distances = np.abs(exp_peak_positions - aligned_exp_position)

    candidate_mask = exp_peak_distances <= EXP_PEAK_SNAP_TOLERANCE
    candidate_indices = np.where(candidate_mask)[0]

    if len(candidate_indices) == 0:
        continue

    candidate_distances = exp_peak_distances[candidate_indices]

    if PRINT_FIRST_PASS_ATTEMPTS:
        print("\n====================================================================")
        print(
            f"{region_name.upper()} FIRST PASS MATCH ATTEMPTS "
            f"FOR SIMULATED PEAK {exp_x_region[sim_peak_index]:.2f} cm^-1"
        )
        print("====================================================================")
        print("Exp Peak      DTW/Wavenumber Distance")
        print("-" * 45)
```

```
        for i, candidate_index in enumerate(candidate_indices):
            candidate_position = exp_peak_positions[candidate_index]
            candidate_distance = candidate_distances[i]

            print(
                f"{candidate_position:8.2f}      "
                f"{candidate_distance:12.6f}"
            )

    initial_best_local_index = np.argmin(candidate_distances)
    initial_best_exp_index = candidate_indices[initial_best_local_index]

    initial_best_position = exp_peak_positions[initial_best_exp_index]
    initial_best_distance = candidate_distances[initial_best_local_index]

    if PRINT_FIRST_PASS_ATTEMPTS:
        print("\nFIRST PASS CHOICE")
        print(f"Simulated Peak:    {exp_x_region[sim_peak_index]:.2f} cm^-1")
        print(f"Experimental Peak: {initial_best_position:.2f} cm^-1")
        print(f"Distance:          {initial_best_distance:.6f}")

    initial_matched_exp_peak_indices.add(
        exp_peaks[initial_best_exp_index]
    )

    candidate_records.append(
        {
            "sim_peak_index": sim_peak_index,
            "aligned_exp_indices": aligned_exp_indices,
            "candidate_indices": candidate_indices,
            "candidate_distances": candidate_distances,
            "num_aligned_points": len(aligned_exp_indices)
        }
    )

################################################################################
# DETERMINE WHICH EXPERIMENTAL PEAKS WERE LEFT UNCOVERED IN PASS 1
################################################################################

initial_unmatched_exp_peak_indices = set(exp_peaks) - initial_matched_exp_peak_indices

################################################################################
# PASS 2: SOFT MANY-TO-ONE ASSIGNMENT
################################################################################

dtw_peak_assignments = []
```

```
exp_assignment_counts = {}

combined_min_intensity = min(np.min(exp_y_region), np.min(sim_y_interp_region))
combined_max_intensity = max(np.max(exp_y_region), np.max(sim_y_interp_region))

intensity_span = combined_max_intensity - combined_min_intensity

if intensity_span == 0:
    raise ValueError(f"Intensity span is zero in {region_name}; cannot scale intensity differences.")

for record in candidate_records:

    sim_peak_index = record["sim_peak_index"]
    candidate_indices = record["candidate_indices"]
    candidate_distances = record["candidate_distances"]

    sim_intensity = sim_y_interp_region[sim_peak_index]

    candidate_exp_intensities = exp_peak_intensities[candidate_indices]

    wavenumber_term = WAVENUMBER_WEIGHT * candidate_distances

    raw_intensity_differences = np.abs(
        candidate_exp_intensities - sim_intensity
    )

    scaled_intensity_differences = (
        raw_intensity_differences / intensity_span
    )

    intensity_term = INTENSITY_WEIGHT * scaled_intensity_differences

    coverage_term = np.zeros_like(candidate_distances)

    for i, candidate_index in enumerate(candidate_indices):

        true_exp_peak_index = exp_peaks[candidate_index]

        if true_exp_peak_index in initial_unmatched_exp_peak_indices:
            coverage_term[i] = -COVERAGE_BONUS

    occupancy_term = np.zeros_like(candidate_distances)

    for i, candidate_index in enumerate(candidate_indices):

        true_exp_peak_index = exp_peaks[candidate_index]

        current_assignment_count = exp_assignment_counts.get(
```

```
            true_exp_peak_index,
            0
        )

        occupancy_term[i] = (
            OCCUPANCY_PENALTY_WEIGHT * current_assignment_count
        )

    candidate_scores = (
        wavenumber_term
        + intensity_term
        + coverage_term
        + occupancy_term
    )

    if PRINT_SECOND_PASS_ATTEMPTS:
        print("\n===============================================================")
        print(f"{region_name.upper()} SIMULATED PEAK: {exp_x_region[sim_peak_index]:.2f} cm^-1")
        print("===============================================================")

        print(
            "Exp Peak     "
            "Wave Term    "
            "Intensity Δ  "
            "Scaled Int Δ "
            "Intensity Term   "
            "Coverage Term   "
            "Occupancy Term   "
            "Final Score"
        )

        print("-" * 130)

        for i, candidate_index in enumerate(candidate_indices):

            exp_peak_position = exp_peak_positions[candidate_index]

            print(
                f"{exp_peak_position:8.2f}     "
                f"{wavenumber_term[i]:10.3f}   "
                f"{raw_intensity_differences[i]:10.6f}   "
                f"{scaled_intensity_differences[i]:10.6f}   "
                f"{intensity_term[i]:14.6f}   "
                f"{coverage_term[i]:14.3f}   "
                f"{occupancy_term[i]:14.3f}   "
                f"{candidate_scores[i]:11.6f}"
            )
```

```
    best_candidate_local_index = np.argmin(candidate_scores)
    nearest_exp_peak_index = candidate_indices[best_candidate_local_index]

    matched_exp_position = exp_peak_positions[nearest_exp_peak_index]
    matched_exp_intensity = exp_peak_intensities[nearest_exp_peak_index]

    sim_position = exp_x_region[sim_peak_index]

    shift = sim_position - matched_exp_position

    matched_true_exp_peak_index = exp_peaks[nearest_exp_peak_index]

    exp_assignment_counts[matched_true_exp_peak_index] = (
        exp_assignment_counts.get(matched_true_exp_peak_index, 0) + 1
    )

    dtw_peak_assignments.append(
        {
            "sim_peak": sim_position,
            "sim_peak_index": sim_peak_index,
            "sim_intensity": sim_intensity,
            "matched_exp_peak": matched_exp_position,
            "matched_exp_peak_index": matched_true_exp_peak_index,
            "matched_exp_intensity": matched_exp_intensity,
            "shift": shift,
            "num_aligned_points": record["num_aligned_points"],
            "assignment_score": candidate_scores[best_candidate_local_index],
            "wavenumber_term": wavenumber_term[best_candidate_local_index],
            "raw_intensity_difference": raw_intensity_differences[best_candidate_local_index],
            "scaled_intensity_difference": scaled_intensity_differences[best_candidate_local_index],
            "intensity_term": intensity_term[best_candidate_local_index],
            "coverage_term": coverage_term[best_candidate_local_index],
            "occupancy_term": occupancy_term[best_candidate_local_index]
        }
    )

##############################################################################
# MATCHED / UNMATCHED PEAKS
##############################################################################

matched_exp_peak_indices = set()
matched_sim_peak_indices = set()

for item in dtw_peak_assignments:

    matched_exp_peak_indices.add(
        item["matched_exp_peak_index"]
    )
```

```
    matched_sim_peak_indices.add(
        item["sim_peak_index"]
    )

unmatched_exp_peak_indices = [
    peak_index
    for peak_index in exp_peaks
    if peak_index not in matched_exp_peak_indices
]

unmatched_sim_peak_indices = [
    peak_index
    for peak_index in sim_peaks
    if peak_index not in matched_sim_peak_indices
]

print(f"\n================ {region_name.upper()} DTW RESULTS ================\n")
print(f"Region analyzed:         {exp_x_region[0]:.2f}–{exp_x_region[-1]:.2f} cm^-1")
print(f"DTW window:              {DTW_WINDOW} data points")
print(f"Raw DTW score:           {dtw_score:.4f}")
print(f"Normalized DTW score:    {dtw_score_norm:.6f}")
print(f"Original Pearson r:      {pearson_original:.4f}")
print(f"DTW-warped Pearson r:    {pearson_warped:.4f}")

print(f"\n========== {region_name.upper()} DTW PEAK SHIFT ASSIGNMENTS ==========\n")
print("Sim Peak   Matched Exp Peak   Shift   Sim Int   Exp Int   Points")
print("-----------------------------------------------------------------")

for item in dtw_peak_assignments:
    print(
        f"{item['sim_peak']:8.2f}   "
        f"{item['matched_exp_peak']:16.2f}   "
        f"{item['shift']:7.2f}   "
        f"{item['sim_intensity']:7.3f}   "
        f"{item['matched_exp_intensity']:7.3f}   "
        f"{item['num_aligned_points']:6d}"
    )

print(f"\n================ {region_name.upper()} PEAK COUNTS ================\n")
print(f"Total experimental peaks found: {len(exp_peaks)}")
print(f"Total simulated peaks found:    {len(sim_peaks)}")

return {
    "region_name": region_name,
    "x": exp_x_region,
    "exp_y": exp_y_region,
    "sim_y": sim_y_interp_region,
```

```
        "dtw_score": dtw_score,
        "dtw_score_norm": dtw_score_norm,
        "dtw_path": dtw_path,
        "exp_path_indices": exp_path_indices,
        "sim_path_indices": sim_path_indices,
        "warped_x_exp": warped_x_exp,
        "warped_exp_y": warped_exp_y,
        "warped_sim_y": warped_sim_y,
        "warped_sim_original_x": warped_sim_original_x,
        "pearson_original": pearson_original,
        "pearson_warped": pearson_warped,
        "exp_peaks": exp_peaks,
        "sim_peaks": sim_peaks,
        "exp_peak_positions": exp_peak_positions,
        "exp_peak_intensities": exp_peak_intensities,
        "sim_peak_positions": sim_peak_positions,
        "sim_peak_intensities": sim_peak_intensities,
        "dtw_peak_assignments": dtw_peak_assignments,
        "matched_exp_peak_indices": matched_exp_peak_indices,
        "matched_sim_peak_indices": matched_sim_peak_indices,
        "unmatched_exp_peak_indices": unmatched_exp_peak_indices,
        "unmatched_sim_peak_indices": unmatched_sim_peak_indices,
    }

###############################################################################
# PREPARE REGION
###############################################################################

fingerprint_xmin, fingerprint_xmax = FINGERPRINT_REGION

fp_exp_x, fp_exp_y, fp_sim_y = prepare_region(
    exp_x_raw,
    exp_y_raw,
    sim_x_raw,
    sim_y_raw,
    fingerprint_xmin,
    fingerprint_xmax
)

###############################################################################
# ANALYZE REGION
###############################################################################

fingerprint_results = analyze_region(
    "fingerprint",
    fp_exp_x,
    fp_exp_y,
    fp_sim_y
```

```
)

################################################################################
# SCORING METRICS
################################################################################

fp_match_fraction, fp_peak_rms = calculate_peak_metrics(fingerprint_results)

print("\n================ SCORING SUMMARY ================\n")

print("Fingerprint Region")
print(f"Peak match fraction:     {fp_match_fraction:.4f}")
print(f"Pearson original:        {fingerprint_results['pearson_original']:.4f}")
print(f"Pearson DTW-warped:      {fingerprint_results['pearson_warped']:.4f}")
print(f"Peak shift RMSD:         {fp_peak_rms:.4f} cm^-1")

################################################################################
# PLOTTING HELPERS
################################################################################

def get_marker_arrays(results):
    x = results["x"]
    exp_y = results["exp_y"]
    sim_y = results["sim_y"]

    matched_exp_indices = list(results["matched_exp_peak_indices"])
    matched_sim_indices = list(results["matched_sim_peak_indices"])

    unmatched_exp_indices = results["unmatched_exp_peak_indices"]
    unmatched_sim_indices = results["unmatched_sim_peak_indices"]

    return {
        "matched_exp_positions": x[matched_exp_indices],
        "matched_exp_intensities": exp_y[matched_exp_indices],
        "unmatched_exp_positions": x[unmatched_exp_indices],
        "unmatched_exp_intensities": exp_y[unmatched_exp_indices],
        "matched_sim_positions": x[matched_sim_indices],
        "matched_sim_intensities": sim_y[matched_sim_indices],
        "unmatched_sim_positions": x[unmatched_sim_indices],
        "unmatched_sim_intensities": sim_y[unmatched_sim_indices],
    }

def plot_region_on_axis(ax, results, include_legend=False):
    x = results["x"]
    exp_y = results["exp_y"]
    sim_y = results["sim_y"]
    markers = get_marker_arrays(results)
```

```
ax.plot(
    x,
    exp_y,
    linewidth=GRAPH["spectrum_linewidth"],
    label="Experimental"
)

ax.plot(
    x,
    sim_y,
    linewidth=GRAPH["spectrum_linewidth"],
    label="Simulated"
)

ax.scatter(
    markers["matched_exp_positions"],
    markers["matched_exp_intensities"],
    marker="o",
    s=GRAPH["marker_size"],
    color=GRAPH["matched_color"],
    edgecolors=GRAPH["matched_color"],
    linewidths=1.5,
    zorder=5,
    label="Matched Experimental Peaks"
)

ax.scatter(
    markers["unmatched_exp_positions"],
    markers["unmatched_exp_intensities"],
    marker="o",
    s=GRAPH["marker_size"],
    color=GRAPH["unmatched_color"],
    edgecolors=GRAPH["unmatched_color"],
    linewidths=1.5,
    zorder=5,
    label="Unmatched Experimental Peaks"
)

ax.scatter(
    markers["matched_sim_positions"],
    markers["matched_sim_intensities"],
    marker="x",
    s=GRAPH["marker_size"],
    color=GRAPH["matched_color"],
    linewidths=2.0,
    zorder=5,
    label="Matched Simulated Peaks"
)
```

```
    ax.scatter(
        markers["unmatched_sim_positions"],
        markers["unmatched_sim_intensities"],
        marker="x",
        s=GRAPH["marker_size"],
        color=GRAPH["unmatched_color"],
        linewidths=2.0,
        zorder=5,
        label="Unmatched Simulated Peaks"
    )

    for item in results["dtw_peak_assignments"]:

        sim_peak = item["sim_peak"]
        exp_peak = item["matched_exp_peak"]

        sim_y_value = np.interp(sim_peak, x, sim_y)
        exp_y_value = np.interp(exp_peak, x, exp_y)

        ax.plot(
            [exp_peak, sim_peak],
            [exp_y_value, sim_y_value],
            linestyle="--",
            linewidth=GRAPH["match_linewidth"],
            color=GRAPH["match_line_color"],
            alpha=GRAPH["match_line_alpha"]
        )

    ax.tick_params(axis="both", labelsize=GRAPH["tick_fontsize"])
    for tick in ax.get_xticklabels():
        tick.set_fontweight("bold")
    for tick in ax.get_yticklabels():
        tick.set_fontweight("bold")

    if include_legend:
        legend = ax.legend(fontsize=GRAPH["legend_fontsize"])
        for text in legend.get_texts():
            text.set_fontweight("bold")


##########################################################################################
# PLOT 1: ORIGINAL FINGERPRINT SPECTRA
##########################################################################################

fig, ax1 = plt.subplots(
    figsize=GRAPH["figsize"],
    dpi=GRAPH["dpi"]
```

```
)

plot_region_on_axis(ax1, fingerprint_results, include_legend=True)

ax1.set_xlim(FINGERPRINT_REGION[0], FINGERPRINT_REGION[1])

##################################################################################
# REMOVE Y-AXIS TICKS / NUMBERS BUT KEEP LABEL
##################################################################################

ax1.set_yticks([])
ax1.tick_params(axis="y", left=False, labelleft=False)

ax1.spines["top"].set_visible(False)
ax1.spines["right"].set_visible(False)
ax1.spines["left"].set_visible(False)
ax1.spines["bottom"].set_visible(True)

fig.supxlabel(
    "Raman Shift (cm$^{-1}$)",
    fontsize=GRAPH["axis_fontsize"],
    fontweight="bold"
)

ax1.set_ylabel(
    "Vector-Normalized Intensity",
    fontsize=GRAPH["axis_fontsize"],
    fontweight="bold"
)

fig.suptitle(
    GRAPH["overall_original_title"],
    fontsize=GRAPH["axis_fontsize"],
    fontweight="bold"
)

if SAVE_ORIGINAL_FIGURE:
    plt.savefig(ORIGINAL_FIGURE_NAME, dpi=GRAPH["save_dpi"], bbox_inches="tight")

plt.tight_layout()
#plt.show()

##################################################################################
# PLOT 2: DTW-WARPED FINGERPRINT SPECTRA
##################################################################################

fig, ax1 = plt.subplots(
    figsize=GRAPH["figsize"],
```

```
    dpi=GRAPH["dpi"]
)

results = fingerprint_results
alignment_index = np.arange(len(results["warped_exp_y"]))

ax1.plot(
    alignment_index,
    results["warped_exp_y"],
    linewidth=GRAPH["spectrum_linewidth"],
    label="Experimental DTW Path"
)

ax1.plot(
    alignment_index,
    results["warped_sim_y"],
    linewidth=GRAPH["spectrum_linewidth"],
    label="Simulated Warped by DTW"
)

for item in results["dtw_peak_assignments"]:

    exp_idx = item["matched_exp_peak_index"]
    sim_idx = item["sim_peak_index"]

    exp_path_matches = np.where(results["exp_path_indices"] == exp_idx)[0]
    sim_path_matches = np.where(results["sim_path_indices"] == sim_idx)[0]

    if len(exp_path_matches) == 0 or len(sim_path_matches) == 0:
        continue

    exp_warped_idx = exp_path_matches[len(exp_path_matches) // 2]
    sim_warped_idx = sim_path_matches[len(sim_path_matches) // 2]

    ax1.scatter(
        exp_warped_idx,
        results["exp_y"][exp_idx],
        marker="o",
        s=GRAPH["marker_size"],
        color=GRAPH["matched_color"],
        edgecolors=GRAPH["matched_color"],
        linewidths=1.5,
        zorder=5
    )

    ax1.scatter(
        sim_warped_idx,
        results["sim_y"][sim_idx],
```

```
        marker="x",
        s=GRAPH["marker_size"],
        color=GRAPH["matched_color"],
        linewidths=1.5,
        zorder=5
    )

    ax1.plot(
        [exp_warped_idx, sim_warped_idx],
        [results["exp_y"][exp_idx], results["sim_y"][sim_idx]],
        linestyle="--",
        linewidth=GRAPH["warped_match_linewidth"],
        color=GRAPH["match_line_color"],
        alpha=GRAPH["match_line_alpha"]
    )

ax1.tick_params(axis="both", labelsize=GRAPH["tick_fontsize"])

for tick in ax1.get_xticklabels():
    tick.set_fontweight("bold")

for tick in ax1.get_yticklabels():
    tick.set_fontweight("bold")

####################################################################################
# MATCH ORIGINAL FIGURE FORMATTING
####################################################################################

ax1.set_yticks([])
ax1.tick_params(axis="y", left=False, labelleft=False)

ax1.spines["top"].set_visible(False)
ax1.spines["right"].set_visible(False)
ax1.spines["left"].set_visible(False)
ax1.spines["bottom"].set_visible(True)

ax1.set_ylabel(
    "Vector-Normalized Intensity",
    fontsize=GRAPH["axis_fontsize"],
    fontweight="bold"
)

fig.supxlabel(
    "DTW Alignment Index",
    fontsize=GRAPH["axis_fontsize"],
    fontweight="bold"
)
```

```
fig.suptitle(
    GRAPH["overall_warped_title"],
    fontsize=GRAPH["axis_fontsize"],
    fontweight="bold"
)

legend = ax1.legend(fontsize=GRAPH["legend_fontsize"])

for text in legend.get_texts():
    text.set_fontweight("bold")

if SAVE_WARPED_FIGURE:
    plt.savefig(WARPED_FIGURE_NAME, dpi=GRAPH["save_dpi"], bbox_inches="tight")

plt.tight_layout()
#plt.show()
```
